# Phosphatidylethanolamine made in the inner mitochondrial membrane is essential for yeast cytochrome *bc*_1_ complex function

**DOI:** 10.1038/s41467-019-09425-1

**Published:** 2019-03-29

**Authors:** Elizabeth Calzada, Erica Avery, Pingdewinde N. Sam, Arnab Modak, Chunyan Wang, J. Michael McCaffery, Xianlin Han, Nathan N. Alder, Steven M. Claypool

**Affiliations:** 1 Department of Physiology, Johns Hopkins University School of Medicine, Baltimore, MD USA; 2 Department of Molecular and Cell Biology, University of Connecticut, Storrs, CT USA; 3 Barshop Institute for Longevity and Aging Studies, University of Texas Health Science Center at San Antonio, San Antonio, TX USA; 4 Integrated Imaging Center, Department of Biology, Johns Hopkins University, Baltimore, MD USA

## Abstract

Of the four separate PE biosynthetic pathways in eukaryotes, one occurs in the mitochondrial inner membrane (IM) and is executed by phosphatidylserine decarboxylase (Psd1). Deletion of Psd1 is lethal in mice and compromises mitochondrial function. We hypothesize that this reflects inefficient import of non-mitochondrial PE into the IM. Here, we test this by re-wiring PE metabolism in yeast by re-directing Psd1 to the outer mitochondrial membrane or the endomembrane system and show that PE can cross the IMS in both directions. Nonetheless, PE synthesis in the IM is critical for cytochrome *bc*_1_ complex (III) function and mutations predicted to disrupt a conserved PE-binding site in the complex III subunit, Qcr7, impair complex III activity similar to *PSD1* deletion. Collectively, these data challenge the current dogma of PE trafficking and demonstrate that PE made in the IM by Psd1 support the intrinsic functionality of complex III.

## Abstract (web summary)

Phosphatidylethanolamine (PE) is synthesized by four separate pathways, although surprisingly, perturbing mitochondrial PE synthesis compromises mitochondrial function. Here, the authors show that mitochondrial PE synthesis is required for Complex III function and challenge PE trafficking dogma.

## Introduction

The sequestration of enzymes and their substrates into different membrane compartments allows for the enrichment and regulation of metabolite synthesis in regions of the cell where they are essential. In eukaryotes, the essential phospholipid phosphatidylethanolamine (PE) is synthesized by four separate pathways, three of which localize to the endoplasmic reticulum (ER)^1^. A final pathway is dependent on phosphatidylserine decarboxylase (Psd1) which is embedded in the mitochondrial inner membrane (IM)^2–4^. The major PE production pathways are the Kennedy pathway, which synthesizes PE through the stepwise conjugation of CDP-ethanolamine to diacylglycerol, and the Psd pathway, which utilizes phosphatidylserine (PS) as substrate^1^. Deletion of either pathway is lethal during murine embryogenesis, highlighting the importance of PE generation in both the ER and mitochondria^5,6^.

Conservation of the Psd pathway from bacteria to humans suggests that mitochondrial PS/PE metabolism has been preserved to optimize mitochondrial performance^7^. Indeed, deletion of the nuclear-encoded Psd1 (*Pisd* in mammals and *PSD1* in yeast) in eukaryotic cells decreases cellular growth, impairs oxidative phosphorylation (OXPHOS), alters mitochondrial morphology, and diminishes PE levels in cells and mitochondria^5,8–10^. The Psd pathway is the predominant PE production pathway in *Saccharomyces cerevisiae*, producing up to 70% of PE in the cell^11^. Unlike mammals, yeast additionally contain Psd2 which localizes to either Golgi or endosomal compartments^4,12^. Deletion of *PSD2* does not recapitulate the mitochondrial defects associated with loss of *PSD1*^4^. The combined absence of *PSD1* and *PSD2* produces a strain that is auxotrophic for ethanolamine that allows PE synthesis through the Kennedy pathway.

The substrate of Psd1, PS, is synthesized on the mitochondrial-associated membrane (MAM) of the ER by phosphatidylserine synthase (Cho1)^13^. Thus, the amphipathic PS must traverse two aqueous compartments, the cytosol and the mitochondrial intermembrane space (IMS), to reach the IM^14^. Whether a parallel pathway exists for PE import into the IM remains unclear. The lethal consequence of *PISD* deletion in mice and the failure of supplemental ethanolamine to rescue the respiratory defects of *psd1*Δ yeast suggest that PE made outside of the mitochondrion cannot compensate for the absence of Psd1^1,5,10,15^. However, it was recently reported that extra-mitochondrial PE can in fact improve OXPHOS in *psd1*Δ yeast^16^.

Previously, we generated a functional chimeric Psd1 protein in *S. cerevisiae*, ER-Psd1, targeted to the endosomal compartment^17^. In the current study, we further characterize ER-Psd1, together with an OM-targeted chimeric Psd1 (OM-Psd1), to test if the cytosol, IMS, or both, are barriers that prevent non-mitochondrially produced PE from functionally rescuing the absence of PE made in the IM. Alongside strains expressing these re-directed Psd1 constructs, we compare the mitochondrial function of *psd1*Δ*psd2*Δ yeast grown in the non-fermentable carbon source, lactate, with or without exogenous ethanolamine supplementation, to evaluate the ability of the ER-localized Kennedy pathway to support mitochondrial function. Our results establish that PE made outside the IM can indeed access this membrane, but at a significantly reduced abundance in comparison with PE made in the IM by Psd1. This altered PE biogenesis leads to general alterations in mitochondrial phospholipid content, which differentially impacts respiratory complexes III and IV. Our work supports a specific role of IM-synthesized PE in complex III activity, confirmed by site-directed mutations in a known lipid binding pocket of this enzyme. We conclude that in the context of bi-directional PE transport across the IMS, IM-localized Psd1 is required to support electron transport chain activity.

## Results

### Ethanolamine does not fully rescue *psd1*Δ respiratory growth

**Fig. 1:**
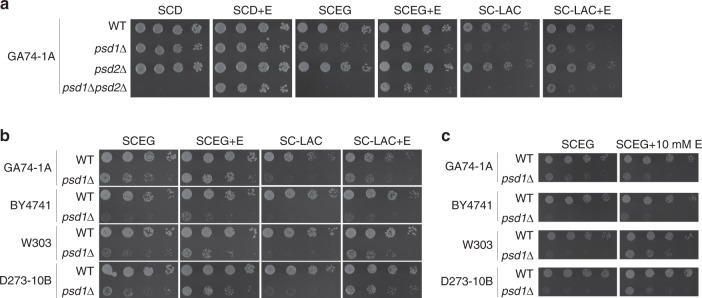
Ethanolamine only partially rescues the respiratory growth defect of *psd1*Δ yeast. The indicated strains were pre-cultured at 30 °C in YPD and spotted onto (**a**, **b**) synthetic complete dextrose (SCD), ethanol-glycerol (SCEG), or lactate medium with or without (+/−) 2 mM ethanolamine (+E) or (**c**) SCEG with or without (+/−) 10 mM ethanolamine and incubated at 30 °C for 2 days (SCD + /− E) or 3 days (all the rest)

The ability of supplemental ethanolamine to rescue the respiratory growth defect of *psd1*Δ yeast has been reported by one group^16^ but not others^1,10,15^. If PE produced by the Kennedy pathway can replace IM-produced PE, this would imply robust trafficking mechanisms to move PE into mitochondria and, by extension, that IM-localized Psd1 is not required for mitochondrial function *per se*. Therefore, we tested the growth of wildtype (WT), *psd1*Δ, *psd2*Δ, and *psd1*Δ*psd2*Δ yeast in synthetic complete ethanol-glycerol (SCEG) or SC-lactate medium with or without ethanolamine supplementation (Fig. 1a). Consistent with previous findings^1,10,15^, we found that ethanolamine restored respiratory growth of *psd1*Δ*psd2*Δ yeast to *psd1*Δ levels but failed to fully restore the respiratory defect of *psd1*Δ yeast. Importantly, this basic result was confirmed in *psd1*Δ yeast from three additional strain backgrounds (Fig. 1b, c), regardless of the amount of ethanolamine provided, although subtle differences between strains were noted. Overall, these findings indicate that the Kennedy pathway cannot fully compensate for the absence of Psd1.

### Validation of Psd1 constructs expressed in the OM or ER

**Fig. 2:**
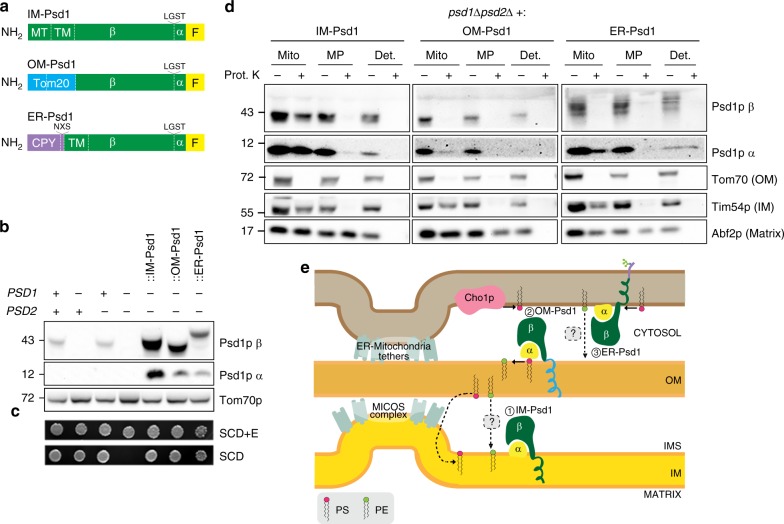
OM-Psd1 and ER-Psd1 constructs are functional and properly mis-localized. **a** Schematic of IM-Psd1, OM-Psd1, and ER-Psd1. All three constructs contain a 3XFLAG tag at the C-terminus (F, yellow). The Tom20 residues (1–100) that replace the mitochondrial targeting sequence (MT) and transmembrane (TM) domain of IM-Psd1 (green) are shown for OM-Psd1 (blue), and the carboxypeptidase Y signal sequence (residues 1–37) as well as an NXS motif are indicated for ER-Psd1 (purple). **b** The β and α subunits of Psd1 were detected in yeast whole cell extracts of the indicated strains by immunoblot. Tom70 served as a loading control. **c** The indicated strains were spotted onto synthetic complete dextrose (SCD) medium with or without (+/−) 2 mM ethanolamine (+E) and incubated at 30 °C for 4 days. **d** Protease protection assay in intact mitochondria (Mito), osmotically ruptured mitochondria (MP), or deoxycholate-solubilized mitochondria (Det.). Following incubation without or with (−/ + ) 100 μg proteinase K (Prot. K) for 30 min, samples were collected, resolved by SDS-PAGE and immunoblotted for Psd1p (β and α subunits), and the mitochondrial compartment-specific markers Tom70 (OM), Tim54 (IM), and Abf2 (matrix). **e** Illustration indicating the topology of (1) IM-Psd1, (2) OM-Psd1, and (3) ER-Psd1

To interrogate whether the cytosol and/or the IMS is a barrier that prevents extra- mitochondrially produced PE from replacing PE made in the IM, we generated chimeric Psd1 constructs that localize to either the ER or OM membranes to redirect PS and PE metabolism (Fig. 2a). Both constructs, and the WT IM-localized Psd1 control (referred to as IM-Psd1 to distinguish it from strains expressing endogenous Psd1), contain a C-terminal 3XFLAG tag to track autocatalytic function of these chimeras by immunodetecting the released Psd1p α subunit. OM-Psd1 and ER-Psd1 each produced mature β and α subunits demonstrating that self-processing remained intact (Fig. 2b). The steady state levels of all three integrated constructs were similar to each other and over-expressed compared to endogenous Psd1. Importantly, both OM-Psd1 and ER-Psd1 rescued the ethanolamine auxotrophy of *psd1*Δ*psd2*Δ yeast indicating that these constructs are capable of synthesizing levels of PE necessary for cellular growth (Fig. 2c).

Previously, ER-Psd1 localization was established by subcellular fractionation and its N-glycosylation status^17^. To confirm the OM localization of OM-Psd1, its protease accessibility was determined in intact mitochondria, OM-ruptured mitoplasts, and detergent-solubilized mitochondria and compared to IM-Psd1. Protease treatment of intact mitochondria expressing IM-Psd1 showed that IM-Psd1, like the IM control Tim54, was protected against degradation (Fig. 2d) while OM-Psd1 was completely degraded like the OM control Tom70, verifying that it was successfully re-localized to the OM facing the cytosol. Given the presence of inter-organelle contact sites, a proportion of ER-Psd1 co-fractionated with crude mitochondria (Supplementary Fig. 1) and demonstrated protease-sensitivity in intact mitochondria (Fig. 2d), a topology that is consistent with its N-glycosylation status (Fig. 2e). Thus, a portion of ER-Psd1 is retained in the ER and/or resides in an endosomal compartment that is co-purified with mitochondria.

### OM-Psd1 and ER-Psd1 mitochondrial PE levels exceed WT

**Fig. 3:**
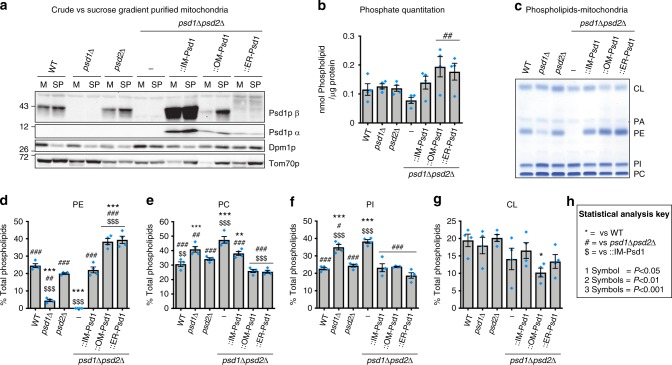
PE is increased in mitochondria from OM-Psd1 and ER-Psd1. **a** Crude mitochondria (M) were purified using a two-step sucrose gradient (SP). Mitochondrial purification was monitored by using the mitochondrial marker Tom70 and the endosomal marker Dpm1. **b** Total phospholipid content/mitochondrial protein (mean ± S.E.M., *n* = 4) in sucrose purified mitochondria. **c** Representative TLC plate of phospholipid extraction from sucrose purified mitochondria followed by visualization using molybdenum blue reagent. **d**–**g** Quantitation of phospholipid levels after separation by TLC and visualization by molybdenum blue staining (mean ± S.E.M., *n* = 4 biologically independent experiments). Statistical comparisons (ns, *P* *>* 0.05; 1 symbol *P* *≤* 0.05; 2 symbols *P* *≤* 0.01; 3 symbols *P* *≤* 0.001) were performed by one-way analysis of variance (ANOVA) with Tukey’s multiple comparison test. **h** Key for symbols used for statistical analysis interpretation when comparing samples *versus* WT (asterisk), *psd1*Δ*psd2*Δ (number sign), or IM-Psd1 (dollar sign)

Next, the lipid content of sucrose gradient purified mitochondria, which none-the-less still contained some co-purified ER (Fig. 3a), was assessed in WT, *psd1*Δ, *psd2*Δ, *psd1*Δ*psd2*Δ, IM-Psd1, OM-Psd1 and ER-Psd1 yeast (Fig. 3b, c). PE was reduced in the absence of Psd1 and undetectable in the combined absence of Psd1 and Psd2 (Fig. 3d). In both *psd1*Δ and *psd1*Δ*psd2*Δ mitochondria, levels of phosphatidylcholine (PC) and phosphatidylinositol (PI) were increased (Fig. 3e, f). Notably, *PSD2* deletion modestly decreased mitochondrial PE (Fig. 3d) but did not result in a respiratory growth defect (Fig. 1a). Combined, these results indicate that Psd2 contributes to the mitochondrial-associated pool of PE but is unable to functionally replace PE made by Psd1.

Interestingly, OM-Psd1 and ER-Psd1 yeast contained significantly higher amounts of PE in mitochondrial-enriched membranes compared to IM-Psd1, which maintained levels similar to WT (Fig. 3d). As IM-Psd1, OM-Psd1, and ER-Psd1 are similarly over-expressed (Supplementary Fig. 2A−C), this suggests that OM-Psd1 and ER-Psd1 have increased access to their substrate and short-circuited normal mitochondrial PS/PE metabolism. OM-Psd1 and ER-Psd1 both normalized the absolute amount of Cho1 and its phosphorylated pool which were significantly increased in *psd1*Δ*psd2*Δ yeast grown in respiratory conditions (Supplementary Fig. 2A, and 2D−F). The relative abundance of PC was reduced in OM-Psd1 and ER-Psd1 (Fig. 3e). Intriguingly, the CL levels in OM-Psd1 mitochondria were significantly decreased compared to WT (Fig. 3g). The reduced levels of PC in OM-Psd1 and ER-Psd1 is notable as it might have been predicted that an increased production of PE would have resulted in augmented PC synthesis by ER-resident methyltransferases that convert PE to PC^18^. The steady state level of Kar2, the yeast equivalent of the Hsp70 chaperone BiP^19^, was not increased in OM-Psd1 or ER-Psd1 (Supplementary Fig. 2g), demonstrating that their altered membrane compositions did not induce ER stress. In contrast, Kar2 was significantly elevated in both *psd1*Δ*psd2*Δ and *cho1*Δ strains. Importantly, there were no dramatic differences in the mitochondrial architectures of strains with elevated or decreased PE levels (Supplementary Fig. 3), which is consistent with previous observations in *psd1*Δ yeast^16^. Overall, re-routing Psd1 to either the OM or ER results in a robust increase in mitochondrial-associated PE levels which may or may not reach the IM.

### OM-Psd1 and ER-Psd1 phenocopy the *psd1*Δ respiratory defect

**Fig. 4:**
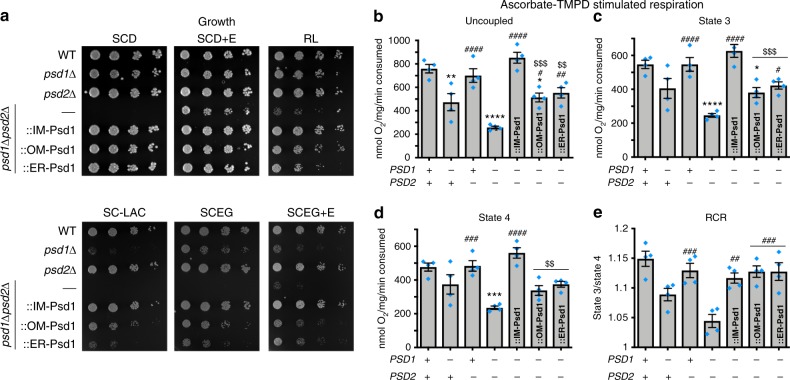
OM-Psd1 and ER-Psd1 OXPHOS function phenocopies *psd1*Δ. **a** The indicated strains were spotted and incubated at 30 °C for 2 days on SCD with or without (+/−) 2 mM ethanolamine (+E) and for 5 days on rich lactate (RL), SC lactate (SC-LAC), and SCEG with or without (+/−) 2 mM ethanolamine (+E). **b**–**e** O_2_ consumption measurements from mitochondria isolated from yeast grown in rich lactate using ascorbate-TMPD as a substrate. **b** The maximal respiratory rate was recorded after the addition of CCCP, (**c**) state 3 respiration was assessed after addition of ADP, and (**d**) state 4 respiration was recorded following ADP depletion. **e** The respiratory control ratio (RCR) is calculated by dividing state 3 by state 4 respiratory rates. Statistical comparisons (ns, *P* *>* 0.05; 1 symbol *P* *≤* 0.05; 2 symbols *P* *≤* 0.01; 3 symbols *P* *≤* 0.001; 4 symbols *P* *≤* 0.0001) vs. WT (asterisk), *psd1*Δ*psd2*Δ (number sign), or IM-Psd1 (dollar sign) were performed by one-way ANOVA with Tukey’s multiple comparison test (mean ± S.E.M. for *n* = 4 biologically independent experiments)

OXPHOS was initially evaluated in these strains by determining their growth on synthetic media containing dextrose with or without (+/−) ethanolamine, lactate, or ethanol-glycerol + /− ethanolamine (Fig. 4a). Compared to IM-Psd1, OM-Psd1 and ER-Psd1 only partially improved growth of *psd1*Δ*psd2*Δ yeast on respiratory carbon sources (lactate and ethanol-glycerol) and growth was not improved by ethanolamine (Fig. 4a). This suggests that PE made in either the OM or ER cannot functionally replace PE produced in the IM.

To directly assess OXPHOS capacity in these strains, oxygen consumption was monitored in mitochondria using an O_2_ electrode after the addition of ADP and ascorbate tetramethyl-*p*-phenyldiamine (TMPD) which promotes proton pumping by complex IV (Figs. 4b–e). The ADP-stimulated (State 3) respiratory rate is recorded after addition of ascorbate TMPD plus ADP and is calculated as the amount of O_2_ consumed over time. ADP facilitates proton pumping and the O_2_ consumption rate and upon its depletion by complex V, the respiratory rate returns to a resting state (State 4). Maximal respiratory capacity was evaluated by addition of carbonyl cyanide *m*-chlorophenyl hydrazine (CCCP), a proton ionophore that collapses the electrochemical gradient across the IM making it easier to pump protons.

Mitochondria lacking Psd1, but not Psd2, had reduced maximal respiratory rate compared to WT mitochondria. Even though *psd2*Δ mitochondria consumed O_2_ like WT, the combined absence of Psd1 and Psd2 caused a more severe respiratory defect than when only Psd1 was missing. This indicates that in the absence of Psd1, PE made by Psd2 has some capacity to support respiratory activity. The uncoupled respiratory rate for OM-Psd1 and ER-Psd1 was significantly increased compared to *psd1*Δ*psd2*Δ mitochondria but still impaired relative to IM-Psd1 (Fig. 4b). The respiratory control ratio (RCR) is an indication of how well proton pumping by the electron transport machinery is coupled to ATP synthesis and is calculated by dividing the ADP-stimulated respiration rate by the resting respiration rate (State 3/State 4). A decrease in the rate of State 3 over State 4 would suggest defective coupling in these processes possibly due to proton leak. RCR ratios were significantly decreased in *psd1*Δ and *psd1*Δ*psd2*Δ mitochondria but perplexingly, OM-Psd1 and ER-Psd1 displayed a ratio similar to WT and IM-Psd1 (Fig. 4c). For *psd1*Δ*psd2*Δ yeast expressing OM-Psd1 and ER-Psd1, the normal RCR stems from the fact that they increased State 3 O_2_ consumption more than State 4. Similar to Psd2 in the context of *psd1*Δ yeast, OM-Psd1 and ER-Psd1 significantly increased *psd1*Δ*psd2*Δ respiratory rates to roughly *psd1*Δ levels, indicating that extra-mitochondrial PE can indeed enhance respiration. Combined, these results suggest that defective complex IV function may contribute to the reduced respiratory growth observed when Psd1 is absent from the IM.

### The complex IV defect in *psd1*Δ*psd2*Δ is rescued by ER-Psd1

**Fig. 5:**
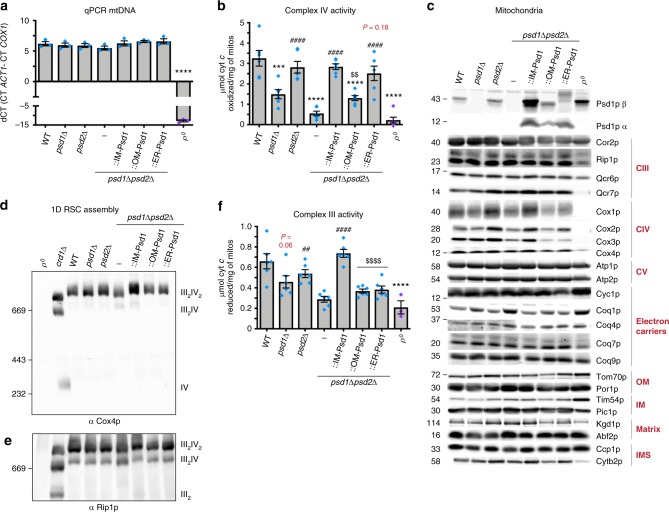
Complex III and IV activities are impaired when Psd1 is absent in the IM. **a** mtDNA was isolated from the indicated strains, normalized, and quantified by qPCR (mean ± S.E.M. for *n* = 3 biologically independent experiments). Analysis was performed by one-way ANOVA with Tukey’s multiple comparison test. **b** Complex IV activity in isolated mitochondria solubilized in 0.5% (w/v) DDM (mean ± S.E.M. for *n* = 6 biologically independent experiments, except for ρ^0^, *n* = 3). **c** Mitochondria from the indicated strains were immunoblotted for subunits of complex III (CIII), complex IV (CIV), complex V (CV), the Coq synthome, cytochrome *c*, and markers of each mitochondrial compartment. **d**, **e** Blue native-PAGE analysis of respiratory supercomplexes (RSCs) using mitochondrial extracts solubilized in 1.5% (w/v) digitonin. **d** Complex IV assembly was monitored by immunoblot against the nuclear-encoded subunit Cox4 and (**e**) Complex III assembly was monitored by immunoblot against the nuclear-encoded subunit Rip1. Mitochondria lacking CL (*crd1*Δ) were used as a positive control for RSC destabilization^53^. **f** Complex III activity in isolated mitochondria solubilized in 0.5% (w/v) DDM (mean ± S.E.M. for *n* = 6 biologically independent experiments, except for ρ^0^, *n* = 3). In B and F, statistical comparisons (ns, *P* *>* 0.05; 1 symbol *P* *≤* 0.05; 2 symbols *P* *≤* 0.01; 3 symbols *P* *≤* 0.001; 4 symbols *P* *≤* 0.0001) *versus* WT (asterisk), *psd1*Δ*psd2*Δ (number sign), or IM-Psd1 (dollar sign) were performed by one-way ANOVA with Tukey’s multiple comparison test. *P* values for decreases that didn’t achieve significance are reported in red and were analyzed by student *t*-test versus WT

When grown in dextrose, *psd1*Δ and *psd1*Δ*psd2*Δ yeast lose their mitochondrial genome at a high frequency^1,16^. Due to this, we harvested mitochondria from cultures grown in rich lactate to select for respiratory competent cells. As expected, mitochondrial DNA (mtDNA) levels were equivalent between strains in these growth conditions (Fig. 5a; ρ^0^ yeast devoid of mtDNA served as a negative control). Next, isolated complex IV activity was determined in *n*-Dodecyl-β-D-maltoside (DDM)-solubilized mitochondria. Complex IV activity was significantly decreased in *psd1*Δ and *psd1*Δ*psd2*Δ mitochondria as previously reported^8,16^, as well as in OM-Psd1 mitochondria but surprisingly, ER-Psd1 retained WT function (Fig. 5b). To determine if the different complex IV activities associated with OM-Psd1 and ER-Psd1 mitochondria reflected changes in its expression, we analyzed the steady state amounts of both nuclear and mtDNA-encoded subunits of complex IV (Fig. 5c). While the levels of the mtDNA-encoded subunit, Cox2, was significantly decreased in *psd1*Δ*psd2*Δ and OM-Psd1 strains, the steady state abundance of the two additional mtDNA-encoded subunits, Cox1 and Cox3, trended towards a decrease in *psd1*Δ, *psd1*Δ*psd2*Δ and OM-Psd1 that did not quite reach statistical significance (Supplementary Fig. 4). Additionally, the amount of a constituent encoded in the nucleus, Cox4, was decreased in OM-Psd1 but not *psd1*Δ*psd2*Δ. Blue native-PAGE analyses indicated that complex IV assembly into respiratory supercomplexes (RSCs) that consist of a complex III dimer affiliated with either one or two complex IV monomers^20^, was normal regardless of the absence of *PSD1* or *PSD2*, singly or in combination, as reported by others^16^, or whether Psd1 was expressed in the IM, OM, or ER (Fig. 5d).

### Reduced complex III activity when Psd1 is not in the IM

The ability of ER-Psd1, but not OM-Psd1, to rescue complex IV activity to WT levels was surprising given that neither chimeric construct restored respiratory growth of the *psd1*Δ*psd2*Δ strain to this degree (Fig. 4a). Therefore, we postulated that the incomplete respiratory growth rescue of OM-Psd1 and ER-Psd1 could reflect a defect in complex III. Indeed, complex III activity was reduced in *psd1*Δ and significantly decreased in *psd1*Δ*psd2*Δ mitochondria (Fig. 5f). While ER-Psd1 significantly improved complex IV function compared to *psd1*Δ*psd2*Δ mitochondria, neither ER-Psd1 nor OM-Psd1 restored complex III activity to WT levels. The reduced complex III activity in *psd1*Δ, *psd1*Δ*psd2*Δ, and *psd1*Δ*psd2*Δ expressing OM-Psd1 or ER-Psd1 did not reflect alterations in the steady state abundance of its subunits (Fig. 5c, Cor2, Rip1, Qcr6, and Qcr7, quantified in Supplementary Fig. 4) or its assembly into supercomplexes (Fig. 5e), although there was proportionately more of the smaller supercomplex (III_2_IV > III_2_IV_2_) detected in *psd1*Δ*psd2*Δ mitochondria. Furthermore, the steady state levels of the complex III electron acceptor cytochrome *c* were normal (Fig. 5c and Supplementary Fig. 4M). Similarly, subunits of the coenzyme Q (CoQ) synthome, a macromolecular complex that catalyzes the synthesis of the complex III electron donor CoQ^21^, were equal with one exception (Fig. 5C and Supplementary Fig. 5). In *psd1*Δ*psd2*Δ mitochondria, Coq1 was increased which could represent an attempt to diminish membrane stress^22^. Moreover, CoQ_6_ supplementation, which is capable of rescuing strains with reduced CoQ biosynthesis^21^, failed to improve respiratory growth of *psd1*Δ or *psd1*Δ*psd2*Δ yeast (Supplementary Fig. 6). Lastly, *psd1*Δ, *psd1*Δ*psd2*Δ, OM-Psd1, and ER-Psd1 respiratory growth was not further impaired in medium lacking *para*-amino benzoic acid (Supplementary Fig. 7), a molecule that can be used to produce CoQ by a secondary pathway^23^. In total, our results demonstrate that CoQ is not limiting for respiratory function in any of these strains. As such, they favor the hypothesis that the impaired complex III activity of *psd1*Δ, *psd1*Δ*psd2*Δ, OM-Psd1, and ER-Psd1 is intrinsic to the multi-subunit holoenzyme itself.

### Altered IM phospholipid profile with disrupted PE metabolism

**Fig. 6:**
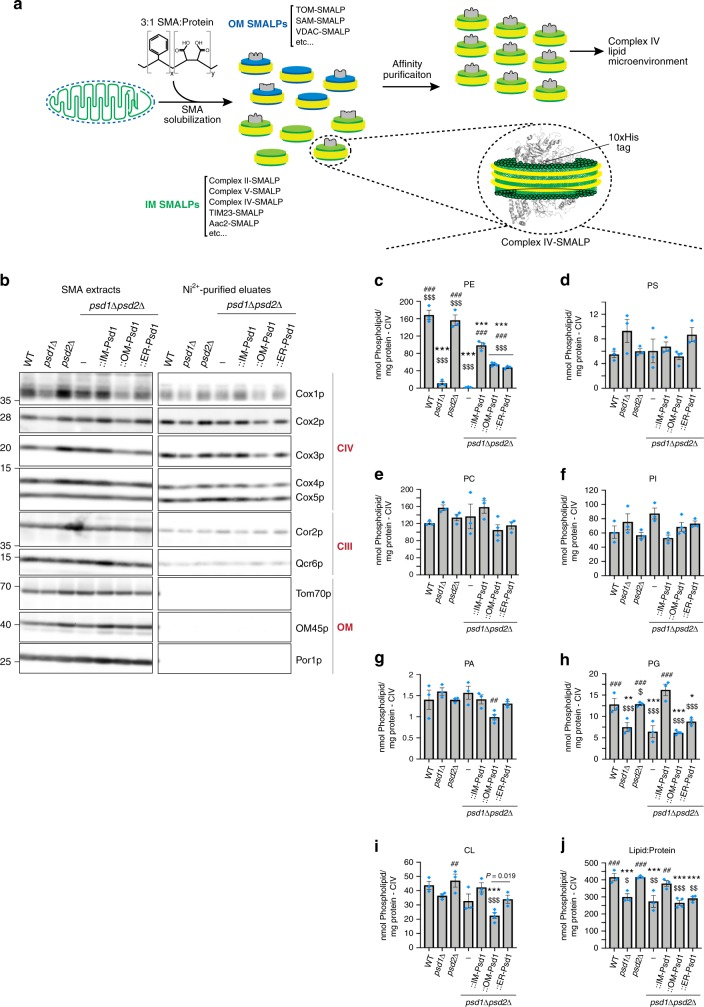
PE made in the OM or ER is incorporated into the IM. **a** Schematic of purification of complex IV nanodiscs from SMA-extracted mitochondria. **b** Following SMA-solubilization of osmotically ruptured mitochondria isolated from the indicated Cox8-His expressing strains, complex IV was affinity purified with Ni-agarose and bound material recovered by elution with 60 mM Imidazole. Unpurified SMA extracts and Ni^2+^-purified complex IV eluates (200 ng each) were resolved by SDS-PAGE and immunoblotted as indicated. **c**–**j** The phospholipid composition of complex IV nanodiscs was determined by shotgun lipidomics. The amount (nmol phospholipid/mg complex IV protein) of (**c**) PE, (**d**) PS, (**e**) PC, (**f**) PI, (**g**) phosphatidic acid (PA), (**h**) PG, (**i**) CL, and (**j**) the total phospholipid:protein ratio was determined (mean ± S.E.M. for *n* = 3 biologically independent experiments except for OM-Psd1, *n* = 4). Analysis *versus* WT (asterisk), *psd1*Δ*psd2*Δ (number sign), or IM-Psd1 (dollar sign) was performed by one-way ANOVA with Holm–Sidak pairwise comparisons

Elevated mitochondrial PE levels have been suggested to be toxic for mitochondrial cristae morphology and respiratory function^24^. Therefore, given the increased levels of PE associated with OM-Psd1 and ER-Psd1 mitochondria (Fig. 3d), we sought to determine the IM lipid composition in our panel of yeast strains. As such, we adapted a detergent-free method that exploits the ability of styrene maleic acid (SMA) copolymers to extract membrane protein complexes and their adjacent phospholipid microenvironment in nanodics^25,26^. It was previously shown that complex IV affinity purified from SMA-extracted mitochondria contains the major IM phospholipids^27^ (Fig. 6a). Since complex IV is inarguably a resident of the IM, we reasoned that its local phospholipid environment will provide insight into the local lipid milieu surrounding the RSCs, which may reflect the overall IM phospholipid composition. To this end, we CRISPR-engineered a His tag onto the C-terminus of endogenous Cox8. Cox8-His was expressed, functional, and assembled normally into RSCs (Supplementary Fig. 8A–C). Affinity purification of SMA extracts with Ni-agarose was specific for Cox8-His, resulted in co-purification of additional complex IV subunits, and yielded a population of SMA-stabilized discoidal nanoparticles (Supplementary Fig. 8D–E).

We therefore appended a His tag at the C-terminus of Cox8 in our panel of re-wired Psd1 yeast and associated control strains (Supplementary Fig. 8F). A preparative scale Ni-agarose affinity purification was performed from SMA-extracted mitochondria and the resulting eluates were enriched in subunits of complex IV (Fig. 6b). While some complex III subunits were also present in the purified complex IV nanodiscs, OM proteins (Tom70, OM45, and Por1) were not detected (Fig. 6b). Thus, the purified complex IV nanodiscs are devoid of OM contaminants. Next, the phospholipid composition of the purified complex IV nanodiscs was determined by shotgun lipidomics (Fig. 6c–j). The amount of PE that co-purified with complex IV nanodiscs in the absence of Psd1 was even less than what was detected in gradient purified mitochondria (Figs. 6c and 3d, 6.7% vs 22% relative to WT PE levels, respectively). While PE was increased by 156% in mitochondria from OM-Psd1 and ER-Psd1 compared to WT, it was reduced by ~70% in their purified complex IV nanodiscs. This indicates that although PE made in the OM or ER can be transported across the IMS and incorporated into the IM, much of it remains in the OM and/or co-purified ER membranes. Surprisingly, the levels of PE, which were normal in mitochondria (Fig. 3d), was reduced by 59% in complex IV nanodiscs purified from IM-Psd1. While the basis for this observation is unclear, the fact that IM-Psd1 functionally rescued the absence of endogenous Psd1 indicates that the amount of PE in the IM is sufficient, at least for the IM functions tested. With the exception of phosphatidylglycerol (PG), the complex IV nanodisc-associated levels of other phospholipids were similar regardless of the absence of *PSD1* or *PSD2*, singly or in combination, or whether Psd1 was expressed in the IM, OM, or ER. However, differences in the acyl chain profile of phospholipids associated with complex IV nanodics were identified that varied depending on if and where PE was made (Supplementary Fig. 9). Unexpectedly, the amount of complex IV nanodisc-associated PG, a precursor for CL that is of low abundance, was decreased when Psd1 was not in the IM (Fig. 6h). Another CL precursor, PA, was reduced in complex IV purified nanodiscs from OM-Psd1 (Fig. 6g), which also contained significantly less CL (Fig. 6i). Notably, when Psd1 is not present in the IM, the purified complex IV nanodiscs had a significantly lower phospholipid:protein ratio (Fig. 6j). In the absence of a clear compensatory increase in another lipid class, this is likely a direct consequence of their low PE levels. Collectively, our results demonstrate that while PE made in the OM or ER can access the IM, it fails to accumulate to the level needed to fully support complexes III and IV function.

### Ethanolamine rescues complex IV but not complex III activity

**Fig. 7:**
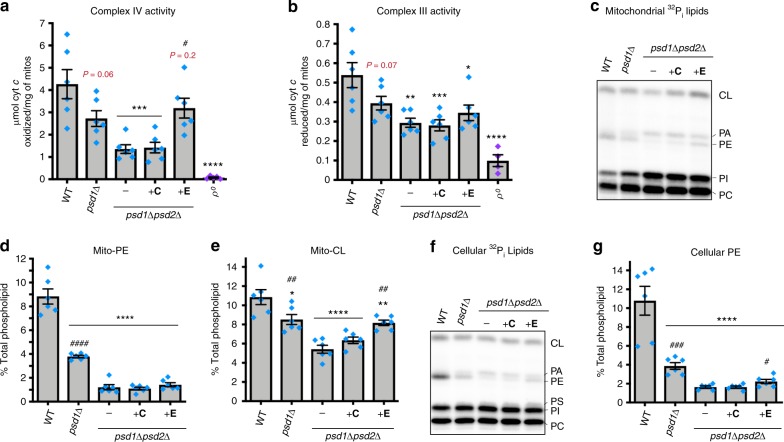
Ethanolamine rescues the activity of complex IV but not complex III. The indicated yeast strains were grown in rich lactate without (WT, *psd1*Δ, and *psd1*Δ*psd2*Δ) or with choline (+C) or ethanolamine (+E). **a** Complex IV activity and (**b**) complex III activity in isolated mitochondria solubilized in 0.5% (w/v) DDM (mean ± S.E.M. for *n* = 6 biologically independent experiments, except for ρ^0^, *n* = 4). Statistical comparisons (ns, *P* *>* 0.05; 1 symbol *P* *≤* 0.05; 2 symbols *P* *≤* 0.01; 3 symbols *P* *≤* 0.001; 4 symbols *P* *≤* 0.0001) vs. WT (asterisk) or *psd1*Δ*psd2*Δ (number sign) were performed by one-way ANOVA with Tukey’s multiple comparison test. *P* values for decreases that did not achieve significance are reported in red and were analyzed by student *t* test *versus* WT. **c**–**e** Mitochondrial phospholipids from the indicated strains were labeled overnight with ^32^P_i_ and separated by TLC. **c** Representative TLC plate for mitochondrial ^32^P_i_ lipids. Quantitation of mitochondrial (**d**) PE and (**e**) CL levels (mean ± S.E.M. for *n* = 6 biological replicates). Significant differences compared to WT (asterisk) or *psd1*Δ*psd2*Δ (number sign) were calculated by one-way ANOVA with Holm–Sidak pairwise comparisons. **f** Representative TLC plate for cellular ^32^P_i_ lipids. **g** Quantitation of cellular PE levels (mean ± S.E.M. for *n* = 6 biological replicates). Significant differences compared to WT (asterisk) or *psd1*Δ*psd2*Δ (number sign) were calculated by student *t*-test

As ER-Psd1 restores complex IV, but not complex III, function, we isolated *psd1*Δ*psd2*Δ mitochondria from cultures grown in rich lactate without or with (−/ + ) choline or ethanolamine, to evaluate the impact of PC and PE generation by the Kennedy pathways on respiratory activity (Fig. 7a, b). Similar to ER-Psd1, ethanolamine, but not choline, restored *psd1*Δ*psd2*Δ complex IV (Fig. 7a), but not complex III (Fig. 7b), function to WT levels. Combined, our results indicate that PE, but not PC, made in the ER by the Kennedy pathway (Fig. 7A) or in the endosomal system by either Psd2 or ER-Psd1 (Fig. 5b), can significantly rescue the severe complex IV dysfunction that occurs in *psd1*Δ*psd2*Δ yeast. Surprisingly, when we evaluated the phospholipid composition of *psd1*Δ*psd2*Δ yeast supplemented with choline or ethanolamine, we found that ethanolamine did not alter mitochondrial PE levels (Fig. 7c, d) and only modestly and yet significantly increased cellular PE abundance (Fig. 7f, g), consistent with the slight incre ases observed in^1,28^ but not^16^. Intriguingly, ethanolamine, but not choline, supplementation significantly increased CL in *psd1*Δ*psd2*Δ yeast to *psd1*Δ levels (Fig. 7e). Significant changes in the abundance of other phospholipid species in *psd1*Δ*psd2*Δ yeast supplemented with either choline or ethanolamine were not observed (Supplementary Fig. 10). As such, these results indicate that the Kennedy Pathway for PE production is metabolically linked to CL biosynthesis and/or stability. Moreover, they suggest that the ability of ethanolamine to improve complex IV activity in *psd1*Δ*psd2*Δ yeast coincides with its unanticipated capacity to increase CL levels, a phospholipid known to be important for complex IV function^29^. These findings demonstrate that both CL and PE are important for complex IV activity. Moreover, they further underscore that PE made within the IM is necessary for the full activity of complex III which is otherwise normally expressed, fully assembled, and not limited by the amount of either of its mobile electron carriers.

### PE synthesis by Psd1 is required for complex III activity

**Fig. 8:**
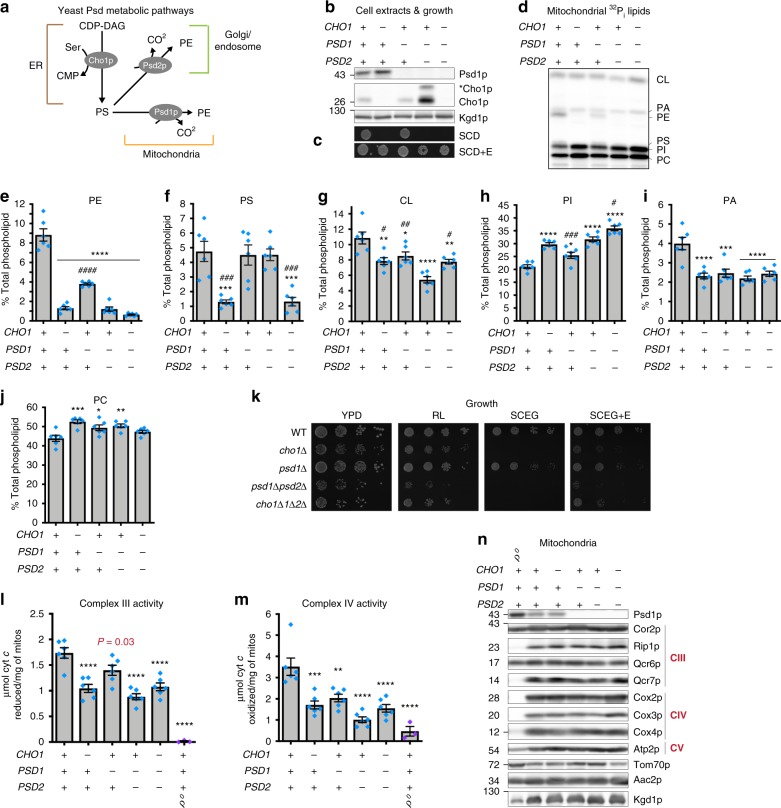
Deletion of Cho1 impairs complex III and complex IV activities. **a** Metabolic pathways tied to PS synthesis by Cho1 in yeast. **b** Detection of the β subunit of Psd1 and Cho1 expression were verified in yeast whole cell extracts of the indicated strains by immunoblot. Kgd1 served as a loading control. *Cho1, phosphorylated Cho1. **c** The indicated strains were spotted onto synthetic complete dextrose (SCD) medium with or without (+/−) 2 mM ethanolamine (+E) and incubated at 30 °C for 2 days. **d**–**j** Mitochondrial phospholipids from the indicated strains were labeled overnight with ^32^P_i_, separated by TLC, and quantitated by phosphoimaging (mean ± S.E.M. for *n* = 6 biological replicates). Significant differences compared to WT (asterisk) or *psd1*Δ*psd2*Δ (number sign) were calculated by one-way ANOVA with Holm–Sidak pairwise comparisons. **k** The indicated strains were spotted and incubated at 30 °C for 2 days on YPD and for 3 days on rich lactate (RL), and SCEG without or with 2 mM ethanolamine (+E). **l** Complex III activity and (**m**) complex IV activity in isolated mitochondria solubilized in 0.5% (w/v) DDM (mean ± S.E.M. for *n* = 6 biologically independent experiments, except for ρ^0^, *n* = 3). Analysis vs. WT by one-way ANOVA with Tukey’s multiple comparison test. **n** Steady state expression of mitochondrial proteins in mitochondria isolated from the indicated strains

Cho1 produces PS in the MAM of the ER through conjugation of free serine with CDP-diacylglycerol^13^. In yeast, this feeds into both the Psd1 and Psd2 PS decarboxylation pathways (Fig. 8a). We generated *CHO1* deletion strains in WT and *psd1*Δ*psd2*Δ (labeled *cho1*Δ*1*Δ*2*Δ in Fig. 8k) yeast to deplete mitochondrial PS/PE levels while preserving Psd1 expression. Consistent with previous data^17^, deletion of Cho1 did not affect Psd1 accumulation or maturation (Fig. 8b) and resulted in an ethanolamine auxotrophy^30^ (Fig. 8c). As anticipated, PS and PE levels were drastically reduced in strains lacking Cho1 (Fig. 8d–f). In comparison to *psd1*Δ*psd2*Δ, *cho1*Δ*psd1*Δ*psd2*Δ yeast had a significant increase in CL and PI levels (Fig. 8g, h), the former of which may be associated with its enhanced respiratory growth (Fig. 8k). Deletion of *CHO1* in the *psd1*Δ*psd2*Δ background restored PC to WT levels (Fig. 8j) but failed to increase the levels of the CL precursor PA (Fig. 8i). Growth of *cho1*Δ was decreased compared to WT in YPD and rich lactate media and similar to *psd1*Δ*psd2*Δ in SCEG containing ethanolamine (Fig. 8k). Notably, the activities of complexes III (Fig. 8l) and IV (Fig. 8m) were reduced in the absence of Cho1. Since Psd1 and essential subunits of complexes III and IV were expressed normally in the absence of Cho1, singly or in combination with Psd1 and Psd2 (Fig. 8n), our combined results directly implicate mitochondrial PE depletion as the cause for the reduced respiratory function of *psd1*Δ, *psd1*Δ*psd2*Δ, and *cho1*Δ yeast.

### Glu82 of Qcr7 may coordinate PE associated with complex III

**Fig. 9:**
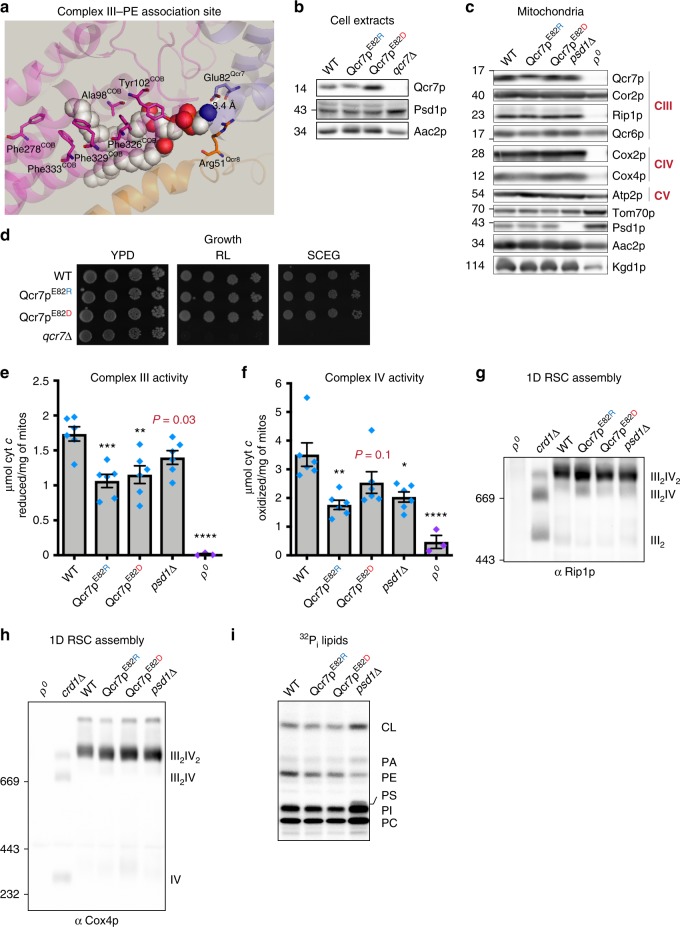
A PE-coordinating residue in Qcr7 is important for complex III activity. **a** The crystal structure of yeast cytochrome *bc*_1_ that modeled associated lipids was downloaded using PDB ID: 1KB9. Using PyMOL, the region containing the catalytic subunit Cob1 (magenta) near the matrix-facing surface was enlarged to demonstrate hydrophobic interactions between this subunit and the acyl chains of PE. Arg51 of Qcr8 (orange) also shows hydrophobic interactions with a carbon atom from the ethanolamine headgroup. Glu82 of Qcr7 (blue) was predicted to form a hydrogen bonding interaction (3.4 Å distance) with the amine group of PE, whose atoms are depicted as spheres; gray: carbon, red: oxygen, and blue: nitrogen (hydrogen atoms are not represented). **b** WT and mutant Qcr7 was detected in yeast whole cell extracts of the indicated strains by immunoblot; Aac2 served as a loading control. **c** Mitochondria from the indicated strains were immunoblotted for subunits of complex III and complex IV as well as markers for the indicated mitochondrial compartments. **d** The indicated strains were spotted and incubated at 30 °C for 2 days on YPD and for 3 days on rich lactate (RL) and SCEG. **e** Complex III activity or (**f**) complex IV activity in isolated mitochondria solubilized in 0.5% (w/v) DDM (mean ± S.E.M. for *n* = 6 biologically independent experiments, except for ρ^0^, *n* = 3).. Analysis *versus* WT (asterisk) was performed by one-way ANOVA with Tukey’s multiple comparison test. *P* values for decreases that did not achieve significance are reported in red and were analyzed by student *t*-test *versus* WT. The assembly of (**g**) complex III and (**h**) complex IV was monitored by immunoblot against the nuclear-encoded subunits Rip1 and Cox4, respectively. Mitochondria lacking RSCs (ρ^0^) or CL (*crd1*Δ) were used controls. **i** Mitochondrial phospholipids from the indicated strains were labeled overnight with ^32^P_i_ and separated by TLC

PE and CL were identified in crystal structures of the yeast and mammalian cytochrome *bc*_1_ complex in association with the essential mtDNA-encoded catalytic subunit cytochrome *b* (Cob1) as well as the nuclear-encoded subunit Qcr7^31,32^. Qcr7 is associated with the matrix-facing surface of Cob1 and it is postulated that hydrogen bonding interactions between the headgroup of PE and Glu82 of Qcr7 may help position the complex vertically within the bilayer (Fig. 9a). To test the importance of this residue in forming hydrogen bonds with the amine group of PE, we introduced a charge reversal by mutating Glu82 to Arg and also created a strain expressing Asp82 to test the potential effect of shortening the distance of this interaction. Importantly, the amount of Qcr7^E82R^ or Qcr7^E82D^ in cell and mitochondrial lysates (Fig. 9b, c) was similar to WT (the Qcr7^E82R^ variant was consistently upshifted compared to WT following SDS-PAGE). Further, Qcr7^E82R^ and Qcr7^E82D^ supported respiratory growth in rich or minimal medium in contrast to *qcr7*Δ whose respiratory growth was compromised (Fig. 9d). Despite being sufficiently functional to promote respiratory growth, complex III activity was significantly decreased for Qcr7^E82R^ and Qcr7^E82D^ to a similar degree as when Psd1 is missing (Fig. 9e). Surprisingly, complex IV activity was also decreased in Qcr7^E82R^ but not Qcr7^E82D^ (Fig. 9f). The impaired respiratory complex activity for Qcr7^E82R^ and Qcr7^E82D^ was independent of any changes in the steady state amount of their phospholipids, subunits, or subunit assembly into RSCs (Fig. 9c and g–i). These results provide the first molecular evidence of the functional importance of a conserved PE-binding site identified in the structures of yeast and human complex III. Collectively, these data demonstrate that PE made in the IM by Psd1 is critical to support the intrinsic functionality of complex III and suggest one likely mechanism.

## Discussion

The results derived from this study support four important conclusions. First, PE flux across the IMS is bi-directional. Given the capacity of Psd1 to produce up to 70% of PE in the cell^11^, it has long been appreciated that PE made in the IM can be transported across the IMS to other membrane-bound compartments. Our biochemical determination that PE made in the OM or ER can access the IM demonstrates that the mechanism(s) responsible for PE transport across the IMS, which has not yet been molecularly identified, can do so in both directions. This ability again raises the question as to why there is a PE-generating enzyme localized to the mitochondrial IM. In this regard, it is notable that the levels of PE in the IM, or at least in the immediate vicinity of complex IV, are significantly lower when Psd1 makes robust amounts of PE in either the OM or ER. Moreover, the resulting amount of PE in the IM is unable to fully support the activities of complex IV and especially complex III. These observations support a model whereby the final distribution of PE between the IM and OM reflects a thermodynamic equilibrium between a high local gradient of PE in the IM established by Psd1 and PE transport across the IMS, which is in principle bi-directional. According to this model, the presence of Psd1 in the IM ensures that the levels of PE in this membrane are sufficient to fully support the activity of the respiratory complexes.

Second, the absence of PE made in the IM results in severe complex III and IV functional defects. Interestingly, PE made in the ER by ER-Psd1 or the Kennedy pathway upon ethanolamine supplementation can rescue complex IV but not complex III function in *psd1*Δ*psd2*Δ yeast. What is the basis for the capacity of extra-mitochondrially produced PE to rescue the activity of complex IV but not complex III? While the answer to this question remains elusive, it is notable that CL, and not PE, was increased upon ethanolamine supplementation (Fig. 7e), a phenomenon that has been observed previously^1,16^, suggesting that this rescue may be CL-dependent. Support for this possibility also stems from the failure of OM-Psd1, which has even lower levels of CL than *psd1*Δ*psd2*Δ (Figs. 3g and 6j), to increase the activity of complex IV (Fig. 5b). The importance of CL in the assembly and function of the RSCs is well documented^29,33,34^. Although it is presently unclear how PE production in the ER promotes CL accumulation, it is known that the Ups1 and Ups2 lipid trafficking proteins have an inverse relationship with respect to CL/PE metabolism suggesting that this may be linked to PS/PA trafficking into the IM^3,35,36^. Depletion of PS in the *psd1*Δ*psd2*Δ background restored CL levels to that of *psd1*Δ and *cho1*Δ which were still comparatively reduced *vs* WT (Fig. 8g). This increase in CL also coincided with improved growth for *cho1*Δ*psd1*Δ*psd2*Δ yeast (Fig. 8k). It is possible that in the absence of PS, metabolic pathways that either promote PA formation in the ER^37^ or promote PA import to the IM^38,39^ are stimulated. Recent evidence has also implicated ethanolamine in improving the respiratory function of CL-depleted strains^40^. As respiration and complex assembly are improved in the absence of CL but coincides with increases in PG/PE levels, this supports redundant functions of PE/CL for mitochondrial respiration. Moving forward, it will be important to distinguish between these non-mutually exclusive models.

Third, PE made by Psd1 in the IM is especially important for complex III function. Since *psd1*Δ*psd2*Δ yeast contain normal amounts of cytochrome *c* and CoQ_6_ is not limiting, the underlying respiratory defect is intrinsic to complex III. As both complex III and complex IV are IM residents, one possibility is that these respiratory complexes require different levels of PE within the IM to function appropriately. However, the levels of complex IV-associated PE were the same for OM-Psd1 and ER-Psd1 (Fig. 6d). Another intriguing possibility is that the synthesis of PE by Psd1 in the IM is somehow directly coupled to the incorporation of this lipid into partially or fully assembled complex III. Recently, a subunit of complex IV was found to associate with the mitochondrial contact site and cristae organizing system (MICOS) subunit, Mic19, in mammalian cells by EM tomography and immunoprecipitation^41^. As the MICOS complex was suggested to work in concert with Ups2 to regulate mitochondrial PS/PE metabolism in yeast^24^, contact sites between the OM and IM could potentially facilitate the transport of ER-derived PE to complex IV more directly than it can to complex III.

Finally, we demonstrated that mutations in a residue of Qcr7 predicted to bind PE impaired complex III activity (Fig. 9e). To our knowledge, this is the first molecular evidence demonstrating the functional importance of a specific interaction of PE with complex III, which until now had only been postulated from crystal structures of yeast and human RSCs^31,32^. A second PE is found adjacent to the dimer interface of this complex; as such, its acyl chains are thought to interact with both monomers. However, PE depletion did not disrupt dimer formation (Fig. 5d, e). PE at the dimer interface could instead potentially promote quinol-quinone exchange at the Q_i_ and Q_0_ sites. More broadly, during quinol-quinone exchange, sidechain movement of Cob1 is thought to be necessary to transfer protons from His202 to ubiquinone^42^. If depletion of PE or mutagenesis of residues that interact with this lipid diminish the efficiency of electron transfer between complex III monomers or between complex III and its substrates, this could result in reduced complex III function, potentially as a means to prevent superoxide production^43^. These structural observations will guide future efforts to determine the role(s) of PE as it relates to complex III activity.

## Methods

### Yeast strains and growth conditions

All yeast strains used in this study are listed in Table 1 and were derived from GA74–1A unless otherwise noted. Deletion strains were generated by PCR-mediated gene replacement of the entire reading frame using a selectable marker as indicated in Table 1^33^. Psd1 containing a COOH-terminal 3XFLAG tag subcloned into pRS305 has been described^17,44^. To re-direct Psd1 to the mitochondrial OM, the first 100 amino acids of Psd1, encompassing its mitochondrial targeting sequence and transmembrane domain, were replaced by the equivalent domains (amino acids 1–34) of the single-pass OM resident protein, Tom20. ER-Psd1, which is directed to the secretory pathway, was generated by replacing the first 57 amino acids of Psd1, encompassing its mitochondrial targeting signal (MTS), with the N-terminal signal sequence (amino acids 1–23) of carboxypeptidase Y, as previously described^17^. Additionally, the ER-Psd1 construct contains an NXS *N-*glycosylation signal immediately downstream of the CPY leader sequence to track its topology. Details of the primers used in this study are listed in Supplementary Table 1. The IM-Psd1, OM-Psd1, and ER-Psd1 constructs, which all contained the C-terminal 3XFLAG tag, were subcloned into the pRS305 plasmid, linearized, and integrated into the *LEU2* locus in the *psd1*Δ*psd2*Δ background. Clones were selected on synthetic dropout medium (0.17% (w/v) yeast nitrogen base, 0.5% (w/v) ammonium sulfate, 0.2% (w/v) dropout mixture synthetic-leu, 2% (w/v) dextrose) and verified by immunoblot.

**Table 1:** Yeast strains used in this work. The names, genotypes, and sources of the yeast strains used in the present study

Strain	Genotype	Source
GA74-1A	*MAT*a, *his3-11,15, leu2, ura3, trp1, ade8 [rho* *+* *, mit* *+* *]*	Carla Koehler
*psd1Δ*	*MAT*a, *psd1Δ::HIS3MX6, leu2, ura3, trp1, ade8 [rho* *+* *, mit* *+* *]*	ref. ^17^
*psd2Δ*	*MAT*a, *psd2Δ::HIS3MX6, leu2, ura3, trp1, ade8 [rho* *+* *, mit* *+* *]*	ref. ^17^
*psd1Δpsd2Δ*	*MAT*a, *psd2Δ::HIS3MX6, leu2, ura3, psd1Δ::TRP1, ade8 [rho* *+* *, mit* *+* *]*	ref. ^17^
*crd1Δ*	*MAT*a, *his3-11,15, leu2, ura3, crd1Δ::TRP1, ade8 [rho* *+* *, mit* *+* *]*	ref. ^48^
ρ^0^	*MAT*a, *his3-11,15, leu2, ura3, trp1, ade8 [rho-, mit* *+* *]*	This study
*cho1Δ*	*MATa, cho1Δ::HIS3MX6, leu2, ura3, trp1, ade8 [rho* *+* *, mit* *+* *]*	ref. ^17^
*psd1Δpsd2Δ::IM-Psd1*	*MAT*a, *psd2Δ::HIS3MX6, Psd3XFLAG::LEU2, ura3, psd1Δ::TRP1, ade8 [rho* *+* *, mit* *+* *]*	ref. ^17^
*psd1Δpsd2Δ::OM-Psd1*	*MAT*a, *psd2Δ::HIS3MX6, Tom20-Psd3XFLAG::LEU2, ura3, psd1Δ::TRP1, ade8 [rho* *+* *, mit* *+* *]*	This study
*psd1Δpsd2Δ::ER-Psd1*	*MAT*a, psd2Δ::HIS3MX6, CPY*mPsd3XFLAG::LEU2, ura3, psd1Δ::TRP1, ade8 [rho *+* *, mit* *+* *]*	Ref. ^17^
*cho1Δpsd1Δpsd2Δ*	MATa, psd2Δ::HIS3MX6, leu2, ura3, psd1Δ::TRP1, *cho1 HI-CRISPR ade8 [rho *+* *, mit* *+* *]*	This study
*Qcr7p E82R*	*MATa, his3-11, 15, leu2, ura3, trp1, ade8 [rho*^*+*^*, mit*^*+*^] *Qcr7p^E82R^ *HI-CRISPR*	This study
*Qcr7p E82D*	*MATa, his3-11, 15, leu2, ura3, trp1, ade8 [rho*^*+*^*, mit*^*+*^] *Qcr7p^E82D^ *HI-CRISPR*	This study
W303	*MAT*a, *his3-11, ura3-1,15, trp-1-1, ade2-1, can1-100 [rho* *+* *, mit* *+* *]*	Cathy Clarke
*psd1Δ* W303	*MAT*a, *psd1Δ::HIS3MX6, ura3-1,15, trp1-1, ade2-1, can1-100*	This study
D273-10B	*MATa [rho* *+* *, mit* *+* *]*	Carla Koehler
*psd1Δ D273-10B*	*MAT*a, *psd1Δ::KANMX6, [rho* *+* *, mit* *+* *]*	This study
*BY4741*	*MAT*a, *his3-1*, *leu2-0*, *met15-0*, *ura3-0 [rho* *+* *, mit* *+* *]*	Euroscarf
*psd1Δ BY4741*	*MAT*a, *psd1Δ::KANMX4,his3-1*, *leu2-0*, *met15-0*, *ura3-0 [rho* *+* *, mit* *+* *]*	Euroscarf
*abf2Δ*	*MAT*a, *his3-11,15, leu2, ura3, abf2Δ::TRP1, ade8 [rho* *+* *, mit* *+* *]*	This study

For strains that were genetically modified by homology-integrated clustered regulatory interspaced short palindromic repeats (CRISPR)-Cas (HI-CRISPR)^45^, CRISPR-Cas9 gene blocks were designed to target *CHO1* or *QCR7* and cloned into the pCRCT plasmid^45^, as previously described^44^. The spacer sequences (including the CRISPR-Cas9 target (20-bp) and the homology repair template (50-bp) homology arms on both sides (total 100-bp) flanking the Cas9 recognition sequence) were ordered as gBlocks (Integrated DNA Technologies) The sequence of all gBlocks used in this study are provided in Supplementary Table 2. Specifically, *CHO1* knockout was achieved by incorporating an 8-bp deletion within the homology repair template to induce a frameshift mutation by removal of nucleotides 38–45 downstream of the start ATG sequence of the *CHO1* open reading frame (ORF). The CRISPR construct was designed to target the protospacer adjacent motif (PAM) sequence encoded by nucleotides 40–42 on the reverse strand of the *CHO1* ORF. Single point mutations were introduced into the *QCR7* ORF at positions 244–246 downstream of the ATG start site to mutate Glu82 (GAG) to Asp82 (GAC) or Arg82 (AGA) through the design of homology repair templates encoding these mutations. Specificity of gene block integration into this locus was accomplished by targeting the PAM sequence encoded by nucleotides 209–211 on the reverse strand of the *QCR7* ORF. Both *QCR7* gene blocks encoded for silent alanine mutations at position 208–210 to mutate the PAM sequence and prevent re-cleavage by Cas9 after homology-directed repair, and position 214–216 to prevent hairpin formation of gBlocks. Additionally, the homology arms adjacent to the PAM sequence were extended to 125-bp on each side for these constructs. Integration of gene blocks encoding for these mutations were verified by sequencing of yeast genomic DNA using primers specific for *QCR7*. A 10XHis tag was introduced into the C-terminus of Cox8 expressed in WT, *psd1*Δ, *psd2*Δ, *psd1*Δ*psd2*Δ, IM-Psd1, OM-Psd1, and ER-Psd1. The Cox8–10XHis gene block was designed to target a PAM sequence on the 3’ untranslated region (UTR) downstream of the *COX8* ORF. Specifically, this was located on the reverse strand 242–244 nucleotides downstream of the *COX8* ATG start site. The homology arms of the Cox8-10XHis gene block included a 76-bp extension upstream of the original TAA stop sequence, which was replaced by the inclusion of a 3X alanine (GCT) linker, 10X His (CA(T/C)) sequence, followed by a new TAA stop sequence. Silent mutations were introduced at nucleotide positions 242–244 to recode the PAM sequence to prevent re-cleavage by Cas9 after homology-directed repair, and at position 246 to prevent hairpin formation of the gBlocks. These mutations were followed by an 83-bp extension homology arm. Expression of Cox8-His was verified in yeast and mitochondrial lysates by immunoblot using a 6 × -His monoclonal antibody (His.H8, Thermofisher).

Yeast were grown on YPD (1% (w/v) yeast extract, 2% (w/v) tryptone, 2% (w/v) dextose) plates. To assess the function of the assorted re-directed Psd1 constructs, overnight cultures grown in synthetic complete dextrose (SCD; 0.17% (w/v) yeast nitrogen base, 0.5% (w/v) ammonium sulfate, 0.2% (w/v) complete amino acid mixture, 2% (w/v) dextrose) supplemented with 2 mM ethanolamine hydrochloride were spotted on SCD plates in the absence or presence of 2 mM ethanolamine hydrochloride, or spotted onto rich lactate (1% (w/v) yeast extract, 2% (w/v) tryptone, 0.05% (w/v) dextrose, 2% (v/v) lactic acid, 3.4 mM CaCl_2_-2H_2_O, 8.5 mM NaCl, 2.95 mM MgCl_2_-6H_2_O, 7.35 mM KH_2_PO_4_, 18.7 mM NH_4_Cl, pH 5.5), or synthetic complete ethanol glycerol (SCEG; 0.17% (w/v) yeast nitrogen base, 0.5% (w/v) ammonium sulfate, 0.2% (w/v) complete amino acid mixture, 1% (v/v) ethanol, 3% (v/v) glycerol) or synthetic complete lactate (SC-LAC; 0.17% (w/v) yeast nitrogen base, 0.5% (w/v) ammonium sulfate, 0.2% (w/v) complete amino acid mixture, 0.05% (w/v) dextrose, 2% (v/v) lactic acid, 3.4 mM CaCl_2_-2H_2_O, 8.5 mM NaCl, 2.95 mM MgCl_2_-6H_2_O, 7.35 mM KH_2_PO_4_, 18.7 mM NH_4_Cl, pH 5.5) in the absence or presence of 2 mM ethanolamine hydrochloride to test respiratory growth.

For CoQ_6_ supplementation experiments, starter cultures were grown in SCD + 2 mM ethanolamine overnight and diluted to 0.025 OD_600_ in 500 μL of either SCEG, SCEG + 2 mM ethanolamine, SCEG + 2μM CoQ_6_ (Avanti Polar Lipids, Inc), SCEG + 2 μM CoQ_6_ + 2 mM ethanolamine, SCEG + 10μM CoQ_6_, or SCEG + 10μM CoQ_6_ + 2 mM ethanolamine in duplicate in a 48 well plate. OD_600_ measurements were then recorded every 30 min for a period of 48 h at 30 °C using a Tecan Infinite 200 Pro instrument. For experiments designed to test the importance of the pABA pathway for cell growth, synthetic media lacking pABA was utilized for liquid and solid growth of yeast cells. -pABA media consisted of 790 mg/mL CSM Mixture Complete (Formedium LTD) and 6.9 g/L yeast nitrogen base lacking amino acids and *para*-amino benzoic acid (Formedium LTD). For + pABA media, the same mixture was used but included the addition of 100μM *para*-amino benzoic acid (Research Products International, Inc) dissolved in water and sterile filtered using a 0.20μM filter (Corning, Inc). Individual colonies of yeast were used to inoculate starter cultures in -pABA medium containing 2% (w/v) glucose and 2 mM ethanolamine. Solid growth of yeast on agar plates was evaluated in -/ + pABA medium containing 1% (v/v) ethanol and 3% (v/v) glycerol −/+ 2 mM ethanolamine.

### Preparation of yeast cell extracts

Overnight 2 mL cultures of yeast were grown in YPD, rich lactate, or SCD with or without 2 mM ethanolamine as indicated in figure legends. 1 OD_600_ of exponentially growing cells was collected in a 1.5 mL microcentrifuge tube by centrifugation for 10 min at 845 *xg* at room temperature. The supernatant was aspirated and the cell pellet resuspended in 1 mL of water. A freshly made NaOH/β-mercaptoethanol solution consisting of 1 mL 2 M NaOH and 80 µL of β-mercaptoethanol was prepared, and 150 µL of this solution added to the cell resuspension which was mixed by inversion. Tubes were incubated for 10 min on ice and mixed by inversion every 2 min. 75 µL of 100% (w/v) tricholoroacetic acid (TCA) was added to precipitate proteins in each sample tube and incubated on ice for 10 min with mixing by inversion every 2 min. Samples were centrifuged for 2 min at 21,000 *x g* at 4 °C. The supernatant was aspirated and pellets washed using 500 µL of 100% cold acetone. Samples were centrifuged for 2 min at 21,000 *x g* at 4 °C and the acetone decanted. The pellet was briefly air-dried and then resuspended with 30 µL of 0.1 M NaOH. After dissolution of the pellets, an equal volume of 2X reducing sample buffer was added and samples boiled for 5 min at 95 °C. 5 µL of yeast extracts were evaluated by SDS-PAGE and immunoblotting.

### Immunoblotting

Protein samples in reducing sample buffer were resolved on either 12% or custom-made 10–16% gradient SDS-PAGE gels at ~5.8 mAmp/hr. Proteins were transferred to nitrocellulose membranes (Bio-Rad Laboratories Inc, 0.45 µM Catalog No. 1620115) at 30 Volts overnight (16 h) at room temperature. The quality of the transfer was check by Ponceau S staining for 15 s, and membrane strips were cut to probe for proteins at their verified molecular weight migration. As described^46,47^, membranes were blocked with 5% (w/v) milk (Giant, Ellicott City, MD), 0.05% (v/v) Tween-20/1XPBS for 1 h and then incubated with primary antibody with rocking for 1 h at room temperature. Following three successive 10 min washes with PBST (PBS with 0.2% Tween-20), HRP- or IRDye 800CW-conjugated secondary antibodies were added for 45 min, the membranes were washed three times for 10 min with PBST and twice for 10 min with PBS. When using HRP-conjugated secondary antibodies, immunoreactive bands were visualized using the SuperSignal West Pico chemiluminescent substrate from Pierce and imaged using a Fluorchem Q (ProteinSimple). Immunoblots using IR 800 CW secondary antibodies were imaged using an Odyssey CLx Imaging System. To reprobe a blot, membranes were incubated with stripping buffer (100 mM *β*-mercaptoethanol (*β*-ME), 2% (w/v) SDS, 62.5 mM Tris, pH 6.7) shaking at 37^o^C for at least 1 h and then washed 5 times, 10 min/wash, with PBST prior to blocking the membrane again.

### Mitochondrial isolation and fractionation

Crude mitochondria were isolated as described^47^. Yeast were maintained on rich lactate plates and grown in rich lactate media to prevent the loss of mitochondrial DNA. To improve growth on rich lactate, *psd1*Δ*psd2*Δ yeast were grown in the presence of 2 mM choline prior to harvesting mitochondria (except where indicated in Fig. 7a, b and in Fig. 8). For mitochondrial isolation, starter cultures of 100 mL (or 150 mL for *psd1*Δ*psd2*Δ) were grown at 30 °C for 36–48 h until saturation. 100 OD_600_ of the starter cultures were sterilely added to 2 L flasks containing 950 mL rich lactate and grown for ~18 h (or 24 h for *psd1*Δ*psd2*Δ) at which point the cultures had reached an OD_600_ of ∼2.5–3.5 (two 2 L flasks were grown for each strain). Cells were harvested by centrifugation (5 min at 6,000 *x g* at room temperature) using 1000 mL buckets and the cell pellets resuspended with ~100 mL of water and transferred to pre-weighed 250 mL tubes. Cells were collected at 2000 *x g* for 5 min at room temperature by centrifugation. The supernatant was decanted and the cell pellets weighed (typically yield is ~6 g of yeast/L of culture). Cells were suspended in 50 mL of freshly prepared 0.1 M Tris-SO_4,_ pH 9.4 (1 M Tris with pH adjusted with sulfuric acid) containing 15 mM dithiothreitol, and incubated for 20 min at 30 °C shaking at 220 rpm. Cell pellets were collected by centrifugation at 2000 *x g* for 5 min at room temperature and washed with 40 mL of 1.2 M sorbitol, 20 mM KPi, pH 7.4 buffer. Pellets were collected again at 2000 × *g* for 5 min at room temperature. To convert cells to spheroplasts, yeast pellets were resuspended in 1.2 M sorbitol, 20 mM KPi, pH 7.4, containing 3 mg of Zymolyase 20 T (nacalai tesque, INC.) per gram of yeast, using a final volume of 2 mL per gram of yeast, and the suspensions shaken at 220 rpm for 1 h at 30 °C. Spheroplasting efficiency was monitored visually by checking for osmolysis upon dilution of a small drop of yeast slurry in 10 µL H_2_O. Spheroplasts were collected by centrifugation at 3500 *x g* for 5 min at 4 °C. From this point on, all operations were conducted at 4 °C and/or on ice. Cell pellets were washed twice with cold (4 °C chilled) 1.2 M sorbitol, 20 mM KPi, pH 7.4 buffer and collected by centrifugation at 3500 × *g* for 5 min at 4 °C. For homogenization, spheroplasts were resuspended in 50 mL of 0.6 M sorbitol, 20 mM KOH-MES, pH 6 (BB6.0 buffer) containing 1 mM phenylmethylsulfonyl fluoride (PMSF), transferred to a tight-fitting (type A) glass dounce homogenizer (pre-chilled on ice), and homogenized with 15 strokes. For cell fractionation studies, 200 µL of this homogenate was collected and placed on ice to quantitate and analyze as the starting material (SM, Supplementary Fig. S1). The combined homogenate was centrifuged for 5 min at 1700 *x g* at 4 °C, and the supernatants collected. The residual pellets were re-homogenized in BB6.0 + 1 mM PMSF by 15 strokes in the same glass dounce. Following centrifugation of the homogenate at 1700 *x g* at 4 °C, the supernatants from both homogenizations were combined and then centrifuged for 10 min at 13,500 *x g*. The pellets following this centrifugation contain crude mitochondria. The resulting supernatants (S13) were either processed for additional cell density-based cell fractionation steps (described below) or discarded. The mitochondrial pellets were washed with 35 mL of BB6.0 buffer without PMSF. Mitochondria were resuspended 2 times using a pre-chilled teflon dounce and collected in the supernatant following a 1700 *x g* 5 min spin at 4 °C. This supernatant (avoiding dissolution of the pellet) was transferred to a fresh 50 mL tube and centrifuged at 13,500 × *g* for 10 min at 4 °C. The supernatant was aspirated, and the mitochondrial pellet washed once with ~30 mL of 0.6 M sorbitol, 20 mM K^+^HEPES, pH 7.4 (BB7.4) buffer. Mitochondria were resuspended 2 times using a pre-chilled teflon dounce and transferred to a clean 50 mL tube for collection of the final crude mitochondrial pellet (P13) at 13,500 *x g* for 10 min at 4 °C. The supernatant was aspirated and the mitochondrial pellets resuspended with residual BB7.4. Protein concentration was determined using the Pierce BCA Protein Assay Kit (ThermoFisher Scientific, Catalog No. 23225). Aliquots of mitochondria (1 mg at ~25 mg.mL) were snap frozen in liquid nitrogen and stored at −80 °C.

To collect additional subcellular fractions, 35 mL of the S13 supernatant (see Methods section above for isolation of crude mitochondria), was transferred to a 50 mL tube and centrifuged at 21,500 × *g* for 15 min at 4 °C. The resulting supernatant was transferred to a fresh 50 mL tube and spun at 40,000 × *g* for 30 min at 4 °C. The resulting supernatant (S40), which contains the cytosol, was transferred to a 50 mL falcon tube. The 40,000 *x g* pellet (P40) was resuspended in residual buffer and transferred to a 1.5 mL microcentrifuge tube. The BCA assay was used to determine the protein concentration of each collected fraction (starting material, P13, P40, and S40). Aliquots of cell fractionation samples were snap frozen in liquid nitrogen and stored at -80 °C.

For sucrose gradient purified mitochondria^17^, 4 mg/ml crude mitochondria in SEM buffer (250 mm sucrose, 1 mm EDTA, 10 mm MOPS, pH 7.2) were homogenized by 10 strokes using a teflon dounce. The resultant crude mitochondrial suspension was layered onto a sucrose step gradient (1.5 ml of 15% sucrose, 1.5 ml of 23% sucrose, 4 ml of 32% sucrose, and 1.5 ml of 60% sucrose in EM buffer (1 mm EDTA, 10 mm MOPS, pH 7.2) and centrifuged at 134,000 × *g* for 1 h at 4 °C. Mitochondria were recovered from the 32–60% sucrose interface, resuspended in SEM buffer to dilute the sucrose concentration, and re-collected by centrifugation at 13,500 × *g* for 10 min at 4 °C. Mitochondria were washed in ice-cold BB7.4, the yield determined using the BCA assay, and aliquots snap frozen in liquid nitrogen and stored at −80 °C.

Submitochondrial localization of the Psd1 constructs utilized an established protease accessibility assay^47^. This protocol involves the generation of 3 different treatment groups (intact mitochondria, OM-disrupted mitoplasts, and detergent-solubilized mitochondria), each analyzed in the presence or absence of the protease, Proteinase K. For intact mitochondria, two microcentrifuge tubes containing 150 µg of mitochondrial proteins were set aside on ice. For OM-disrupted/detergent solubilized samples, 600 µg of mitochondria was collected by centrifugation at 8,000 *x g* for 5 min at 4 °C. The mitochondrial pellet was resuspended in 200 µL of BB7.4 and 50 µL of this mixture aliquoted into four tubes. To rupture the OM, 19x volumes of 20 mM K^+^HEPES, pH 7.4, lacking or containing 100 µg/mL of Proteinase K, was added to mitochondria. The 20 mM K^+^HEPES, pH 7.4 (without or with Proteinase K) was spiked with deoxycholate (final 0.5% (w/v)) for the detergent-solubilized samples. In parallel, the intact mitochondrial samples were resuspended in 1 mL BB7.4 buffer lacking or containing 100 µg/mL of Proteinase K. Following a brief 10 sec vortex (medium setting of 7 using a Vortex-Genie), the samples were incubated on ice for 30 min. To inhibit Proteinase K, PMSF was added (5 mM final) to each sample, which were then centrifuged at 21,000 × *g* for 10 min at 4 °C. For intact mitochondria and OM-ruptured mitoplasts, the supernatant was aspirated and Proteinase K completely inactivated by resuspension in 180 µL of BB7.4 containing 1 mM PMSF. This resuspension was transferred to a new tube containing 20 µL of 100% (w/v) TCA. Following a brief 5 min incubation at 60^o^C, samples were kept on ice for another 5 min. For detergent-solubilized mitochondria, the supernatants were transferred to tubes containing 0.2 mL of 100% (w/v) TCA and placed on ice for 1 h. Following these treatments and incubations, every sample was centrifuged at 21,000 *x g* for 10 min at 4 °C, the supernatants aspirated, and pellets washed with 0.5 mL of cold acetone. Following a 10 min 21,000 × *g* centrifugation at 4 °C, the acetone was decanted and the pellets incubated with 30 µL of 0.1 M NaOH for 30 min at room temperature. An equal volume of 2X reducing sample buffer was added to each sample which were then boiled for 5 min at 95 °C. 12 µL of each sample was evaluated by SDS-PAGE and western blotting.

### Phospholipid analyses

1.2 mg of sucrose purified mitochondria were transferred to 5 mL borosilicate tubes containing 1.5 mL of 2:1 chloroform:methanol. After vigorous vortexing at room temperature for 30 min, 0.3 mL of 0.9% (w/v) NaCl was added to each tube which were vortexed for another 1 min. Tubes were centrifuged at 1000 rpm for 5 min at room temperature. The aqueous phase was aspirated and the organic phase washed with 0.25 mL of 1:1 methanol:water. Tubes were vortexed for 30 s, centrifuged for 5 min at 1000 rpm at room temperate and the lower organic phase transferred to new 5 mL borosilicate tubes using a clean glass pasteur pipette. Lipid samples were dried down under a stream of liquid nitrogen. For phosphate quantitation, mitochondrial lipids were resuspended in 50 µL of chloroform, and 1 and 2 µL transferred to clean, unused glass tubes; the rest of the mitochondrial lipids were re-evaporated under a stream of liquid nitrogen. The 1 µL and 2 µL lipid samples were evaporated and 150 µL of 70% (w/v) perchloric acid added to each sample. A phosphate standard curve containing 5, 10, 15, 20, and 25 nmol of KPi was treated in parallel. Samples were vortexed, incubated at 180 °C for 40 min, cooled and 500 µL of water added to each tube and vortexed. 200 µL of 1.25% (w/v) ammonia molybdate was added to each sample and vortexed. Next, 200 µL of 5% (w/v) ascorbic acid was added to each sample and vortexed. All samples were incubated at 100 °C for 5 min and their absorption determined at 797 nM.

For ^32^P-based phospholipid analyses, starter cultures were diluted to an OD_600_ = 0.4 in 2 ml of rich lactate medium supplemented with 10 μCi/mL ^32^P_i_ and grown shaking at 240 rpm for ∼24 h at 30 °C. Where indicated, cultures additionally contained 2 mM choline or 2 mM ethanolamine. Yeast were recovered by centrifugation (1690 *x g* for 5 min), washed with H_2_O, re-centrifuged, and the yeast pellets resuspended in 0.3 mL MTE buffer (0.65 M mannitol, 20 mM Tris, pH 8.0, and 1 mM EDTA) supplemented with 1 mM PMSF, 10 μM leupeptin, and 2 μM pepstatin A and transferred to a microcentrifuge tube containing 0.1 mL glass beads. Each tube was sealed with parafilm and the yeast were disintegrated by vortexing on high for ∼30 min at 4 °C. The glass beads and unbroken yeast were discarded following a low-speed 4 °C spin at 250 × *g*. A crude mitochondrial fraction was collected from the remaining supernatant by centrifugation for 5 min at 13,000 × *g* at 4 °C. Phospholipids from equal amounts of labeled crude mitochondria, as determined by liquid scintillation, were extracted as described in previous section using sucrose purified mitochondria.

For evaluation by thin layer chromatography, dried lipid samples were resuspended in 13 µL of chloroform and loaded on Silica Gel GHLF (Analtech) or SILGUR-25 (Machery-Nagel) TLC plates that were pretreated with 1.8% (w/v) boric acid in 100% ethanol and activated at 95 °C for at least 30 min. The loaded plates were resolved using a solvent system containing chloroform/ethanol/H_2_O/triethylamine (30:35:7:35). The plate was air-dried for 30 min and either sprayed manually using a TLC sprayer (CAMAG) and the molybdenum spray reagent (Sigma) or developed using a K-screen and FX-Imager (Bio-Rad Laboratories).

### Electron microscopy

Cells were grown in rich lactate medium and harvested at mid-log phase by centrifugation. Cells were fixed in 3% glutaraldehyde contained in 0.1 M sodium cacodylate, pH 7.4, 5 mM CaCl_2_, 5 mM MgCl_2_, and 2.5% (w/v) sucrose for 1 h at room temperature with gentle agitation, spheroplasted, embedded in 2% ultra-low temperature agarose (prepared in water), cooled, and subsequently cut into small pieces (∼1 mm^3^) as previously described^33^. The cells were then post-fixed in 1% OsO_4_, 1% potassium ferrocyanide contained in 0.1 M sodium cacodylate, 5 mM CaCl_2_, pH 7.4, for 30 min at room temperature. The blocks were washed thoroughly four times with double distilled H_2_O, 10 min total, transferred to 1% thiocarbohydrazide at room temperature for 3 min, washed in double distilled H_2_O (four times, 1 min each), and transferred to 1% OsO_4_, 1% potassium ferrocyanide in 0.1 M sodium cacodylate, pH 7.4, for an additional 3 min at room temperature. The cells were washed four times with double distilled H_2_O (15 min total), stained en bloc in Kellenberger’s uranyl acetate for 2 h to overnight, dehydrated through a graded series of ethanol, and subsequently embedded in Spurr resin. Sections were cut on a Reichert Ultracut T ultramicrotome, post-stained with uranyl acetate and lead citrate, and observed on an FEI Tecnai 12 transmission electron microscope at 100 kV. Images were recorded with a Soft Imaging System Megaview III digital camera, and figures were assembled in Adobe Photoshop with only linear adjustments in contrast and brightness.

### mtDNA quantitation

Yeast cells were grown for 2 days in rich lactate and the collected cell pellets were vortexed at level 10 for 3 min with 200μL breaking buffer (2% (v/v) Triton X-100, 1% (v/v) SDS, 100 mM NaCl, 10 mM Tris pH8.0, 1 mM EDTA pH 8.0), 0.3 g glass beads, and 200μL phenol/chloroform/isoamyl alcohol at room temperature. The solution was neutralized with the addition of 200 μL of Tris-EDTA (TE) buffer pH 8.0 and phases were separated by centrifugation at 21,000 × *g* for 5 min. The aqueous phase was collected and DNA was precipitated by the addition of 100% ethanol and collected in the pellet after centrifugation at 21,000 × *g* for 3 min. The pellets were resuspended in 400 μL TE buffer, pH 8.0 and RNA was digested with the addition of 3 μL of 10 mg/mL RNAse A and incubation at 37^o^C for 5 min before addition of 10 µL of 4 M Ammonium acetate and 1 mL 100% ethanol. DNA pellets were recovered by centrifugation at 21,000 × *g* for 3 min, dried, and resuspended in 30 µL TE buffer pH 8.0. DNA was stored at −80^o^C until ready for use, quantitated, and a portion diluted to 10 ng/μL to be used as template in the qPCR reaction. The FastStart Universal SYBR Green Master Rox (Roche) was used for qPCR performed according to the manufacturer’s instructions. 50 ng of genomic DNA was used as a template and the following primers were used at 100 nM concentration in a 20 µL reaction: *COX1* forward (5′-CTACAGATACAGCATTTCCAAGA-3′), *COX1* reverse (5′-GTGCCTGAATAGATGATAATGGT-3′), *ACT1* forward (5′-GTATGTGTAAAGCCGGTTTTG-3′), and *ACT1* reverse (5′-CATGATACCTTGGTGTCTTGG-3′). The reactions were performed in technical duplicate with three biological replicates. After completion of thermocycling in a QuantStudio 6 Flex Real-Time PCR System (Thermo Fisher), melting-curve data was collected to verify PCR specificity. The absence of primer dimers and the Ct value difference between the nuclear (*ACT1*) and mitochondrial (*COX1*) target were computed as a measure of the mitochondrial DNA copy number relative to the nuclear genome.

### Mitochondrial respiration measurements

As done previously^48^, mitochondrial oxygen consumption was measured using a Clark-type oxygen electrode in a magnetically stirred, thermostatically controlled 1.5 mL chamber at 25 °C (Oxytherm; Hansatech). 100μg of mitochondria were resuspended in 0.25 M sucrose, 0.25 mg/mL BSA, 20 mM KCl, 20 mM Tris-Cl, 0.5 mM EDTA, 4 mM KH_2_PO_4_, and 3 mM MgCl_2_, pH 7.2. After addition of 1 mM ascorbate + 0.3 mM TMPD, state 2 rate was monitored for approximately 30 sec. State 3 respiration was initiated by addition of 50μM ADP. After state 4 rate was measured, 10μM CCCP was added to induce uncoupled respiration, and the rate was followed for either 2 min or until oxygen level reached zero.

### Complex III and IV activity measurements

Established methods were used to determine complex III and IV activities^33,49^. To measure complex III activity, 25μg of mitochondria solubilized in 0.5% (w/v) *n*-dodecyl-β-D-maltoside were added to reaction buffer (50 mM KP_i_, 2 mM EDTA, pH 7.4) with 0.008% (w/v) horse heart cytochrome *c* and 1 mM KCN. The reaction was started by adding 100μM decylubiquinol, and the reduction of cytochrome *c* followed at 550 nM. Complex IV activity was initiated by adding 5μg of solubilized mitochondria to reaction buffer with 0.008% (w/v) ferrocytochrome *c* and measured by recording cytochrome *c* oxidation at 550 nm.

### Blue native-PAGE

Sedimented mitochondria (150ug) were solubilized with 30 μL of 1.5% (w/v) digitonin (Biosynth International, Inc.) in 20 mM HEPES-KOH, pH 7.4, 10% (v/v) glycerol, 50 mM NaCl, 1 mM EDTA, and 2.5 mM MgCl_2_, and containing protease inhibitors (1 mM PMSF, 10 μM leupeptin, 2 μM pepstatin A), for 30 min on ice. Samples were finger-flicked or briefly vortexed on medium every 5 min. Insoluble material was removed by centrifugation for 30 min at 21,000 × *g* at 4 °C. The digitonin extracts (∼150 μg) were transferred to tubes containing 3 μL of 10X loading dye (5% Coomassie brilliant blue G-250 (Serva), 0.5 M 6-aminocaproic acid, 10 mM BisTris/HCl, pH 7.0) and the samples loaded on custom-made 5–12% linear polyacrylamide gradient gels. After running gels at 100 V for ~3 h at 4 °C using a cathode buffer solution containing 50 mM Tricine, 15 mM Bis-Tris/HCl pH7.0, and 0.02% Coomassie blue G-250 and an anode buffer solution containing 50 mM Bis-Tris/HCl, pH 7.0, the cathode solution was replaced with 50 mM Tricine, 15 mM Bis-Tris/HCl pH7.0 (without any Coomassie blue G-250). Gels were run at 100 V overnight, and the voltage increased in the morning to a maximum of 200 V until sample migration was complete. Following overnight (16 h) transfer onto PVDF membranes at 30 V, the lane containing the high molecular weight standards (Sigma catalog # 17-0445-01) was excised with a razor blade, and the remaining portion of the membrane washed with methanol to remove excess Coomassie blue prior to immunoblotting for complexes as described above. To reveal the migration of the high molecular weight standards, a small amount of Ponceau S stain was added and then washed off with methanol until the standards were visually detected.

### Preparation of styrene maleic acid co-polymer (SMA)

Styrene maleic acid co-polymer with ratio of 3:1 was prepared by alkaline hydrolysis of its anhydride precursor (SMA 3000, a gift from Cray Valley, USA) following a published protocol^50^. Briefly, the styrene maleic anhydride co-polymer was dissolved in 1 M NaOH by heating and refluxing of the solution. Once dissolved, the solution was cooled down and the SMA co-polymer was gradually precipitated by reducing the pH to < 5.0 with HCl. The precipitate was washed with water and re-dissolved in 0.6 M NaOH. The precipitation and washing cycle of SMA was repeated before the precipitate was finally re-dissolved in 0.6 M NaOH. The dissolved polymer was lyophilized using a freeze-dryer (Labconco) after adjusting the pH to 8.0 with HCl.

### Negative staining and TEM imaging

A concentration of 5 µl of purified SMALPs was applied to glow-discharged, carbon coated, 400 mesh copper grids (Electron Microscopy Sciences, USA) and stained with 0.5 % uranyl acetate. TEM images of the grids were acquired on a Tecnai 12 G2 Spirit BioTWIN microscope (FEI, USA) operating at 120 kV.

### Purification of complex IV from SMA extracts

For preparative scale affinity purifications, mitochondria (21 mg) from the indicated strains was resuspended in 1 mL BB7.4 (0.6 M sorbitol, 20 mM HEPES-KOH pH 7.4), diluted in 19 mL ice cold 20 mM HEPES-KOH pH 7.4, vortexed for 10 sec, and incubated on ice for 30 min. Mitoplasts with osmotically ruptured OMs were recovered by centrifugation at 8,000 × *g* for 10 min at 4^o^C. Each pellet was resuspended with 2.55 mL of 2% (w/v) SMA extraction buffer (20 mM HEPES-KOH pH 8.0, 200 mM NaCl) by repeat pipetting and rotated for 4 h at 4^o^C. SMA extracts, separated from non-extracted material by centrifugation at 21,000 × *g* for 10 min at 4 °C, were diluted to 20 mL with Buffer B (20 mM HEPES-KOH pH 8.0, 200 mM NaCl) containing 5 mM imidazole and rotated with 0.8 mL Ni-NTA agarose overnight at 4^o^C in a chromatography column. Two sequential 20 mL gravity washes were performed with Buffer B spiked with 1) 10 mM imidazole and 2) 20 mM imidazole. Bound material was eluted using Buffer B containing 60 mM imidazole. A series of 8 elutions were collected per sample and protein-containing eluates, identified using the BioRad protein assay, were combined. The final concentration of all of the eluates from every sample was re-quantified against a BSA standard curve on a single 96 well plate using the same method.

### Lipidomics

Individual samples (0.13–0.15 mg protein) were accurately transferred into disposable glass culture test tubes, and a mixture of lipid internal standards was added prior to conducting lipid extraction for quantification of all reported lipid species. Lipid extraction was performed by using a modified Bligh and Dyer procedure^51^. In brief, 4 mL chloroform/methanol (1/1; v/v) and an appropriate volume of 50 mM lithium chloride solution to bring the aqueous phase to a final volume of 2 mL was added to each sample. Following a brief 20 s vortex, the samples were centrifuged at 2700 r.p.m. for 10 min and the bottom organic layer transferred to a new borosilicate glass tube. The residual top layer was re-extracted with 2 mL chloroform. The combined organic layers were dried under a nitrogen stream with a nitrogen-evaporator. Individual lipid extracts were resuspended into a volume of 1 mL of chloroform/methanol (1:1, v/v) per mg of protein for mass spectrometric analysis. Individual suspensions were flushed with nitrogen, capped, and stored at −20 °C for future analysis.

For shotgun lipidomics, lipid extracts were further diluted to a final concentration of ~500 fmol total lipids/µL, and mass spectrometric analysis was performed on a triple quadrupole mass spectrometer (TSQ Altis, Thermo Fisher Scientific, San Jose, CA) and a Q exactive mass spectrometer (Thermo Fisher Scientific, San Jose, CA) which were both equipped with an automated nanospray device (TriVersa NanoMate, Advion Bioscience Ltd., Ithaca, NY) as previously described^52^. Identification and quantification of lipid molecular species were performed using an automated software program^53,54^. Data processing including ion peak selection, baseline correction, data transfer, peak intensity comparison, ^13^C deisotoping, and quantitation were conducted using a custom programmed Microsoft Excel macro as previously described^54^ after considering the principles of lipidomics^55^.

### Antibodies

Antibodies used in the study are listed in Supplementary Table 3. Most of these antibodies were generated by our laboratory or in the laboratories of J. Schatz (University of Basel, Basel, Switzerland) or C. Koehler (UCLA) and have been described previously^3,47,48,56–67^. Abf2 specific antibodies were raised in rabbits using His_6_Abf2 as antigen. Mature Abf2 (Lys27-stop codon) was cloned downstream of the His_6_ tag provided in the pET28a vector (Novagen), induced in BL21-CodonPlus(DE3)-RIL *Escherichia coli* and affinity purified with Ni^2+^ agarose (Qiagen). Specificity of the generated antisera is documented in Supplementary Fig. 11. Other antibodies used were mouse anti-Sec62 (kind gift of Dr. David Meyers (UCLA)), mouse anti-FLAG (clone M2, catalog number F3165, Sigma), mouse anti-Dpm1 (catalog number 113686, Abcam), rabbit anti-Qcr7^68^, rabbit antisera reactive to Coq1^69^, Coq4^70^, Coq7^71^, or Coq9^72^, rabbit antisera raised against the C-terminus of Cho1^73^, rabbit anti-Kar2^19^ and horseradish peroxidase-conjugated (Thermo Fisher Scientific) or IRDye 800CW (LI-COR) secondary antibodies.

### Miscellaneous

Uncropped and unprocessed scans of all immunoblots are presented in Supplementary Fig. 12. Immunoblots and TLC plates were quantitated by Quantity One Software (Bio-Rad Laboratories). Statistical comparisons (ns, *P* *>* 0.05; 1 symbol *P* *≤* 0.05; 2 symbols *P* *≤* 0.01; 3 symbols *P* *≤* 0.001; 4 symbols *P* *≤* 0.0001) were performed using SigmaPlot 11 software (Systat Software, San Jose, CA) or Prism 7 (GraphPad). In some cases, replicates of samples were loaded on the same SDS-PAGE gel, and thus borders of neighboring samples may be detected on the borders of some immunoblots. All graphs show the mean ± S.E.M.. At least three biological replicates represent each of the experiments performed in this study, unless otherwise indicated.

## Supplementary information

Supplementary Information

## Data Availability

The authors declare that all data supporting the findings of this study are available within the paper and the supplementary information files. Correspondence and material requests should be directed to S.M.C.
