# Supplementary material for: Phosphatidylethanolamine made in the inner mitochondrial membrane is essential for yeast cytochrome bc1 complex function
Source: Nat Commun. 2019 Mar 29;10:1432. doi: 10.1038/s41467-019-09425-1 (PMC6441012; doi:10.1038/s41467-019-09425-1)

Supplemental Information

"Phosphatidylethanolamine made in the inner mitochondrial membrane is essential for yeast cytochrome *bc*$_1$ complex function"

Calzada et al

Supplementary Table 1 – Primers used in this work

Primers	Use/Description	Sequence
COX1 forward	qPCR analysis of *COX1*	5′- CTACAGATACAGCATTTCCAAGA-3
COX1 reverse	qPCR analysis of *COX1*	5′- GTGCCTGAATAGATGATAATGGT-3′
ACT1 forward	qPCR analysis of *ACT1*	5′- GTATGTGTAAAGCCGGTTTTG-3′
ACT1 reverse	qPCR analysis of *ACT1*	5′- CATGATACCTTGGTGTCTTGG -3′
QCR7 forward	Upstream primer for sequencing *QCR7* for CRISPR-mediated changes.	5′- TACAAGAAGTTAGGGCTCAAATT-3′
QCR7 reverse	Downstream primer for sequencing *QCR7* for CRISPR-mediated changes.	5′- GGAAGAAATACTACGAGGAGAAC-3′
QCR7 E82R forward	Used to screen for *QCR7* E82R mutants.	5′- CTCATCAAACCAGATTGACTCA-3′
QCR7 E82D reverse	Used to screen *QCR7* E82D mutants.	5′- CATCAAACCGACTTGACTCA-3′
3’ pRS425	Primer a for Tom20-based targeting of Psd1 to OM.	5’- TGCTTCCGGCTCCTATGTTG-3'
b 20Psd1	Primer b for Tom20-based targeting of Psd1 to OM.	5’-ACTGGGACATGCTGGCTTTGCTTTTCCTTC-3’
c 20Psd1	Primer c for Tom20-based targeting of Psd1 to OM.	5’- CAAAGCCAGCATGTCCCAGTCGAACCCTATC- 3’
d 20Psd1	Primer d for Tom20-based targeting of Psd1 to OM.	5’- CCTCCTCTGTTCTTCTTTGATAGTCAAAGTAG- 3’
e 20Psd1	Primer e for Tom20-based targeting of Psd1 to OM.	5’- TCAAAGAAGAACAGAGGAGGATGCTACAGAG- 3’
Psd1.2	Primer f for Tom20-based targeting of Psd1 to OM.	5’- GTCTTCGCCTTGTGCTACTG- 3’
5' Abf2 28a NdeI	5' primer for subcloning Abf2 into pET28a	5'-GGAATTCCATATGAAGGCTTCCAAGAGAACGCAGC-3'
3' Abf2 28a HindIII	3' primer for subcloning Abf2 into pET28a	5'-CCCAAGCTTCTAGTTGAGAGGGTAGCGAG- 3'
5 abf2 delete	5' primer for PCR based disruption of *ABF2*; Hybridizes 50 bp immediately upstream of start site.	5'- AACAAGTAAACAGATTAACAAAGAAGCCAATCAATTA CAACAACAAATAACGGATCCCCGGGTTAATTAA- 3'
3 Abf2 delete	3' primer for PCR based disruption of *ABF2*; Hybridizes 50 bp immediately downstream of stop codon.	5'-TAGGAACGGAAAGAATAAAGGCATAAAAAACATTGTG AGAGTACCGCGGTATAGGCCACTAGTGGATCTG- 3'
5 Abf2 genomic	Used to screen for *ABF2* deletion yeast strains.	5'-TTACGAGCCACAGACTTTCC-3'
Trp1 Reverse, SacII	Used for PCR screens for Trp1-based PCR disruptions.	5'-TCCCCGCGGACCTGTCCCACCTGCTTCTG- 3'
3 Abf2 genomic	Used to screen for *ABF2* deletion yeast strains.	5'-AACACTACACACTTGCTTGG
Trp1 Forward	Used for PCR screens for Trp1-based PCR disruptions.	5'-TGAGTCGTGGCAAGAATACC-3'

Supplementary Table 2 – Geneblocks used in this work

gBlocks	Sequence
CHO1 deletion	5'CTTTGGTCTCACCAAAACACGGACACAGACGTTATCGTAAATGAACACAGAGACGAAAATGACGGGTAATGATGTTGGTGGCACATTAAGCAGAAGGGCCTCAAGTATATTTTCTATATGTGCCACCAACTTCATCTGGTTTTAGAGAGAGACCTTTC-3'
QCR7 E82R mutation	5'CTTTGGTCTCACCAAAACTACAAGAAGTTAGGGCTCAAATTTGACGACTTAATTGCAGAGGAAAATCCCATCATGCAGACCGCTTTAAGAAGACTCCCTGAAGATGAATCTTATGCAAGAGCCTATAGAATAATCAGGGCTCATCAAACCAGATTGACTCATCATTTACTGCCAAGAAACGAATGGATCAAAGCCCAAGAGGATGTTCCTTACCTGTTGCCATACATATTAGAAGCTGAAGCTGCAGCTAAGGAGAAGGACGAGTTAGCCTGATTATTCTATATGCTCGTTTTAGAGAGAGACCTTTC-3'
QCR7 E82D mutation	5'CTTTGGTCTCACCAAAACTACAAGAAGTTAGGGCTCAAATTTGACGACTTAATTGCAGAGGAAAATCCCATCATGCAGACCGCTTTAAGAAGACTCCCTGAAGATGAATCTTATGCAAGAGCCTATAGAATAATCAGGGCTCATCAAACCGACTTGACTCATCATTTACTGCCAAGAAACGAATGGATCAAAGCCCAAGAGGATGTTCCTTACCTGTTGCCATACATATTAGAAGCTGAAGCTGCAGCTAAGGAGAAGGACGAGTTAGCCTGATTATTCTATATGCTCGTTTTAGAGAGAGACCTTTC-3'
QCR7 deletion	5'CTTTGGTCTCACCAAAACAGTCTTTTACGTCTATTGCGAGAATTGGTGACTATATTTTGAAGTCACCCCAAGTAATGTGTTCCAGTTGCCAATCAGTTCATTAACCTCGCAGGTTACACTGGAACACATAACTTGGAGGTTTTAGAGAGAGACCTTTC-3'
COX8-10xHis insertion	5'CTTTGGTCTCACCAAAACTTTCGGGTTCTTCGCTATTGGATTTGCTGTTCCATTTGTTGCCTGCTATGTTCAATTGAAAAAGTCAGGTGCTTTTGCTGCTGCACATCATCACCATCACCACCATCATCACCATTAAAACAGGCGCATAAGTTTGAAGGATAGATGTGTGTACATAGCGTGCTTGGTTGAGACGTTTTAGAGTGTGTTCTTTGCTATTCCTAGGTGCTCTATCCTTCAAACTTATGGGTTTTAGAGAGAGACCTTTC-3'

Supplementary Table 3 – Antibodies used in this work

Antibody	Source[Ref]; Identifier; Dilution Used
Rabbit polyclonal anti-yeast Psd1	Claypool Lab [1]; 4077, 4078; Immunoblot 1:1,000
Mouse monoclonal anti-FLAG	Sigma clone M2; Cat #F3165, Immunoblot 1:5,000
Rabbit polyclonal anti-yeast Tom70	Claypool Lab [2]; 7306; Immunoblot 1:10,000
Rabbit polyclonal anti-yeast Tim54	Claypool Lab [3]; 7303; Immunoblot 1:2,000
Rabbit polyclonal anti-yeast Abf2	Claypool Lab (This study); 5477; Immunoblot 1:8,000
Mouse monoclonal anti-yeast Dpm1	LifeTechnologies; Cat. #5C5A7; Immunoblot 1:1,000
Rabbit polyclonal anti-yeast Cor2	Koehler Lab [4]; CC2-T; Immunoblot 1:5,000
Rabbit polyclonal anti-yeast Rip1	Claypool Lab [2]; MGB71.T; Immunoblot 1:1,000, 1D blue native PAGE 1:2,000
Rabbit polyclonal anti-yeast Qcr6	Claypool Lab [2]; MGB73.2; Immunoblot 1:5,000
Rabbit polyclonal anti-yeast Qcr7	Martin Ott [5]; Immunoblot 1:2,000
Rabbit polyclonal anti-yeast Cox1	G. Schatz [6]; DD2-4; Immunoblot 1:4,000
Mouse monoclonal anti-yeast Cox2	Abcam; [4B12A5] ab110271; Immunoblot 1:2,000
Mouse monoclonal anti-yeast Cox3	Abcam; DA5BC4 Cat #MS406; Immunoblot 1:2,000
Rabbit polyclonal anti-yeast Cox4	Claypool Lab [2]; MGB65-T ; Immunoblot 1:5000; 1D blue native PAGE (1:8,000)
Rabbit polyclonal anti-yeast Cox4 and Cox5	G. Schatz [7]; 203.2; Immunoblot 1:2000
Rabbit polyclonal anti-yeast Atp1 and Atp2	G. Schatz [8]; MP3-T; Immunoblot 1:10,000
Rabbit polyclonal anti-yeast Coq1	Cathy Clarke [9]; Immunoblot 1:10,000
Rabbit polyclonal anti-yeast Coq4	Cathy Clarke [10]; Immunoblot 1:2,000
Rabbit polyclonal anti-yeast Coq7	Cathy Clarke [11]; Immunoblot 1:1,000
Rabbit polyclonal anti-yeast Coq9	Cathy Clarke [12]; Immunoblot 1:1,000
Rabbit polyclonal anti-yeast Por1	G. Schatz [13]; 425; Immunoblot 1:10,000
Rabbit polyclonal anti-yeast Pic1	Claypool Lab [14]; 3676.1; Immunoblot 1:5,000
Rabbit polyclonal anti-yeast Kgd1	G. Schatz [15]; 453.3; Immunoblot 1:2,000
Rabbit polyclonal anti-yeast Ccp1	G. Schatz [16]; SS20-T; Immunoblot 1:8,000
Rabbit polyclonal anti-yeast Cytb2	G. Schatz [13]; 531-T; Immunoblot 1:2,000
Mouse monoclonal anti-yeast Aac2	Panneels V *et al* [17]; 6H8; Immunoblot 1:2,000
Rabbit polyclonal anti-yeast OM45	G. Schatz [18]; SS89-6; Immunoblot 1:10,000
Rabbit polyclonal anti-yeast Cho1	George Carman [19]; Immunoblot 1;5,000
Rabbit polyclonal anti-yeast Hsp70	Koehler Lab [20]; SH1-T; Immunoblot 1:10,000
Mouse monoclonal anti-yeast Sec62	David Meyer [20]; Purple top; Immunoblot 1:1,000
Rabbit polyclonal anti-yeast Kar2	Susan Michaelis [21]; Immunoblot 1:20,000
Rabbit polyclonal anti-yeast Hxk1	Susan Michaelis [21]; Immunoblot 1:200,000
Mouse monoclonal anti-6xHis	Invitrogen; His.H8 Cat #MA1-21315; 1:3,000

Supplementary References

1 Tamura, Y. *et al.* Phosphatidylethanolamine biosynthesis in mitochondria: phosphatidylserine (PS) trafficking is independent of a PS decarboxylase and intermembrane space proteins UPS1P and UPS2P. *J Biol Chem* **287**, 43961-43971 (2012).
2 Baile, M. G., Whited, K. & Claypool, S. M. Deacylation on the matrix side of the mitochondrial inner membrane regulates cardiolipin remodeling. *Mol Biol Cell* **24**, 2008-2020 (2013).
3 Hwang, D. K., Claypool, S. M., Leuenberger, D., Tienson, H. L. & Koehler, C. M. Tim54p connects inner membrane assembly and proteolytic pathways in the mitochondrion. *J Cell Biol* **178**, 1161-1175 (2007).
4 Claypool, S. M., Oktay, Y., Boontheung, P., Loo, J. A. & Koehler, C. M. Cardiolipin defines the interactome of the major ADP/ATP carrier protein of the mitochondrial inner membrane. *J Cell Biol* **182**, 937-950 (2008).
5 Hildenbeutel, M. *et al.* Assembly factors monitor sequential hemylation of cytochrome b to regulate mitochondrial translation. *J Cell Biol* **205**, 511-524 (2014).
6 Dowhan, W., Bibus, C. R. & Schatz, G. The cytoplasmically-made subunit IV is necessary for assembly of cytochrome c oxidase in yeast. *EMBO J* **4**, 179-184 (1985).
7 Poyton, R. O. & Schatz, G. Cytochrome c oxidase from bakers' yeast. IV. Immunological evidence for the participation of a mitochondrially synthesized subunit in enzymatic activity. *J Biol Chem* **250**, 762-766 (1975).
8 Maccecchini, M. L., Rudin, Y., Blobel, G. & Schatz, G. Import of proteins into mitochondria: precursor forms of the extramitochondrially made F1-ATPase subunits in yeast. *Proc Natl Acad Sci U S A* **76**, 343-347 (1979).
9 Gin, P. & Clarke, C. F. Genetic Evidence for a Multi-subunit Complex in Coenzyme Q Biosynthesis in Yeast and the Role of the Coq1 Hexaprenyl Diphosphate Synthase. *J Biol Chem* **280**, 2676-2681 (2005).
10 Belogrudov, G. I. *et al.* Yeast COQ4 Encodes a Mitochondrial Protein Required for Coenzyme Q Synthesis. *Arch Biochem Biophys* **392**, 48-58 (2001).
11 Tran, U. C. *et al.* Complementation of Saccharomyces cerevisiae coq7 Mutants by Mitochondrial Targeting of the Escherichia coli UbiF Polypeptide: TWO FUNCTIONS OF YEAST COQ7 POLYPEPTIDE IN COENZYME Q BIOSYNTHESIS. *J Biol Chem* **281**, 16401-16409 (2006).
12 Hsieh, E. J. *et al.* Saccharomyces cerevisiae Coq9 polypeptide is a subunit of the mitochondrial coenzyme Q biosynthetic complex. *Arch Biochem Biophys* **463**, 19-26 (2007).
13 Daum, G., Böhni, P. C. & Schatz, G. Import of proteins into mitochondria. Cytochrome b2 and cytochrome c peroxidase are located in the intermembrane space of yeast mitochondria. *J Biol Chem* **257**, 13028-13033 (1982).
14 Whited, K., Baile, M. G., Currier, P. & Claypool, S. M. Seven functional classes of Barth syndrome mutation. *Hum Mol Genet* **22**, 483-492 (2013).
15 Glick, B. S. *et al.* Cytochromes c1 and b2 are sorted to the intermembrane space of yeast mitochondria by a stop-transfer mechanism. *Cell* **69**, 809-822 (1992).
16 Djavadi-Ohaniance, L., Rudin, Y. & Schatz, G. Identification of enzymically inactive apocytochrome c peroxidase in anaerobically grown Saccharomyces cerevisiae. *J Biol Chem* **253**, 4402-4407 (1978).
17 Panneels, V., Schüssler, U., Costagliola, S. & Sinning, I. Choline head groups stabilize the matrix loop regions of the ATP/ADP carrier ScAAC2. *Biochem Biophys Res Commun* **300**, 65-74 (2003).

18 Ohba, M. & Schatz, G. Protein import into yeast mitochondria is inhibited by antibodies raised against 45-kd proteins of the outer membrane. *EMBO J* **6**, 2109-2115 (1987).
19 Choi, H. S., Han, G. S. & Carman, G. M. Phosphorylation of yeast phosphatidylserine synthase by protein kinase A: identification of Ser46 and Ser47 as major sites of phosphorylation. *J Biol Chem* **285**, 11526-11536 (2010).
20 Claypool, S. M., McCaffery, J. M. & Koehler, C. M. Mitochondrial mislocalization and altered assembly of a cluster of Barth syndrome mutant tafazzins. *J Cell Biol* **174**, 379-390 (2006).
21 Huyer, G. *et al.* Distinct machinery is required in Saccharomyces cerevisiae for the endoplasmic reticulum-associated degradation of a multispanning membrane protein and a soluble luminal protein. *J Biol Chem* **279**, 38369-38378 (2004).

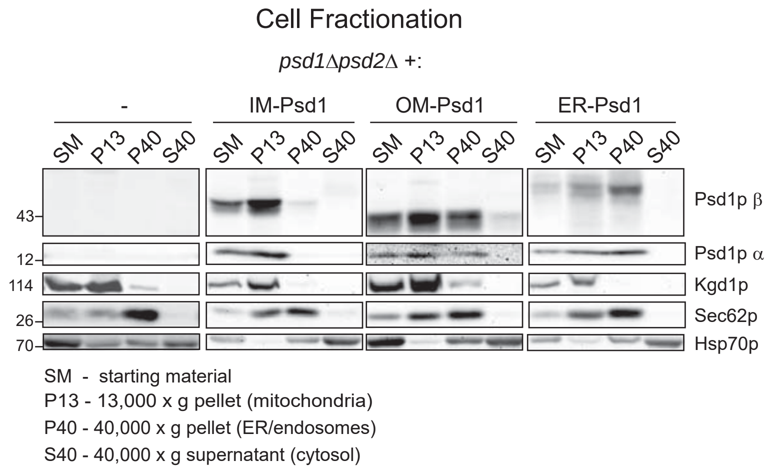

Supplementary Fig 1. ER-Psd1 co-fractionates with ER/endosomal compartment. (A) Following growth in rich lactate medium to late log phase, fractions were collected from the indicated yeast strains by differential gravity centrifugation. Equal protein amounts from each collected fraction were resolved by SDS-PAGE and immunoblotted for Psd1 (β and α subunits) and markers for each compartment (Kgd1 for mitochondria/P13, Sec62 for the ER/P40, and Hsp70 for the cytosol/S40).

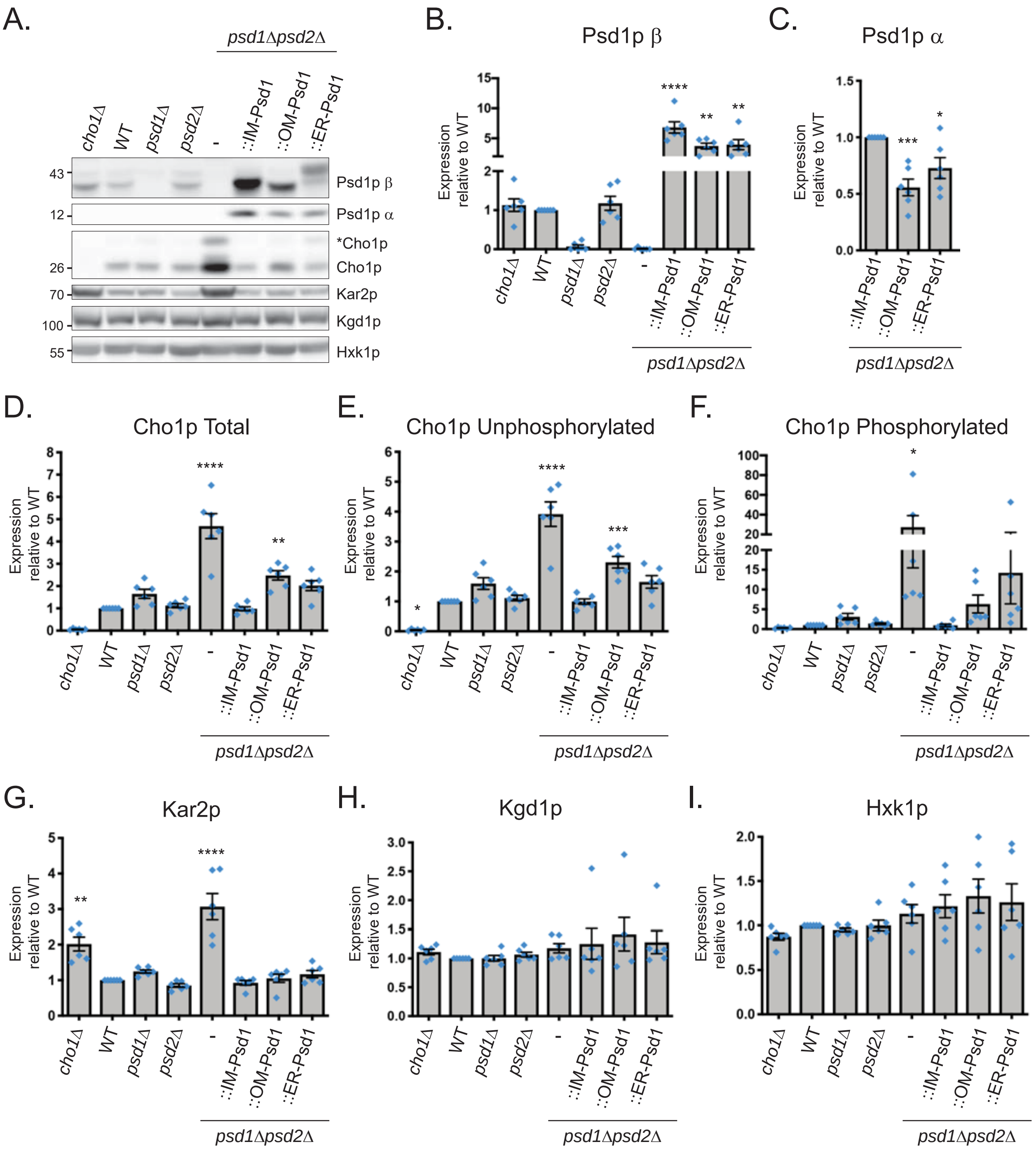

Supplementary Fig 2. Cho1 expression and phosphorylation state is increased in *psd1*Δ*psd2*Δ yeast, which also indicate hallmarks of ER stress through elevated levels of Kar2p. Yeast strains were cultured in rich lactate medium for 2 days at 30°C. Cells were harvested by centrifugation, lysed, protein extracted, resuspended in Laemelli buffer, and analyzed by immunoblot. (B-I) The expression of the indicated proteins was normalized relative to WT (mean ± S.E.M. for n=6 independent experiments). Statistical differences (ns, $P > 0.05$; 1 symbol $P \leq 0.05$; 2 symbols $P \leq 0.01$; 3 symbols $P \leq 0.001$; 4 symbols $P \leq 0.0001$) compared to WT (asterisk) were determined by one-way ANOVA with Tukey's multiple-comparisons test.

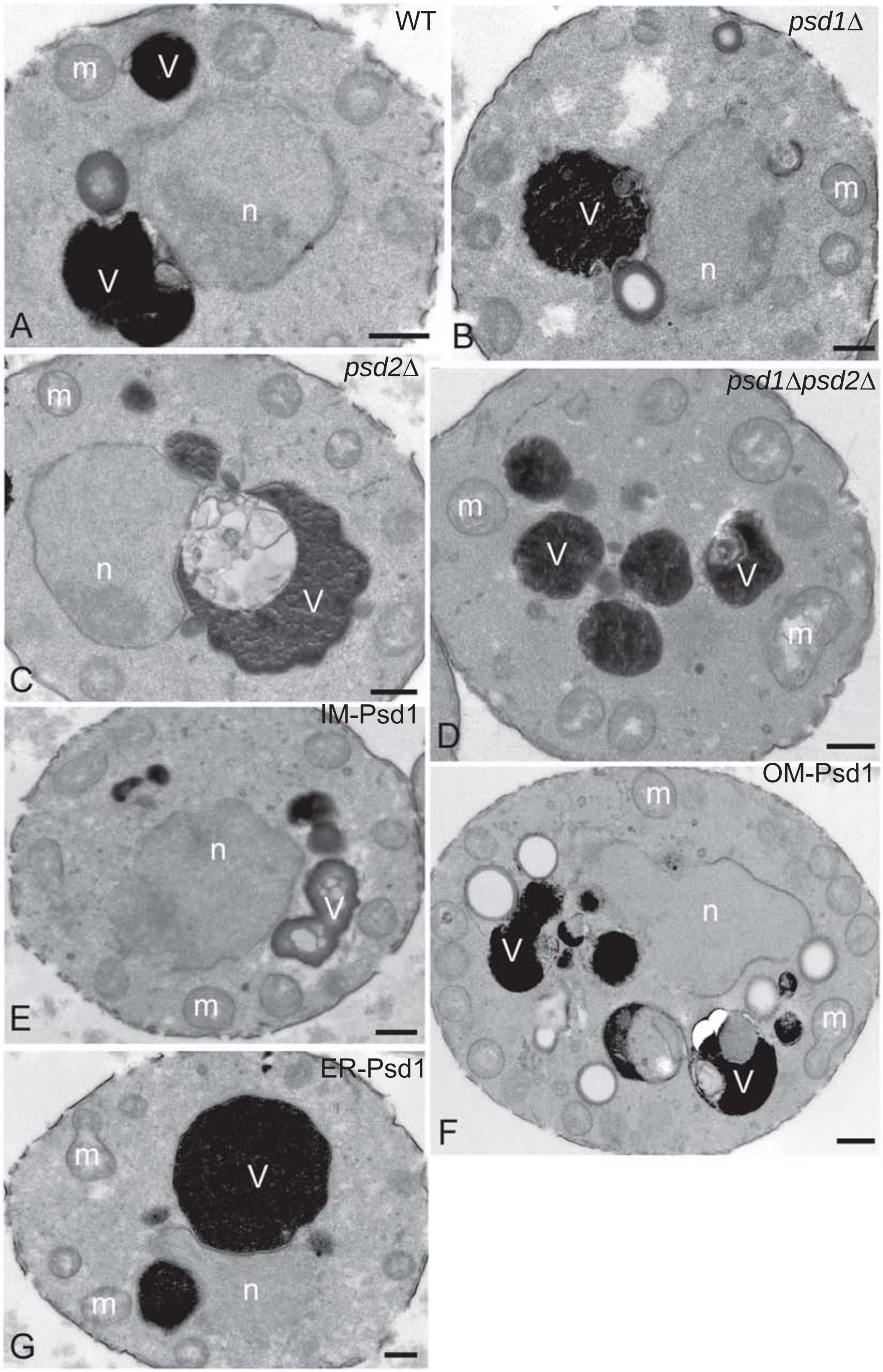

Supplementary Fig 3. Mitochondrial morphology is not overtly affected by disruption or alteration of Psd1 pathway. Cells from the indicated strains were analyzed by transmission electron microscopy. A) GA74-1A parental wildtype strain, B) *psd1*Δ, C) *psd2*Δ, D) *psd1*Δ*psd2*Δ, E) *psd1*Δ*psd2*Δ::IM-Psd1, F) *psd1*Δ*psd2*Δ::OM-Psd1, G) *psd1*Δ*psd2*Δ::ER-Psd1. *n*, nucleus; *m*, mitochondria; and *v*, vacuole. Bars, 0.5 μm.

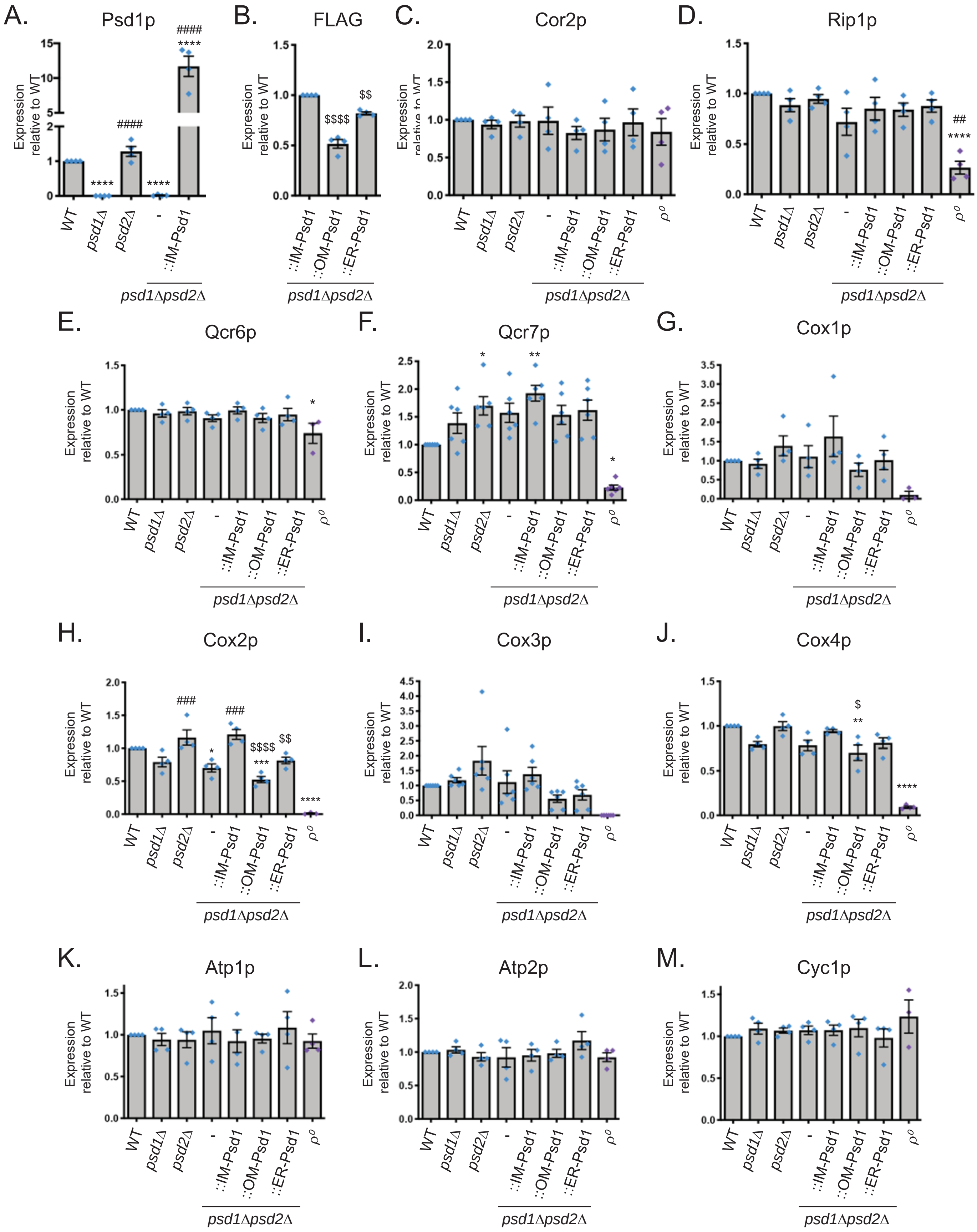

Supplementary Fig 4. Quantitation of respiratory complex subunits. Densitometry analysis of steady state protein amounts in isolated mitochondria (30 μg) from the indicated strains (representative immunoblots shown in Fig. 5C). Protein expression was normalized relative to WT (mean ± S.E.M. for n=4 independent experiments). Statistical comparisons (ns, $P > 0.05$; 1 symbol $P \leq 0.05$; 2 symbols $P \leq 0.01$; 3 symbols $P \leq 0.001$; 4 symbols $P \leq 0.0001$) versus WT (asterisk), *psd1Δpsd2Δ* (number sign), or IM-Psd1 (dollar sign) were determined by one-way analysis of variance (ANOVA) with Tukey's pairwise comparison.

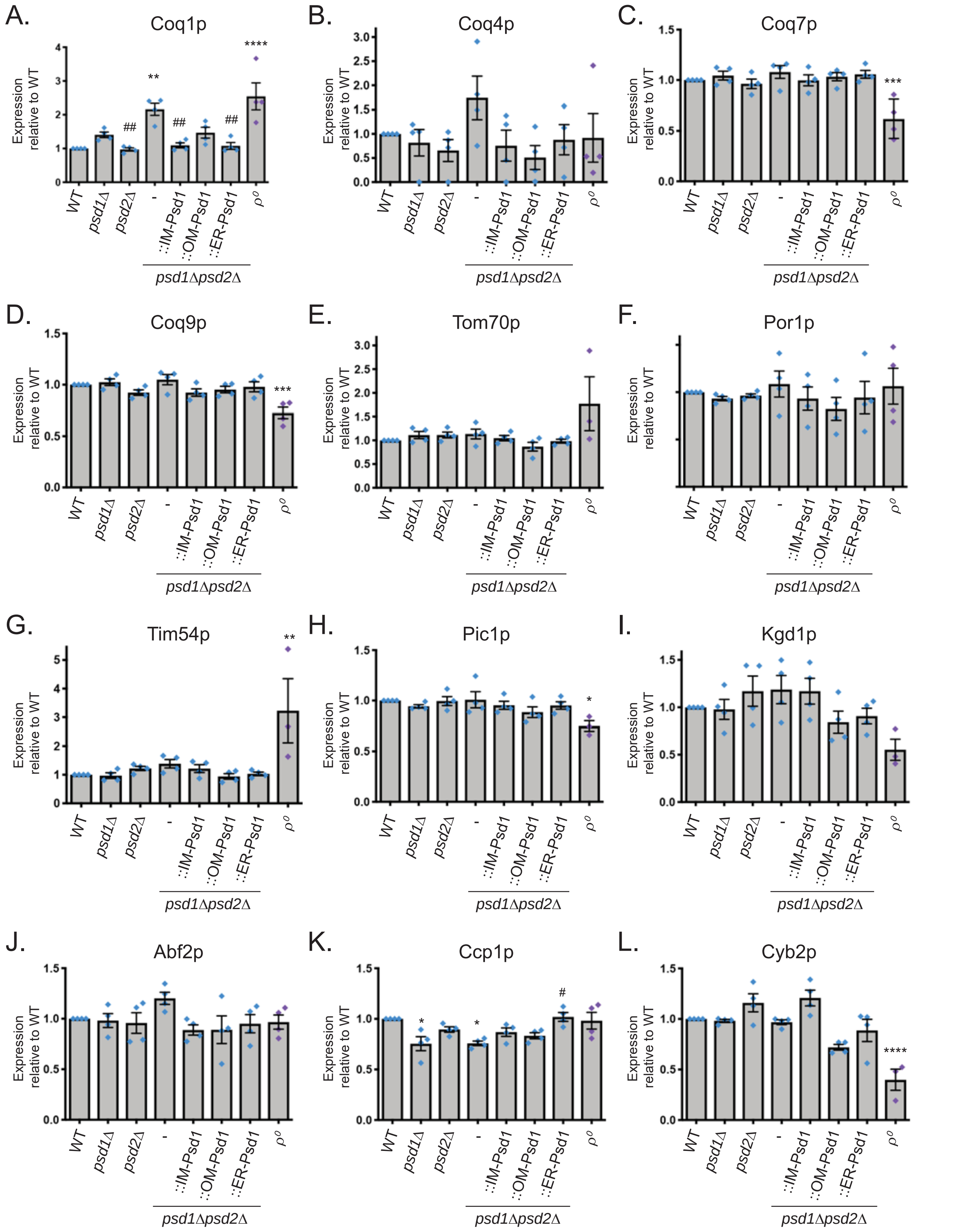

Supplementary Fig 5. Quantitation of CoQ synthome subunits and additional mitochondrial proteins. Densitometry analysis of steady state protein amounts in isolated mitochondria (30 μg) from the indicated strains (representative immunoblots shown in Fig. 5C). Protein expression was normalized relative to WT (mean ± S.E.M. for n=4 independent experiments). Statistical comparisons (ns, $P > 0.05$; 1 symbol $P \leq 0.05$; 2 symbols $P \leq 0.01$; 3 symbols $P \leq 0.001$; 4 symbols $P \leq 0.0001$) versus WT (asterisk), *psd1Δpsd2Δ* (number sign), or IM-Psd1 (dollar sign) were determined by one-way analysis of variance (ANOVA) with Tukey's pairwise comparison.

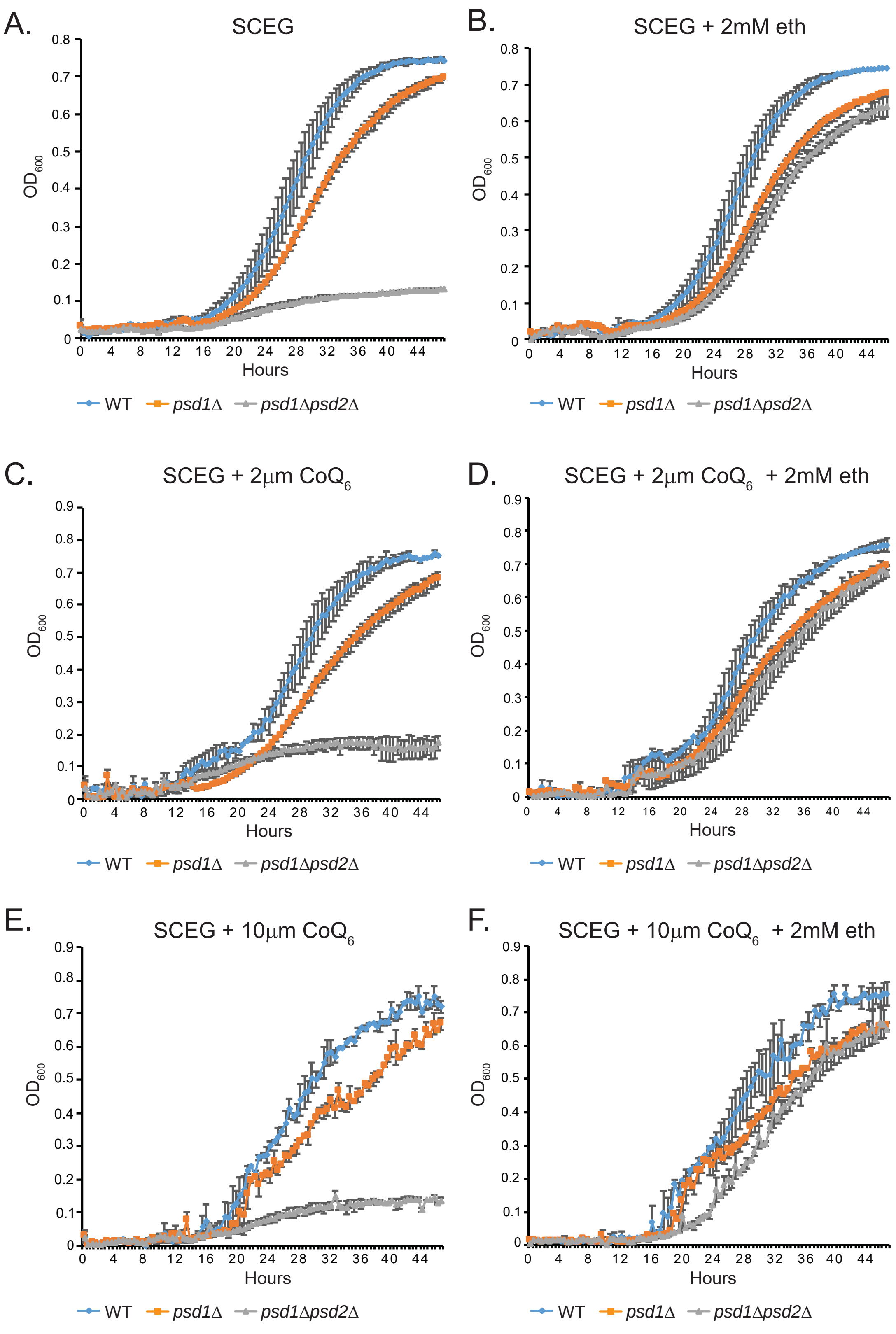

Supplementary Fig 6. CoQ6 supplementation does not rescue the *psd1*Δ and *psd1*Δ*psd2*Δ growth defects on respiratory medium. OD_{600} measurements were recorded every 30 minutes for a period of 48 hours at 30°C for yeast grown in (A) SCEG, (B) SCEG with (+) 2mM ethanolamine (eth), (C) SCEG + 2µM CoQ6, (D) SCEG + 2µM CoQ6 + 2mM ethanolamine, (E) SCEG + 10µM CoQ6, and (F) SCEG + 10µM CoQ6 + 2mM ethanolamine. mean ± S.E.M. for n=2 independent experiments.

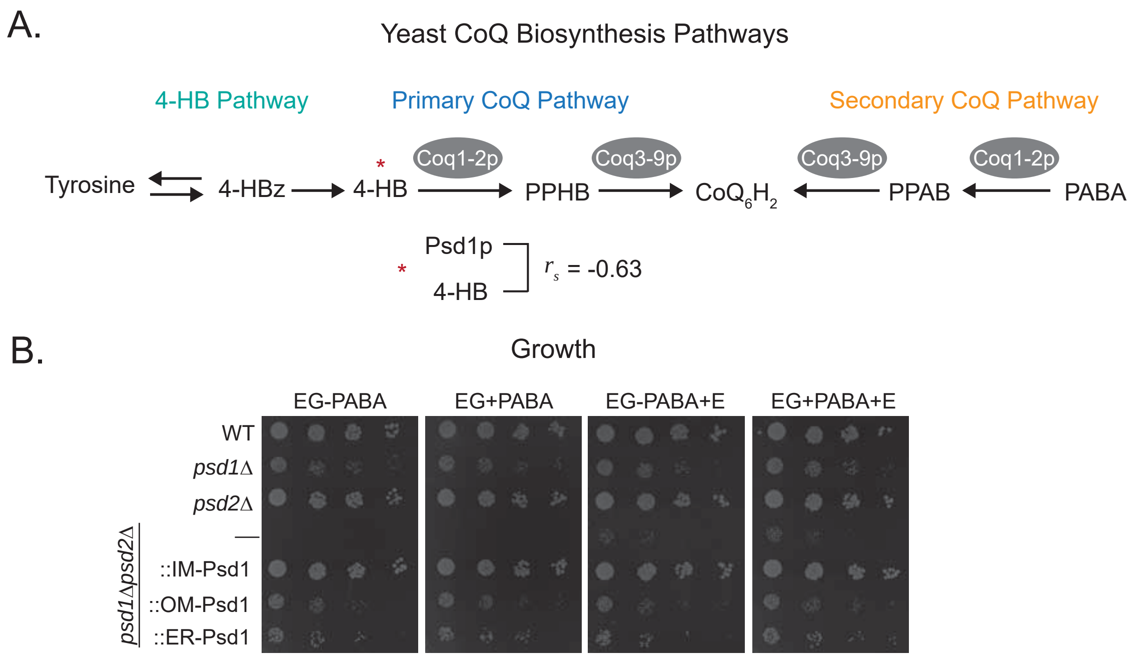

Supplementary Fig 7. The para-amino benzoic acid pathway for CoQ_6 biosynthesis is not necessary for *psd1*Δ growth. (A) Pathways for CoQ Biosynthesis; 4-hydroxy phenylpyruvate (4-HBz), 4-hydroxybenzoate (4-HB), 3-polyprenyl-4-hydroxybenzoate (PPHB), CoQ_6H_2, 3-hexaprenyl-4-aminobenzoate (PPAB), *para*-amino-benzoate (PABA). The indicated Spearman correlation coefficient (rs) for 4-HB molecules that share a negative correlation with Psd1p molecules, was derived from the Yeast 3 Thousand (Y3K) dataset [23]. (B) The indicated strains were spotted and incubated at 30°C for 4 days on SCEG with or without (+/-) PABA and 2mM ethanolamine added as indicated (+E).

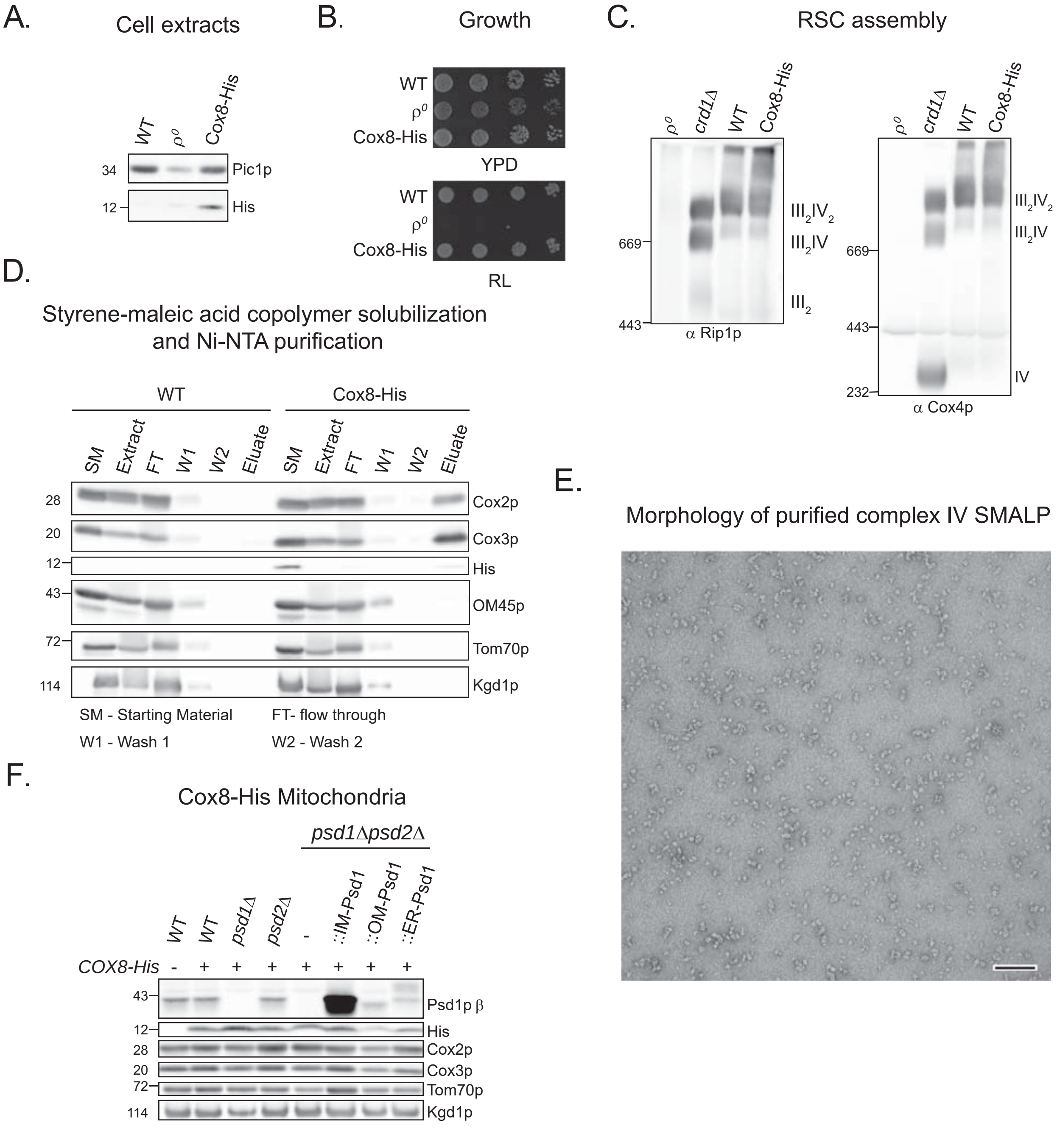

Supplementary Fig 8. Cox8-His is functional, assembles normally, and enables affinity purification of complex IV. (A) Cox8-His was detected in yeast whole cell extracts of the indicated strains by immunoblot. Pic1 served as a loading control. (B) The indicated strains were spotted and incubated at 30°C for 2 days on YPD and for 3 days on rich lactate (RL). (C) Blue native-PAGE analysis of respiratory supercomplexes (RSCs) using mitochondrial extracts solubilized in 1.5% (w/v) digitonin. Complex III assembly was monitored by immunoblot against the nuclear-encoded subunit Rip1 and complex IV assembly was monitored by immunoblot against the nuclear-encoded subunit Cox4. Mitochondria lacking CL (*crd1*Δ) were used as a positive control for RSC destabilization. (D) SMA extracts from WT yeast lacking or expressing Cox8-His were affinity purified using Ni-NTA agarose, washed sequentially with wash buffer containing 10 mM and 20 mM imidazole, and bound material eluted with 60 mM imidazole. Equal amount of the indicated fractions were resolved by SDS-PAGE and immunoblotted for complex IV subunits (His, Cox2, and Cox3), two abundant OM proteins (OM45 and Tom70), and Kgd1. (E) Electron micrograph of affinity purified CIV-SMALPs at a magnification of 98,000x (scale bar = 100 nm). (F) Mitochondria from the indicated strains were immunoblotted as designated.

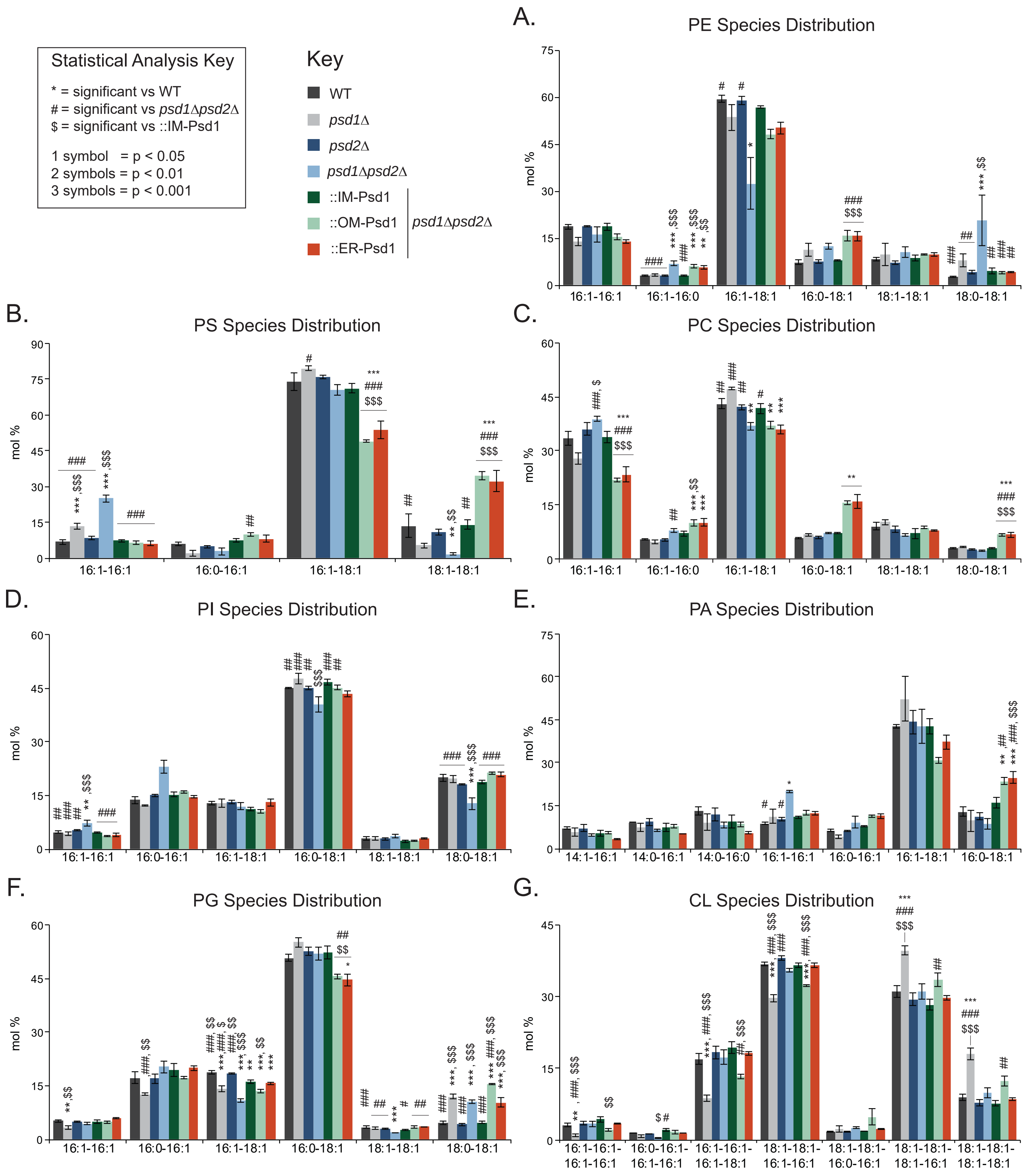

Supplementary Fig 9. The acyl chain pattern of complex IV-associated phospholipids is altered when Psd1 is not in the IM. The acyl chain composition of (A) PE, (B) PS, (C) PC, (D) PI, (E), PA, (F) PG, and (G) CL was determined by shotgun lipidomics and expressed as a % of the total for each lipid class (mean ± S.E.M., n = 3 biologically independent experiments, except for OM-Psd1, n = 4). The color-key for each source of complex IV nanodisc and the symbols for statistical analysis interpretation by one-way ANOVA with Holm-Sidak pairwise comparison is shown at *upper left*. Statistical comparisons (ns, $P > 0.05$; 1 symbol $P \leq 0.05$; 2 symbols $P \leq 0.01$; 3 symbols $P \leq 0.001$; 4 symbols $P \leq 0.0001$) versus WT (asterisk), *psd1Δpsd2Δ* (number sign), or IM-Psd1 (dollar sign) were determined.

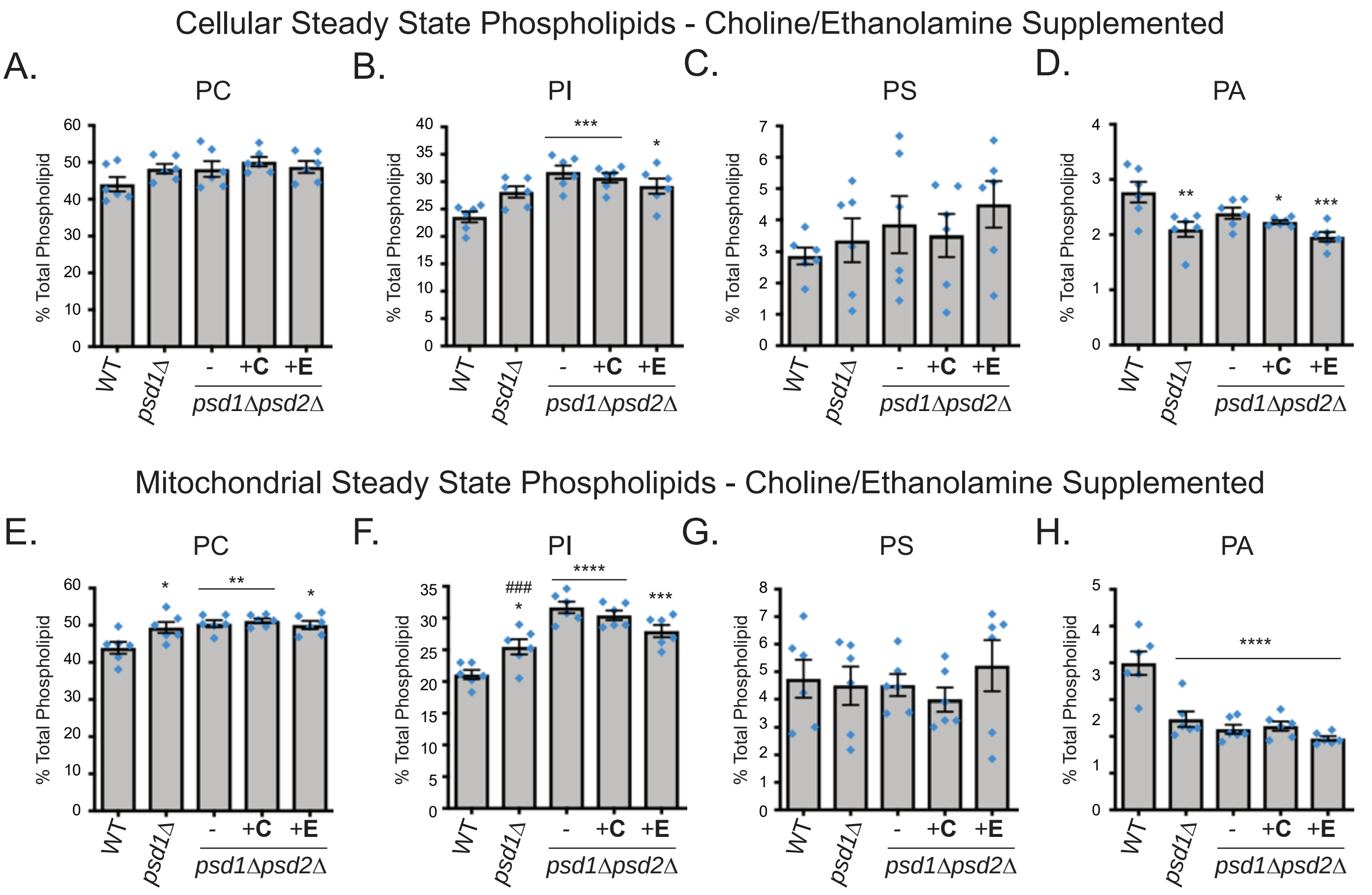

Supplementary Fig 10. Cellular and mitochondrial phospholipid profiles from choline (+C) and ethanolamine (+E) supplemented *psd1Δpsd2Δ* yeast. (A-D) Cellular and (E-H) mitochondrial phospholipids from the indicated strains were labeled overnight with ^{32}Pi and separated by TLC. All graphs show the mean ± S.E.M. for n=6 biological replicates. Significant differences (ns, $P > 0.05$; 1 symbol $P \leq 0.05$; 2 symbols $P \leq 0.01$; 3 symbols $P \leq 0.001$; 4 symbols $P \leq 0.0001$) versus WT (asterisk) or *psd1Δpsd2Δ* (number sign) were determined by one-way analysis of variance (ANOVA) with Tukey's pairwise comparison.

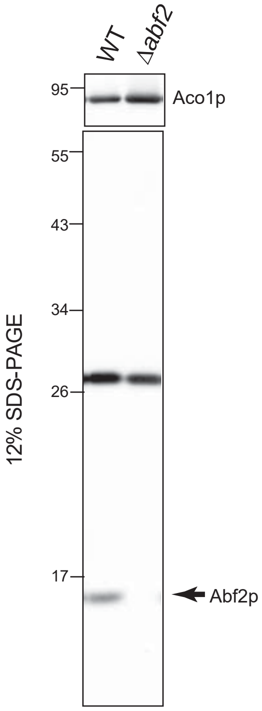

Supplementary Fig 11. Specificity of Abf2-specific antisera. 25 μg of isolated mitochondria from the indicated yeast strains were immunoblotted using antisera raised againts His_6Abf2.Aco1p served as a loading control.

Supplementary Figure 12

Notes for raw files of western blots:

.ai Type size: 5pt

.psd box size: 0.75pt

Inserted jpeg files of .psd files place in .ai

Fig. 2B Original uncropped file; unadjusted exposure:

-Dated 2015_08_14 YS ER OMPsd1 n=2; 2min exposure used. Inverted template to show as FluorChem Q (Cell Biosciences,Santa Clara, CA) quantitative digital imaging system original file.

Psd1p β, α, and Tom70p indicated in fig. Immunoblot exposures were adjusted after cropping of each red boxed blot.

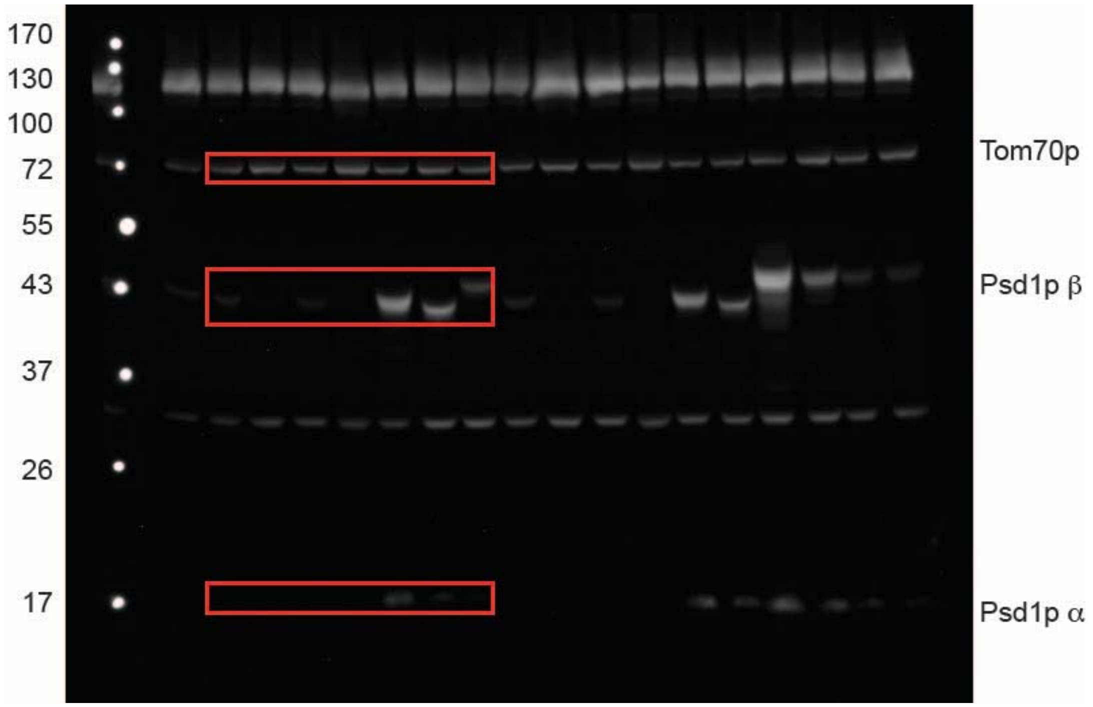

Data not shown in Figure 2B (not redboxed): immunodetection of Kgd1p at ~125kDa, Aac2p at ~30kDa; n=3 yeast cell steady state protein expression (right panel)

Fig. 2D Original uncropped file; unadjusted exposure:

-Dated 2017_06_07 Submito ProtK fractionation; composite of different exposures: 4min Tom70, 8min Tim54, Psd1, FLAG. Inverted template to show as FluorChem Q original file.

Exposures were adjusted after cropping of each red boxed blot.

Note: Edge of Psd1 β blot was cut with razor and resulted in "line" present between the Psd1 β protein band in the ER-Psd1 deoxycholate minus protease sample.

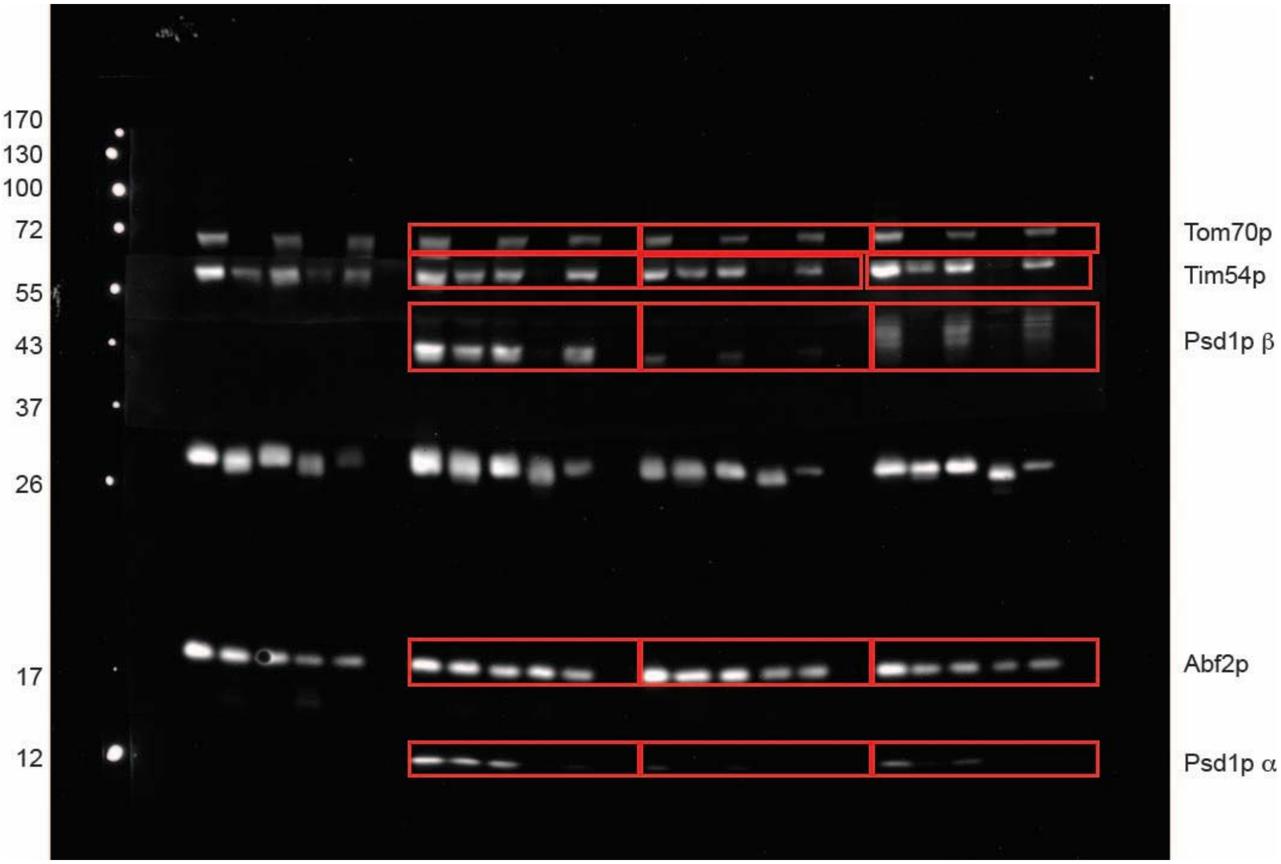

Data not shown in Figure 2D: Aac2p (IM) protease exposure blots; Δ*psd1*Δ*psd2* subcellular fractionation (left panel)

Fig. 3A Original uncropped file; unadjusted exposure:

-Dated 2016_09_05 Sucrose Gradient purified mitos 1; composite of different exposures: 4min Tom70, 8 min Psd1p, Flag, Dpm1p, and Abf2p. Inverted template to display as FluorChem Q original file.

Individual blot exposures were adjusted after cropping of each red boxed blot.

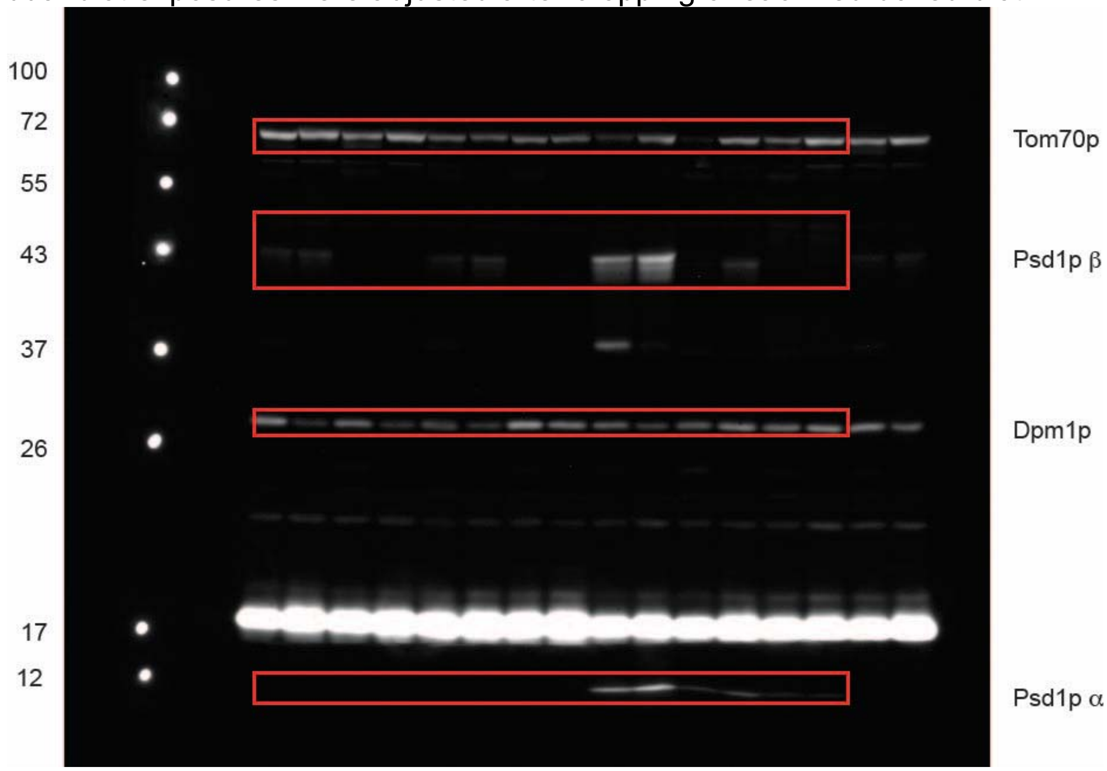

Data not shown in Fig 3A: Abf2p immunoblot

Fig. 5C Original uncropped file; unadjusted exposure:
-Some representative immunoblots were imaged on different days.

Autoexposure function on LiCOR was used to image the indicated immunoblots (white background).

Individual blot exposures were adjusted after cropping of each red boxed blot. Only red boxed blots were used in representative image.

Note: exposed image does not reveal molecular weight markers; protein molecular weights were estimated from molecular weight color standard marked on nitrocellulose membrane

2016_04_04 - Set 1 blots:
Membrane 1: Atp1p, Atp2p, Por1p, Abf2p
Membrane 2: Cor2p, Cox4p

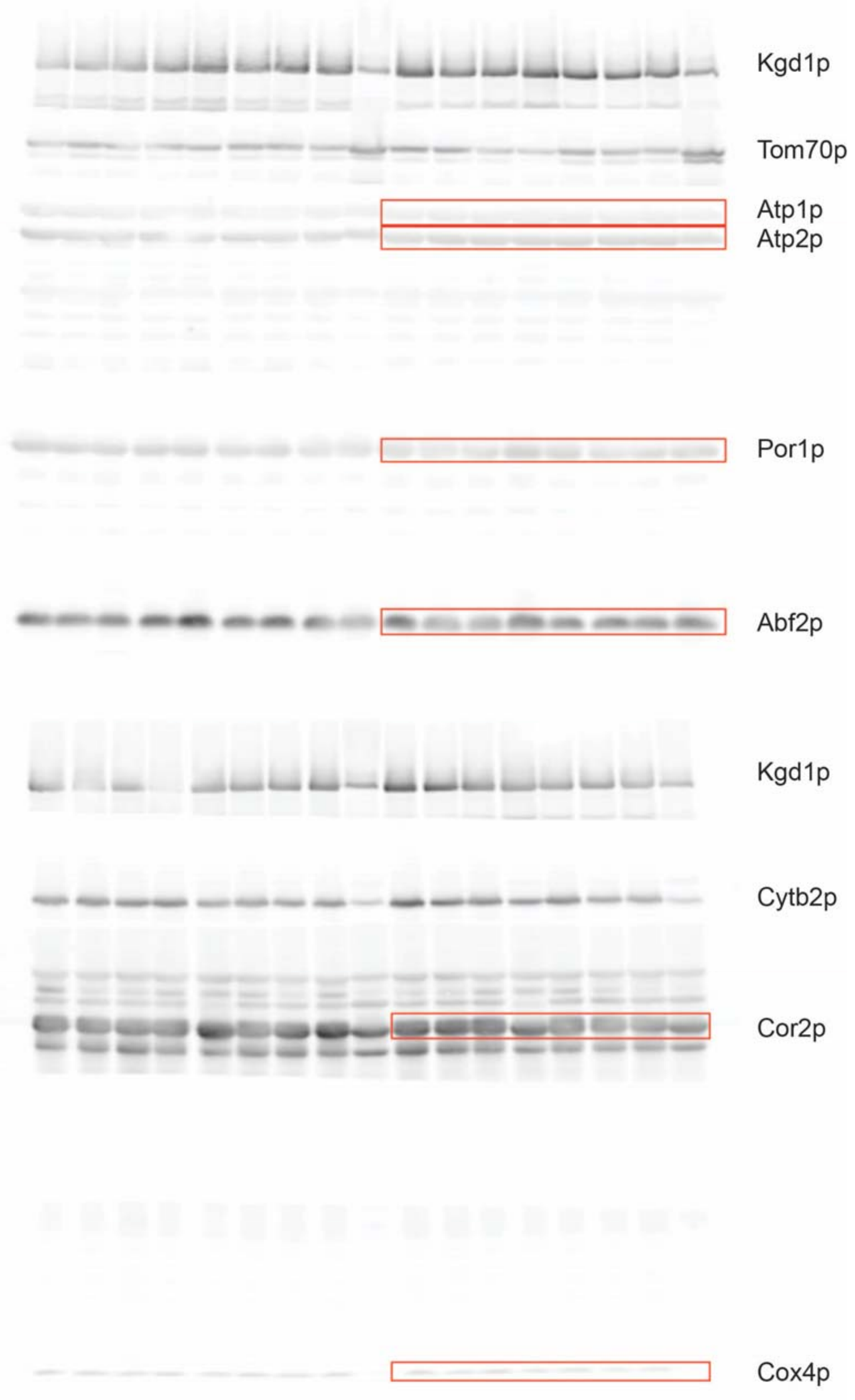
Kgd1p
Tom70p
Atp1p
Atp2p
Por1p
Abf2p
Kgd1p
Cytb2p
Cor2p
Cox4p

Data not used for representative image, not redboxed:

Membrane 1:
Kgd1p, Tom70p

Membrane 2:
Kgd1p, Cytb2

2016_04_04 Set 2: Ccp1p and Rip1p blots:

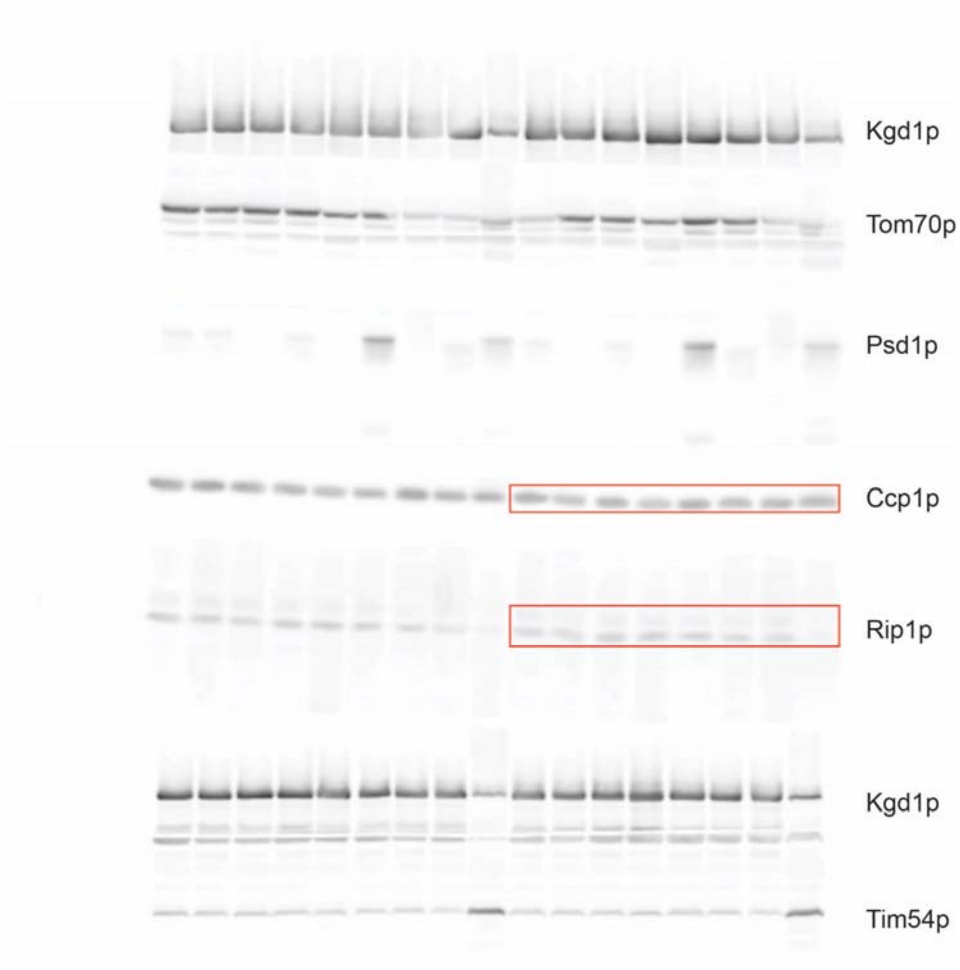

Data not used for representative image (not redboxed):

Membrane 1:
Kgd1p, Tom70p, Psd1p β

Membrane 2:
Kgd1p, Tim54p.

2016_11_09 Set 3 blots:
Membrane 1: Kgd1p, Cytb2p, Psd1p β, Cox2p, Qcr6p
Membrane 2: Tom70p, Cox1p, Pic1p

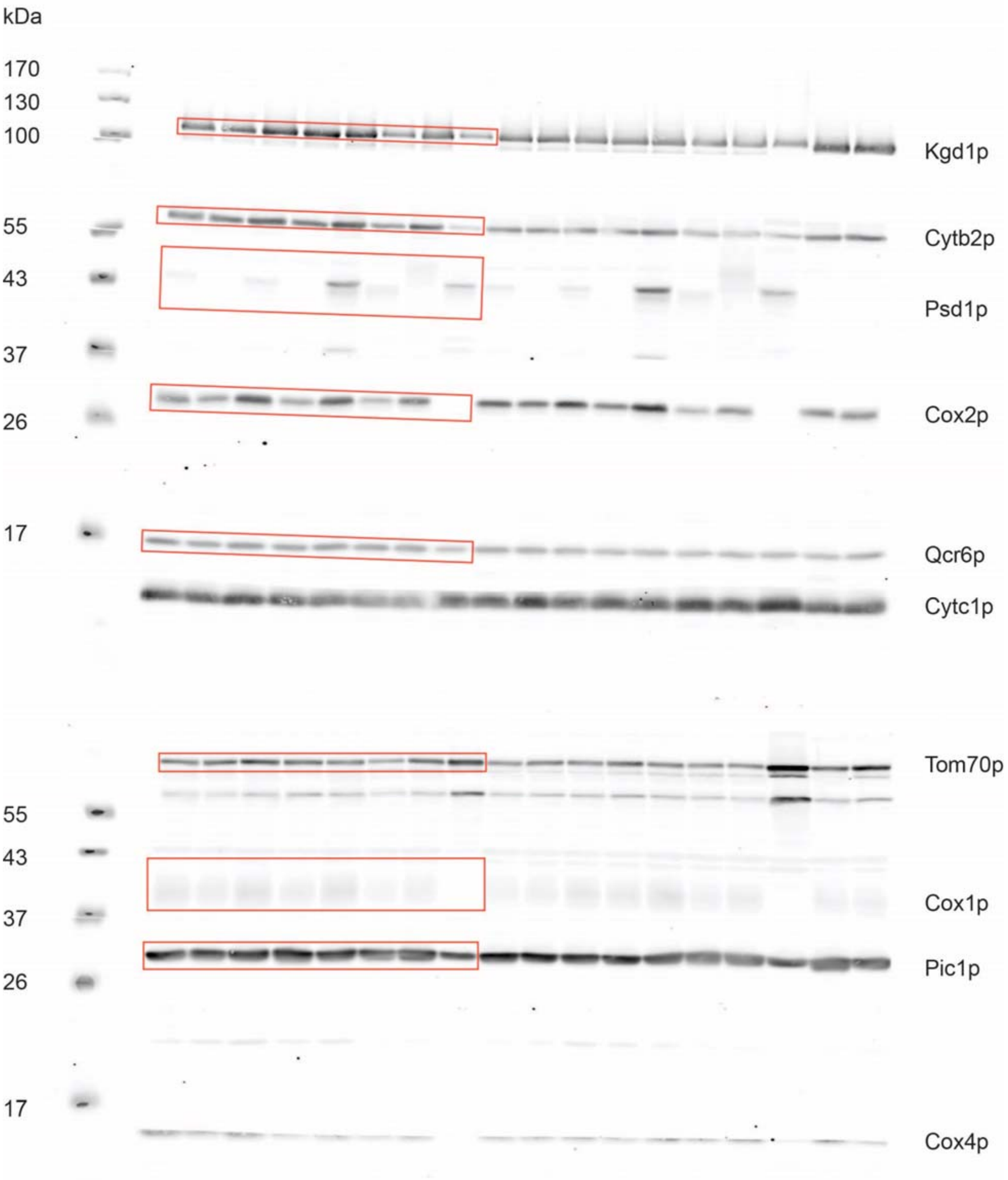

Data not represented in figure (not redboxed) : Cyc1p, Cox4p blots

2017_03_02 Set 4 blots:
Membrane 1: Coq1p, Coq4p, Coq7p
Membrane 2: Coq9p, FLAG (Psd1p α)

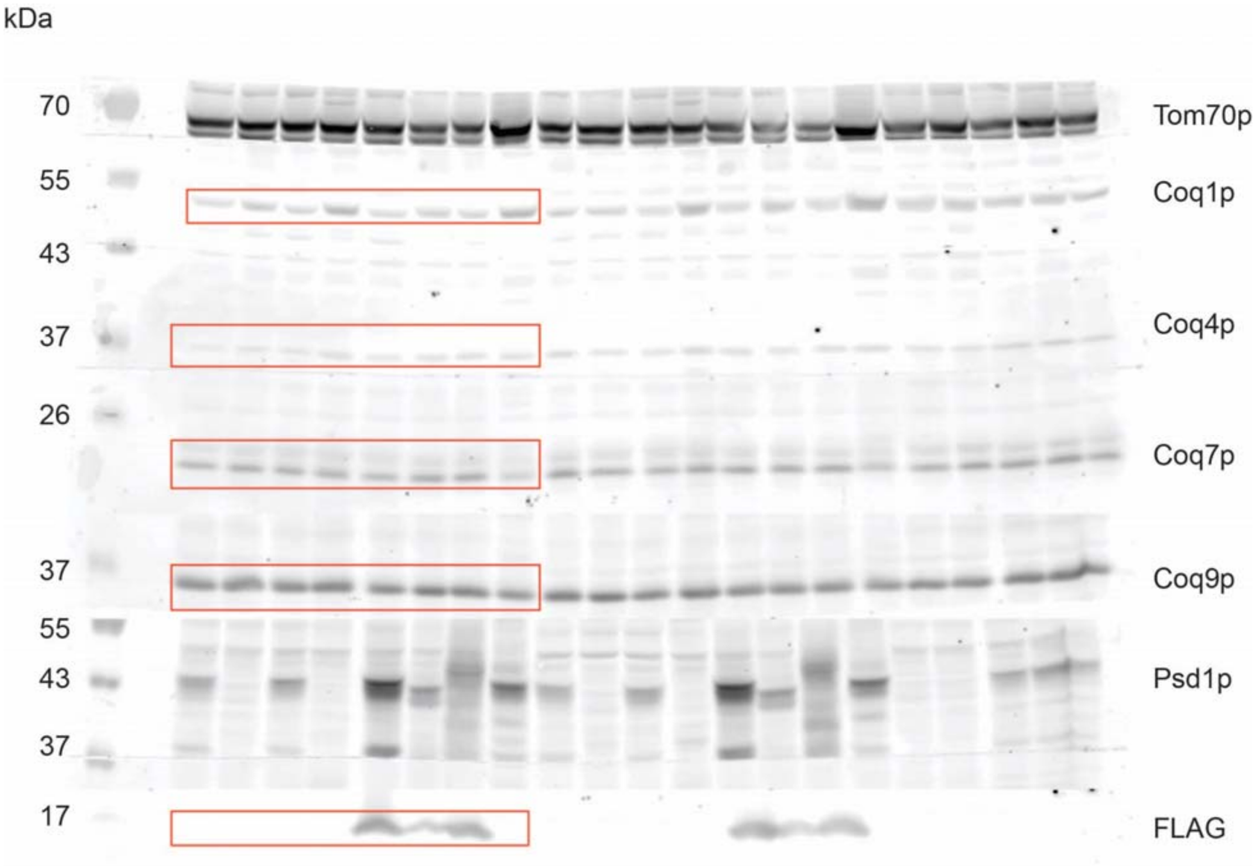

Data not presented in figure (not redboxed): Tom70p, Psd1p β blots

2017_07_04 Set 4 blots:
These immunoblots were imaged on the FluorChem Q.

Exposure of each image was as follows:

Membrane 1: Tim54p (1 min), Cox3p (4min), Qcr7p (4min)

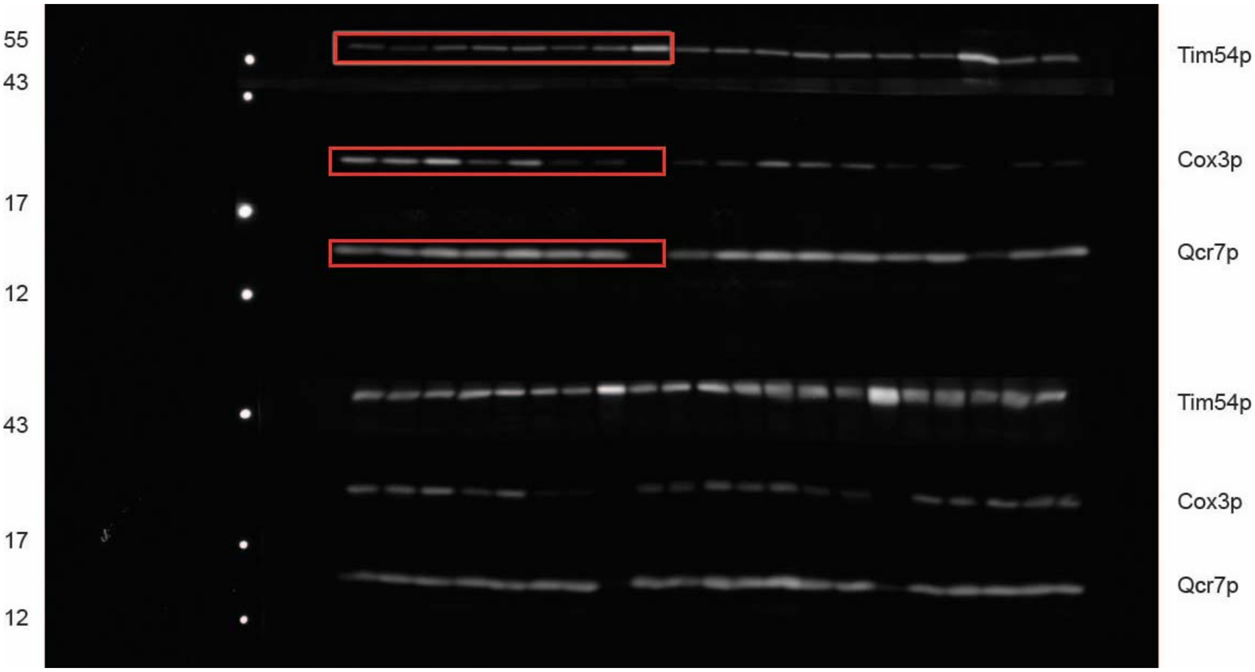

Data not presented in figure (not redboxed): second set of blots (bottom)

Fig. 5D Original uncropped file; unadjusted exposure:
<u>2017_03_30 Cox4p blot</u>

This immunoblot was imaged on the FluorChem Q.

Exposure of Cox4p detection was 16min.

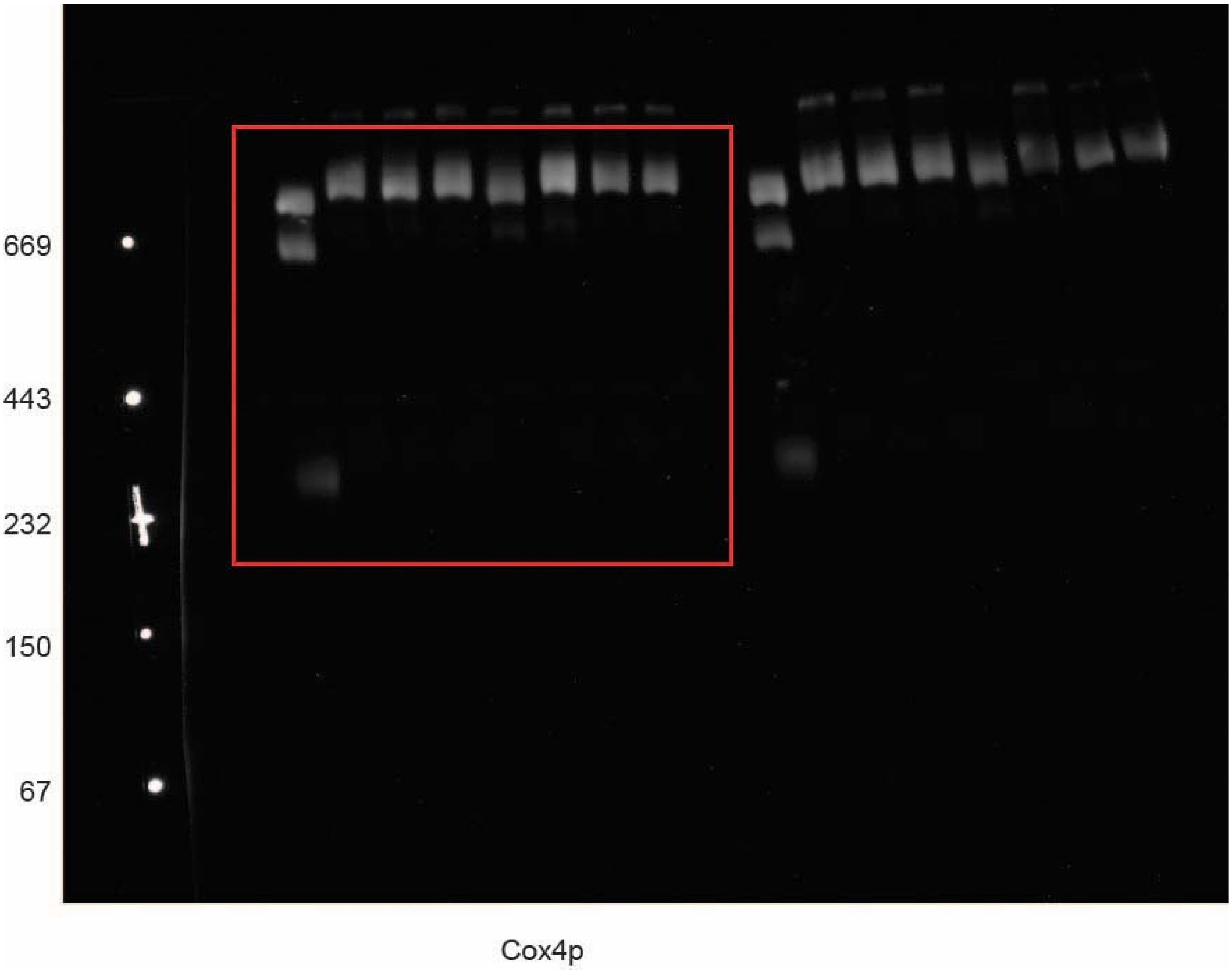

Fig. 5E Original uncropped file; unadjusted exposure:

<u>2017_10_27 Rip1p blots:</u>
2min exposure used on FluorChem Q

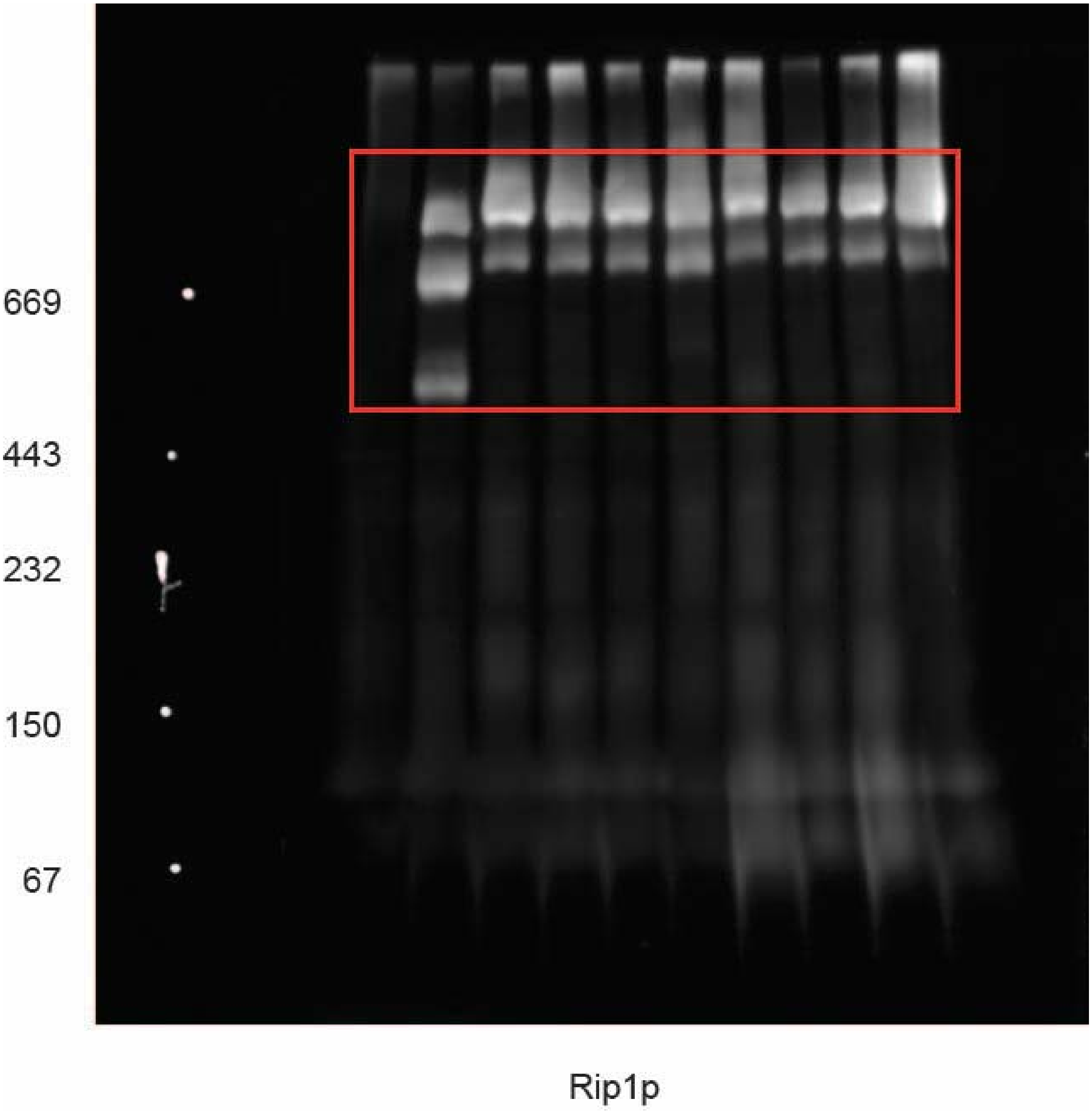

Fig. 6B Original uncropped file; unadjusted exposure:
Immunoblots were imaged on the FluorChem Q
SMA Affinity Purifications 10_20_18; n=3
Individual blot exposures were adjusted after cropping of each red boxed blot.

Blot 1: Composite exposures of: Tom70p (2min), OM45p (30min), Cox3p (2min), and anti-His (30min)

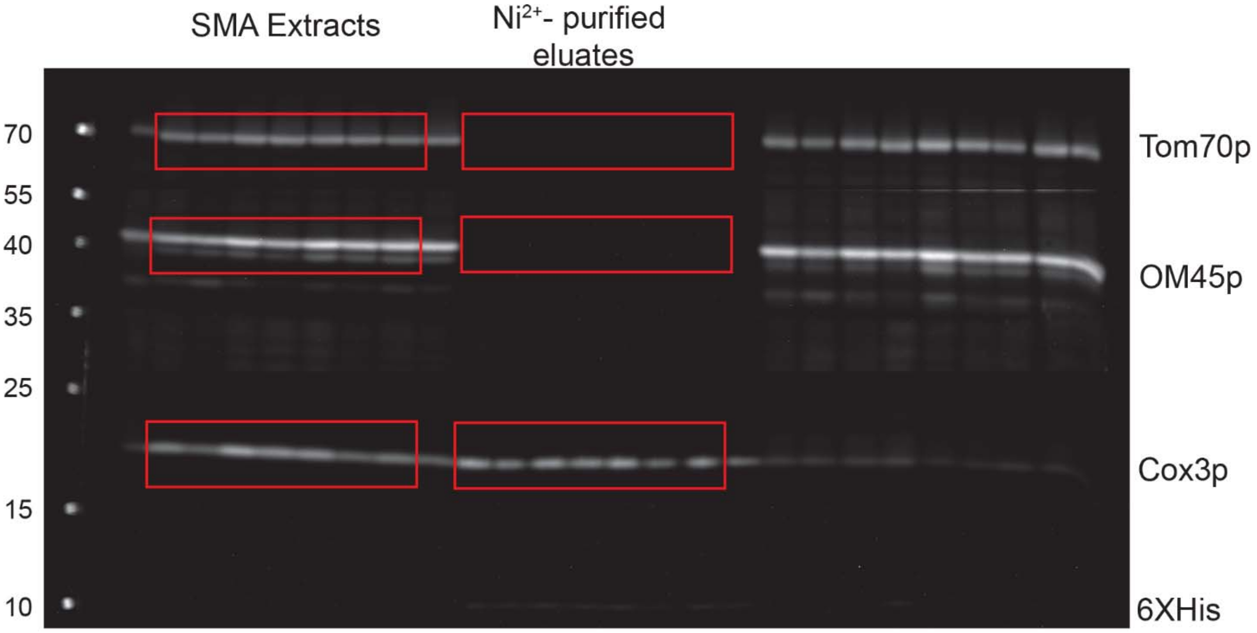

Blot 2 (Strip and Reprobe of above immunoblot): Representative exposure (2min)

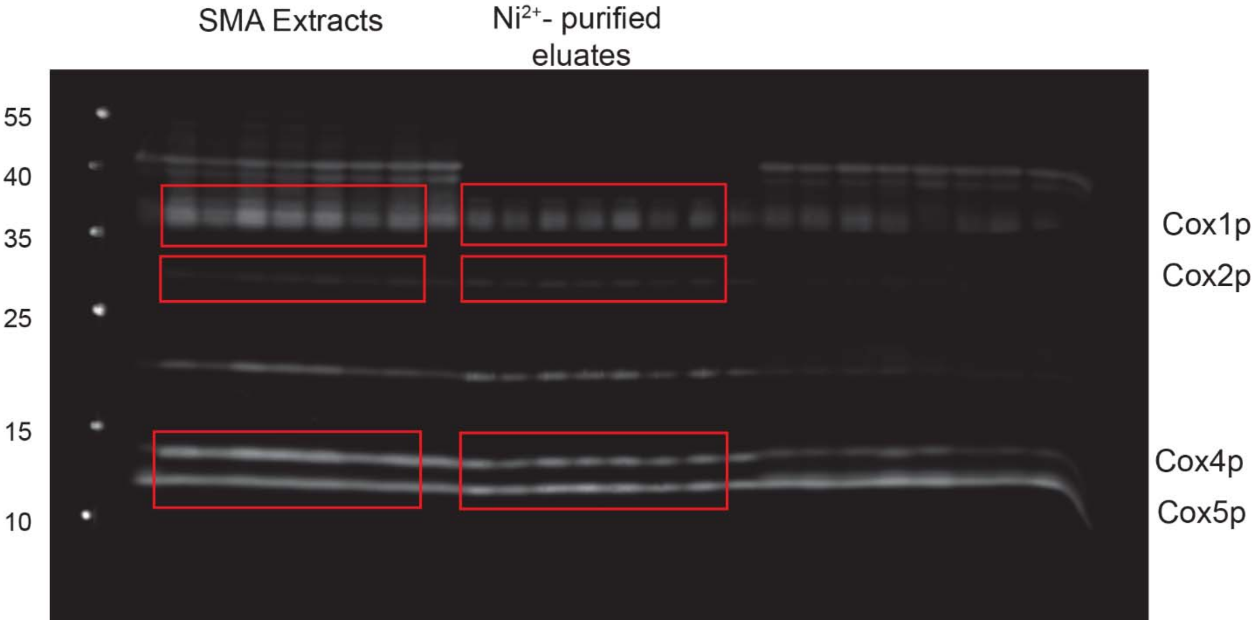

Blot 3 (Strip and Reprobe of above immunoblot; 2nd total strip and reprobe): Composite exposures of: Cor2p (30sec), Por1p (2min), Qcr6p (2min).

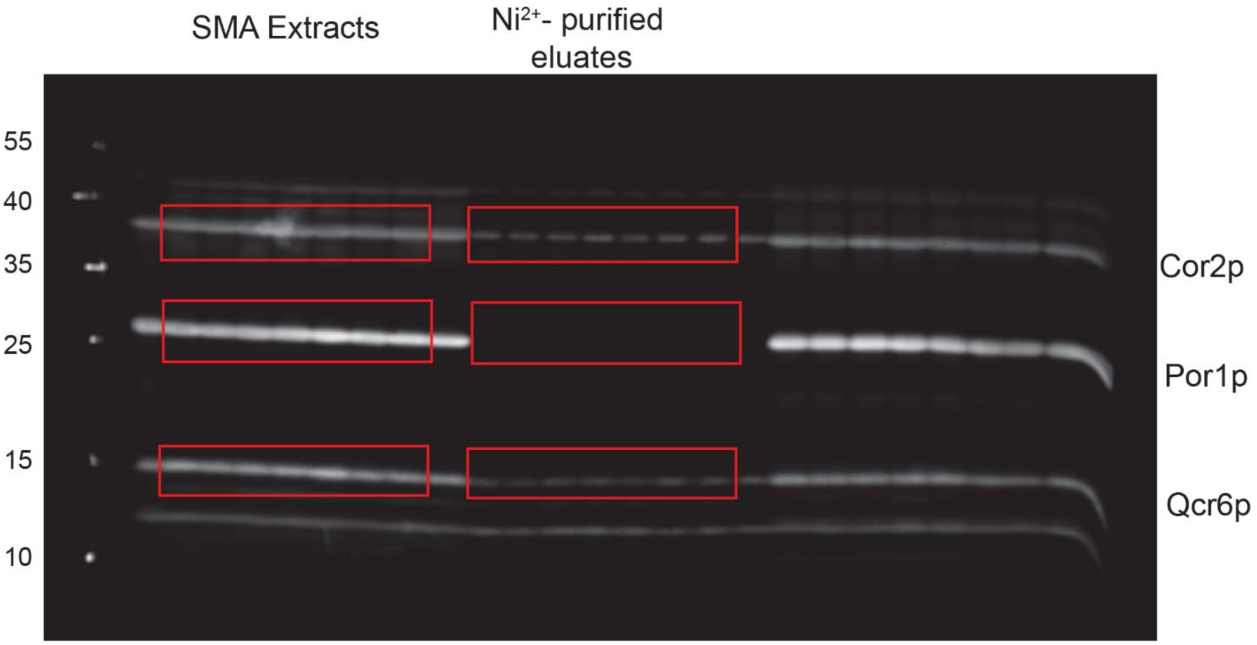

Fig. 8B Original uncropped file; unadjusted exposure:

Dated YS n2, 2018_02_27

FluorChem Q composite image of:
30sec Kgd1 exposure, 4min Cho1p exposure, and 30min Psd1p β exposure

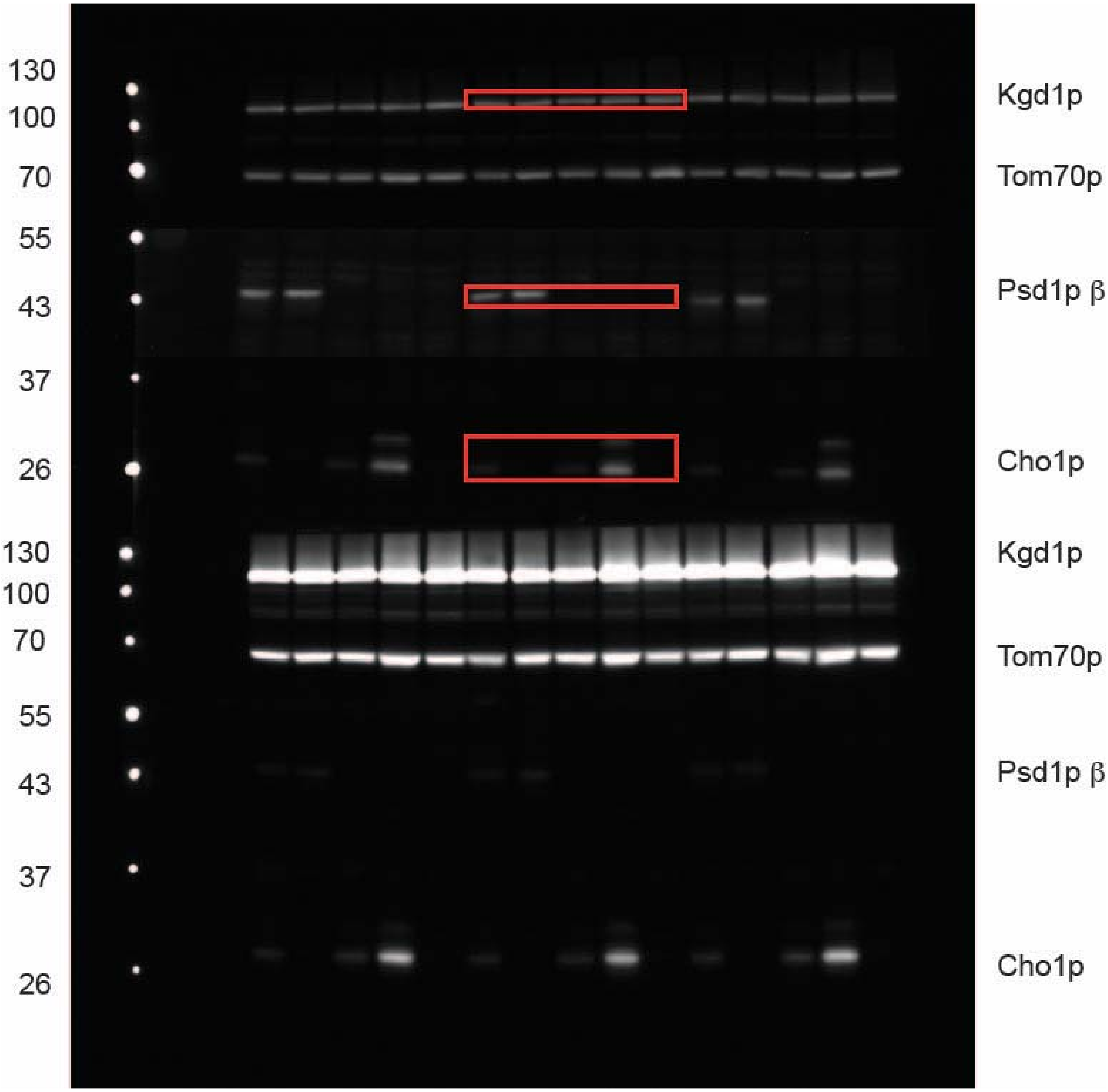

Data not shown in redboxed image: Tom70p immunoblot, and n=1 immunoblots (bottom)

Fig. 8N Original uncropped file; unadjusted exposure:

Dated ss mitos n4, 2018_03_19

FluorChem Q composite exposures for:

Blot 1: Cor2p (15sec), Cox2p (2.5sec), Cox3p (2.5sec), Cox4p (30sec)

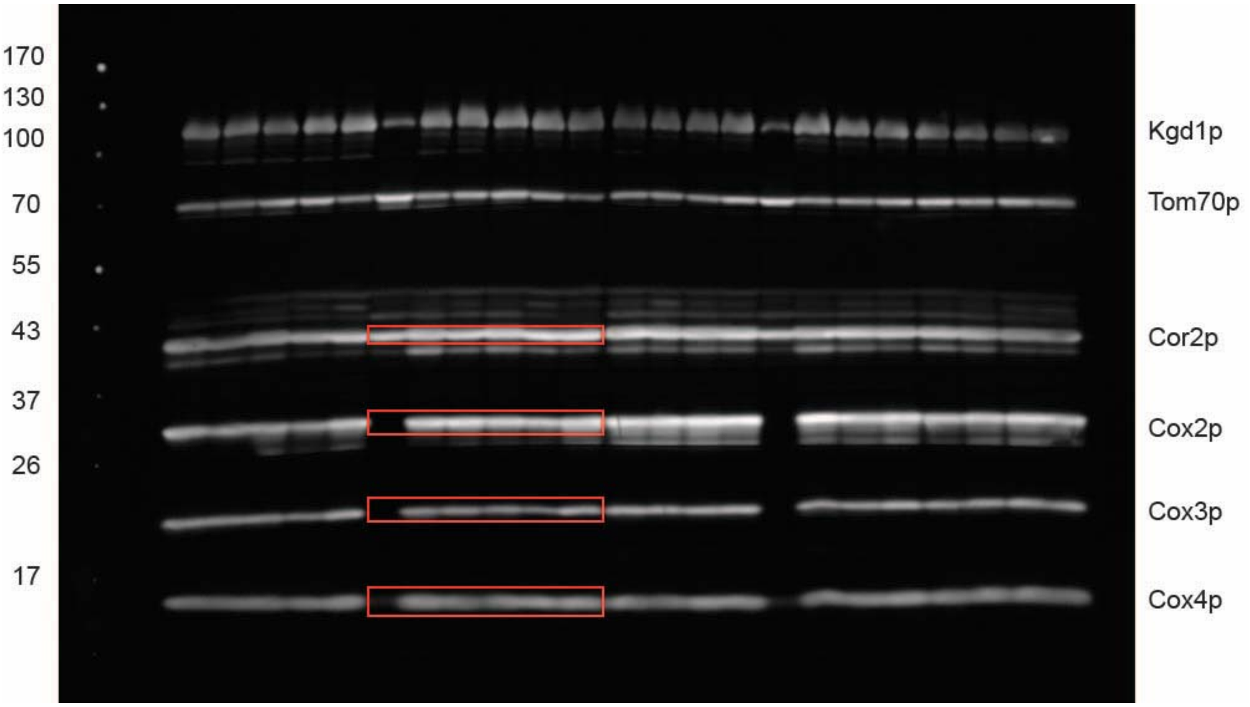

Blot 2: Kgd1p (30sec), Tom70p (30sec) Psd1p β (30sec), Qcr6p (1min)

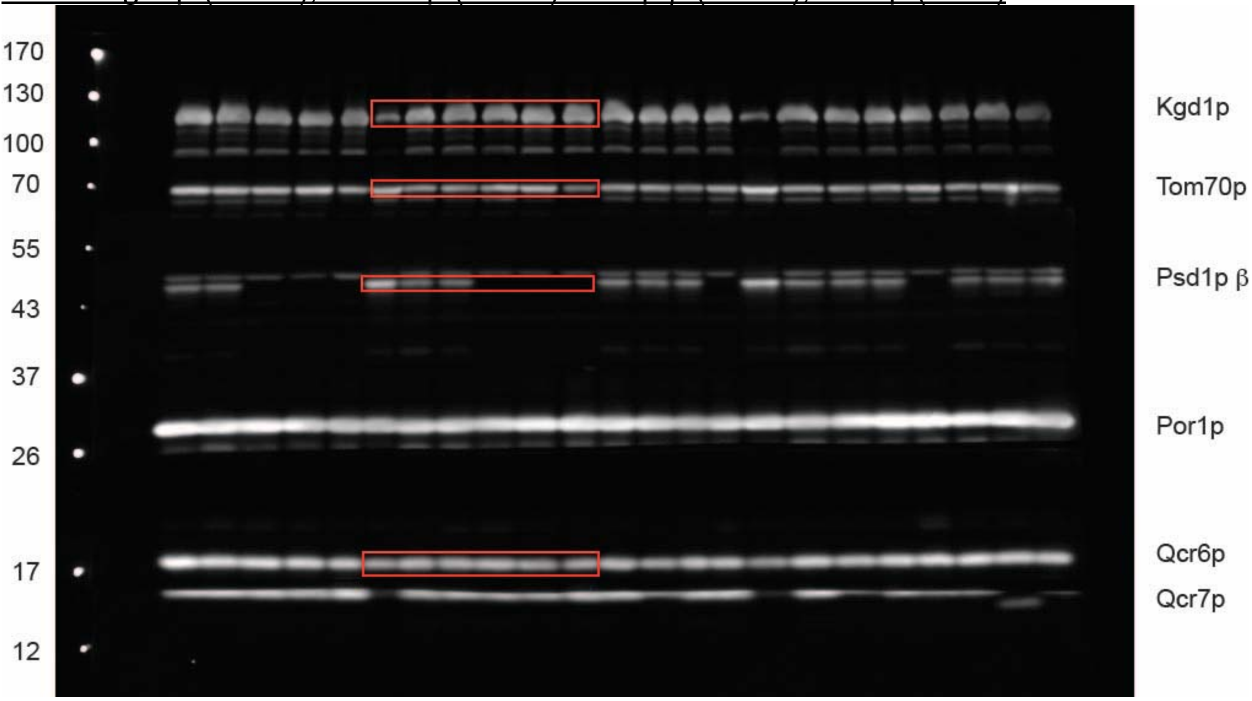

Blot 3 (Strip and Reprobe of above two immunoblots):

Composite exposures of: Atp2p (1min), Rip1p (1min), Aac2p (30sec), Qcr7p (2min)

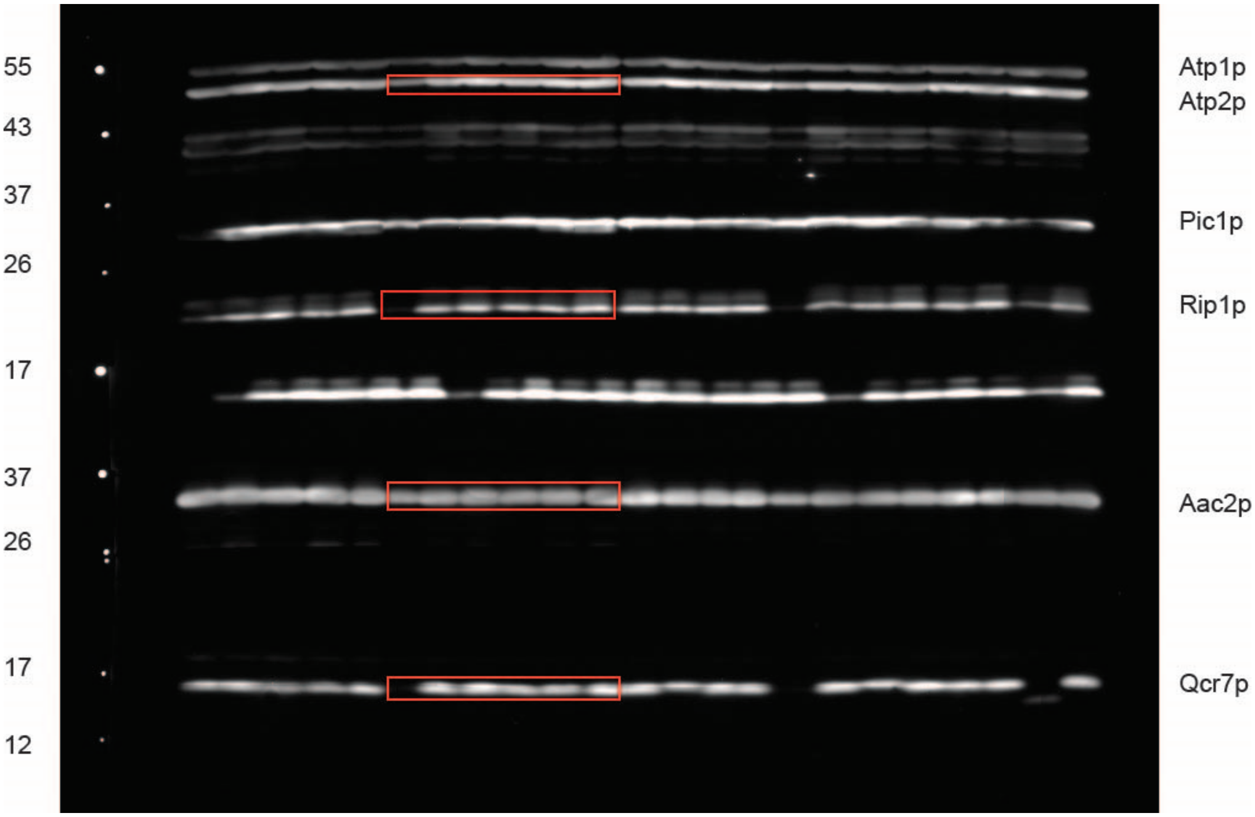

Note: Shorter duration of exposure times due to increased strength of enhanced chemiluminescence (ECL) reagents

Fig. 9B Original uncropped file; unadjusted exposure:
Dated 2018_07_26 YS dQcr7

Composite exposures of 1min Qcr7p, 2min Aac2p, 8min Psd1p

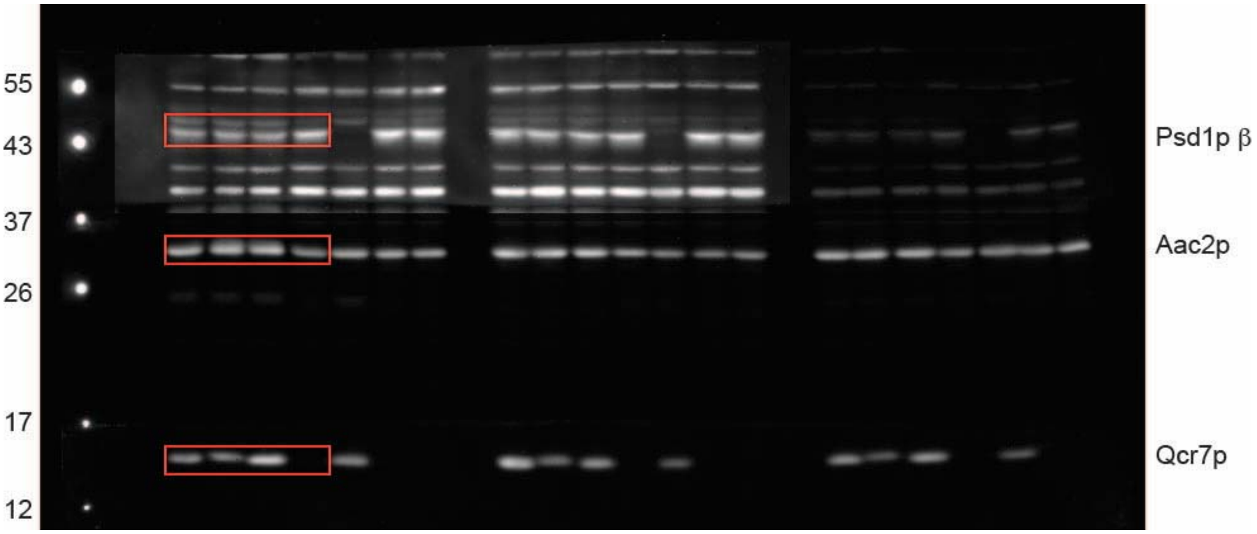

Fig. 9C Original uncropped file; unadjusted exposure:

Dated 2018_03_19 SS mito
Note: imaged on same nitrocellulose membrane as Fig 8n:
FluorChem Q composite exposures for:

Blot 1: Cor2p (15sec), Cox2p (2.5sec), Cox4p (30sec)

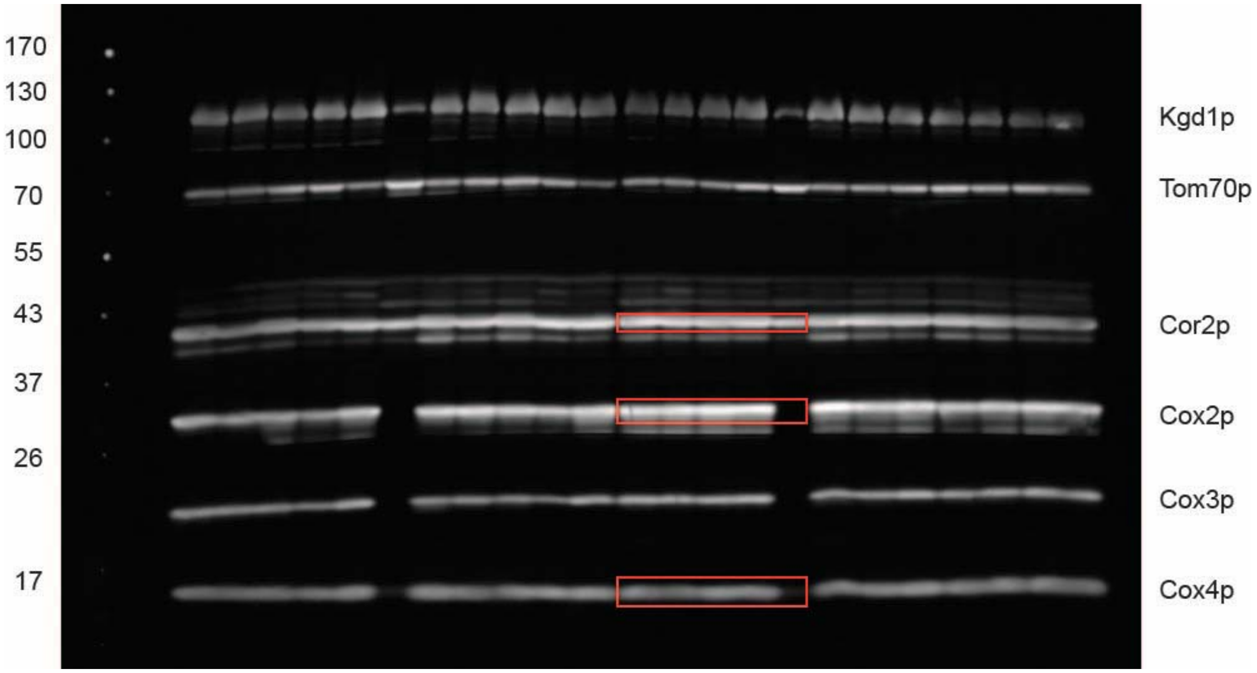

Blot 2: Kgd1p (30sec), Tom70p (30sec) Psd1p β (30sec), Qcr6p (1min)

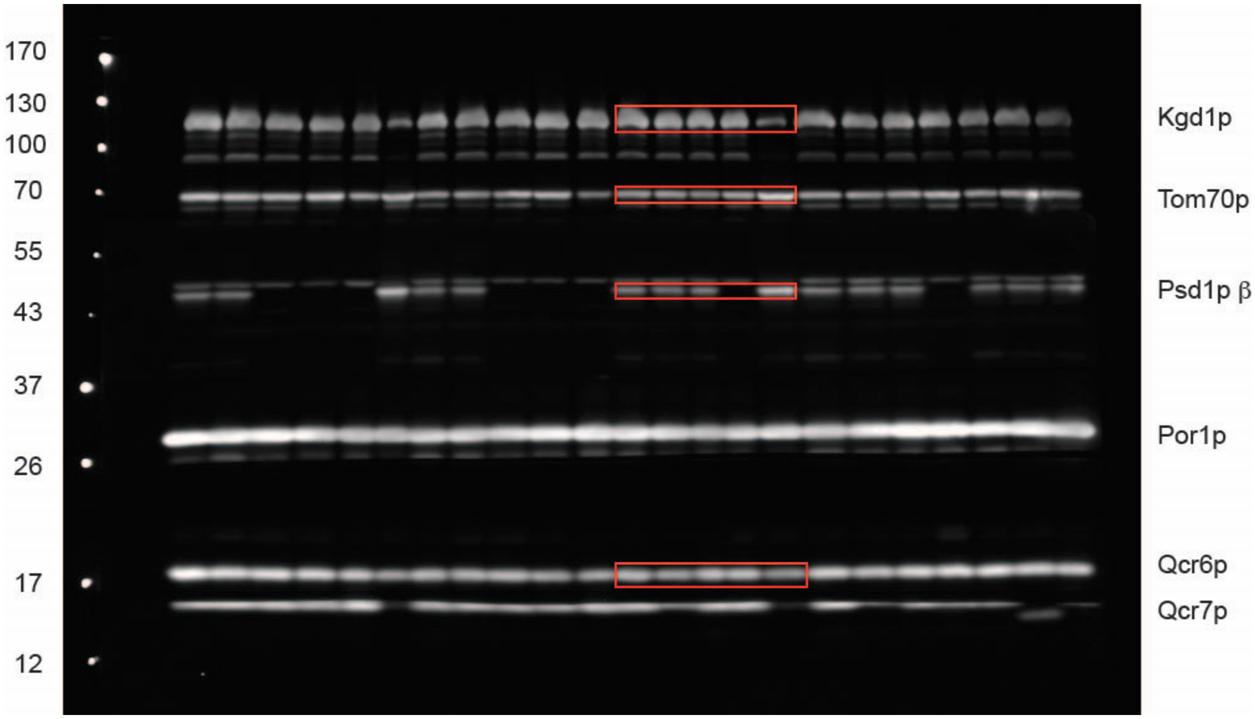

Blot 3 (Strip and Reprobe of above two immunoblots):

Composite exposures of: Atp2p (1min), Rip1p (1min), Aac2p (30sec), Qcr7p (2min)

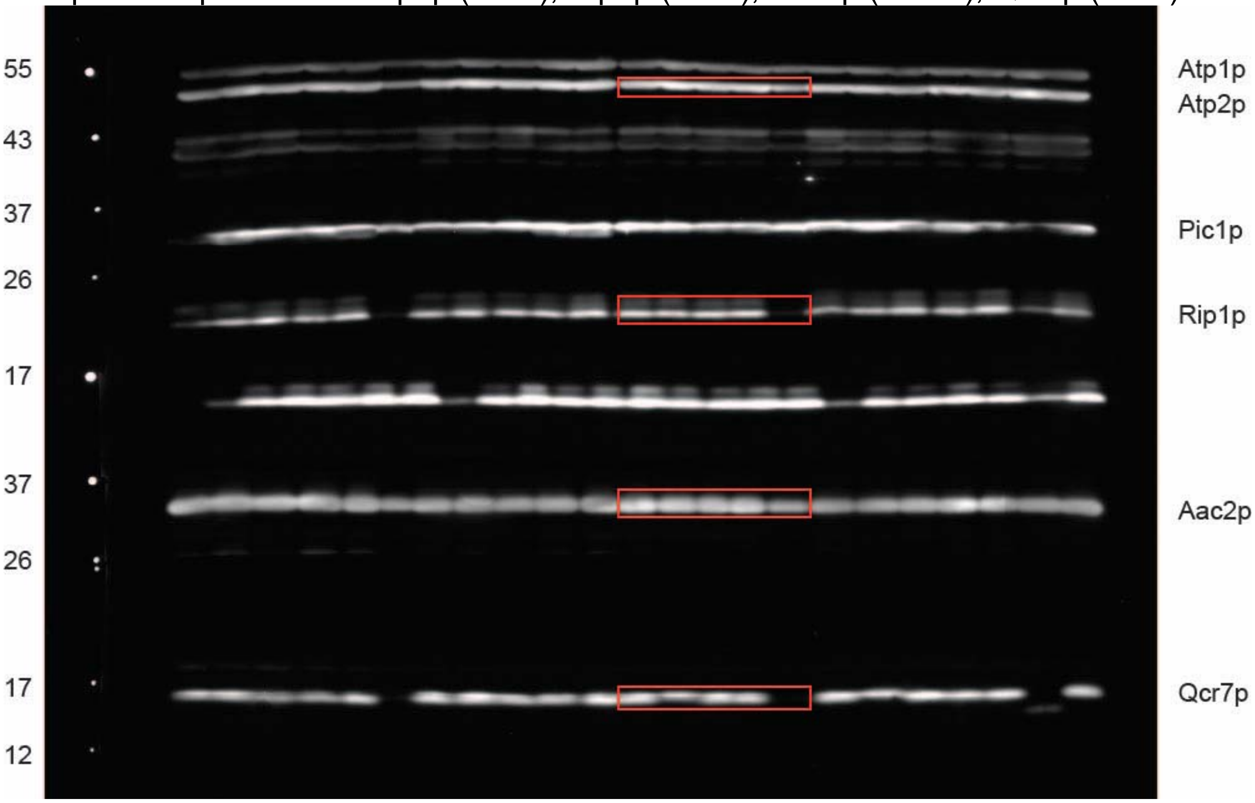

Fig. 9G Original uncropped file; unadjusted exposure:
Dated 2018_07_18 Rip1p 1D BNPAGE Qcr7p set
2min exposure of Rip1p

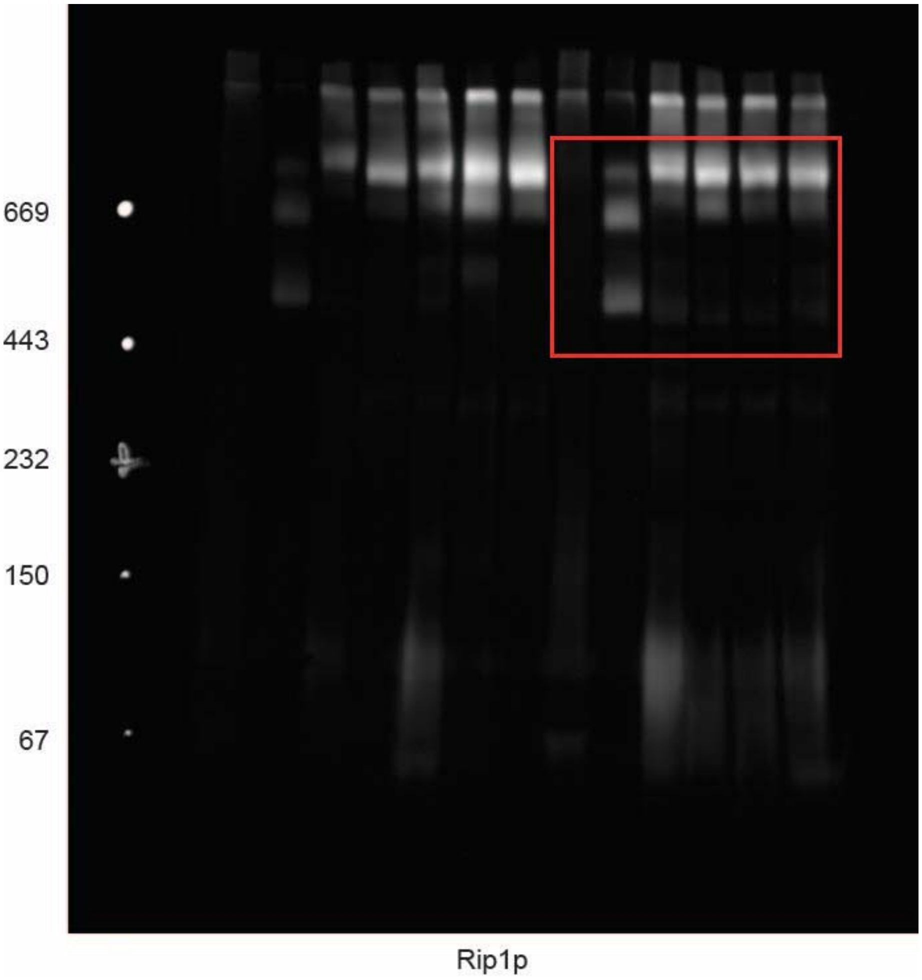

Fig. 9H Original uncropped file; unadjusted exposure:
Dated 2018_07_18 Cox4p 1D BNPAGE Qcr7p set
1min exposure of Cox4p

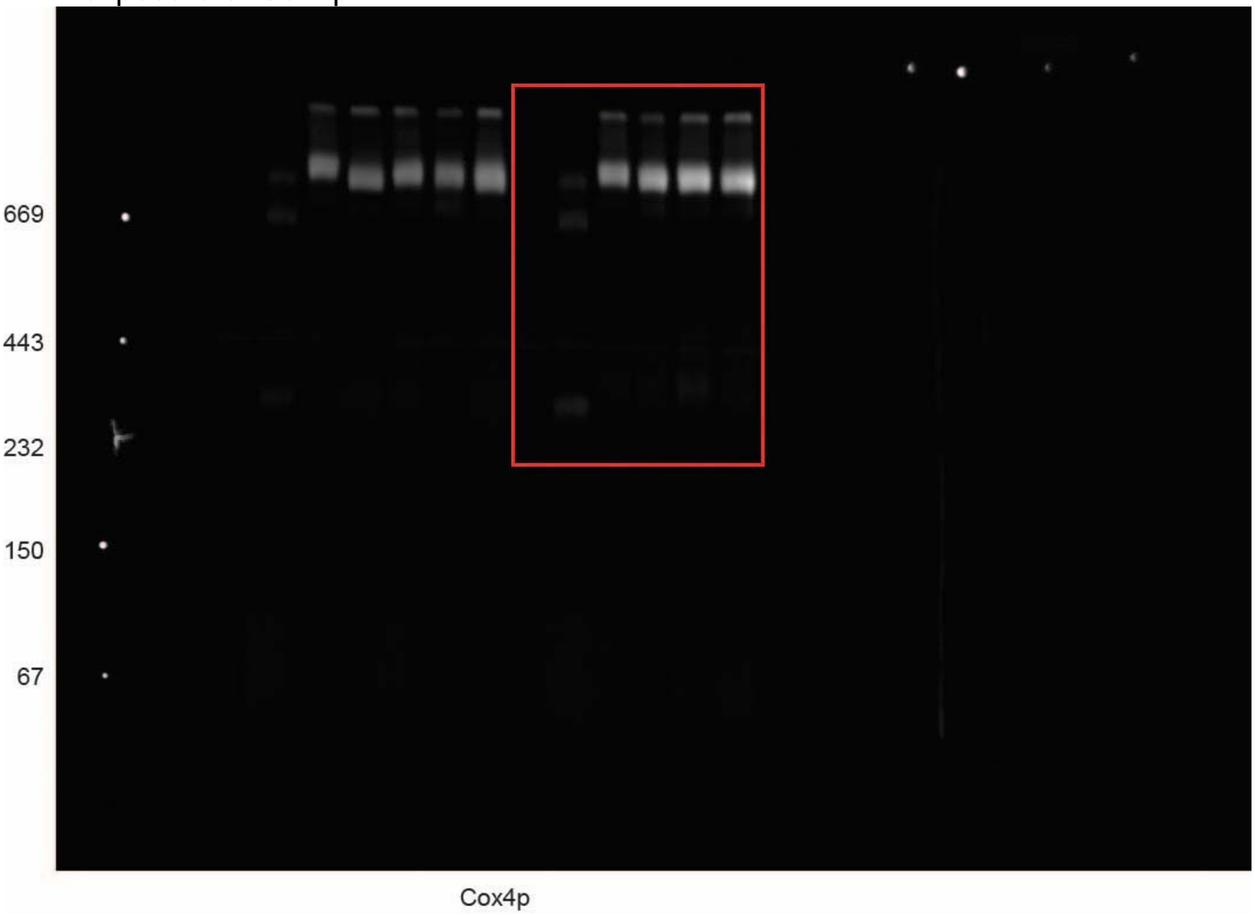

Supplementary Fig 1
Cell Fractionation boxed: Yellow IM-Psd1; Red ER-Psd1; blue OM-Psd1

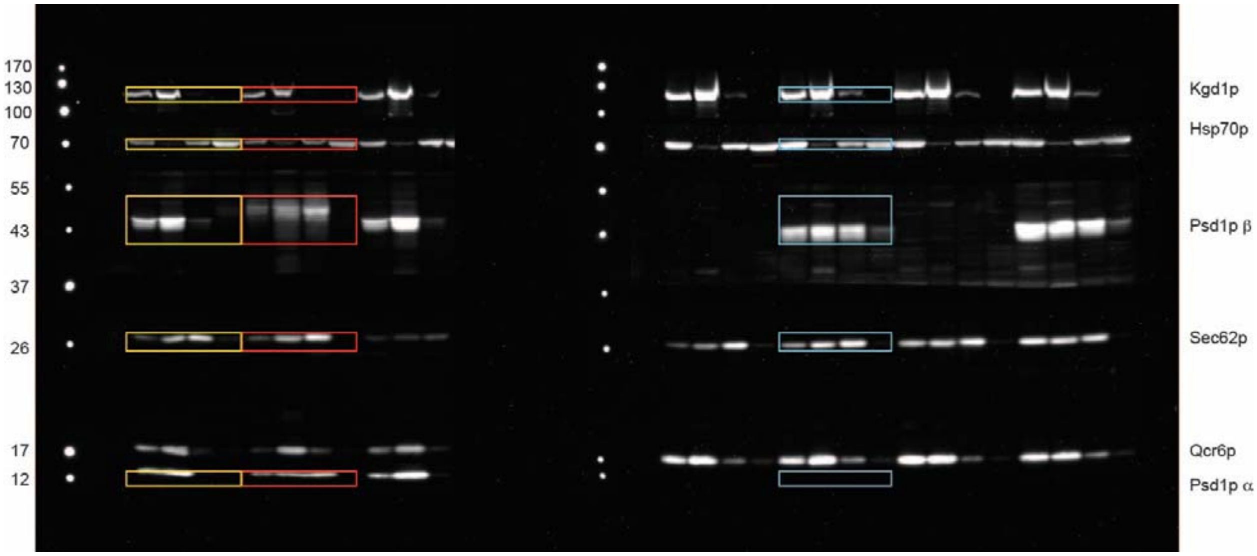

Supplementary Fig 2A

2018_02_28 Cho1p quantitation

Composite image of FluorChem Q exposures:
Kgd1p, Kar2p, Psd1p β, Cho1p, Psd1p α, Hxk1p

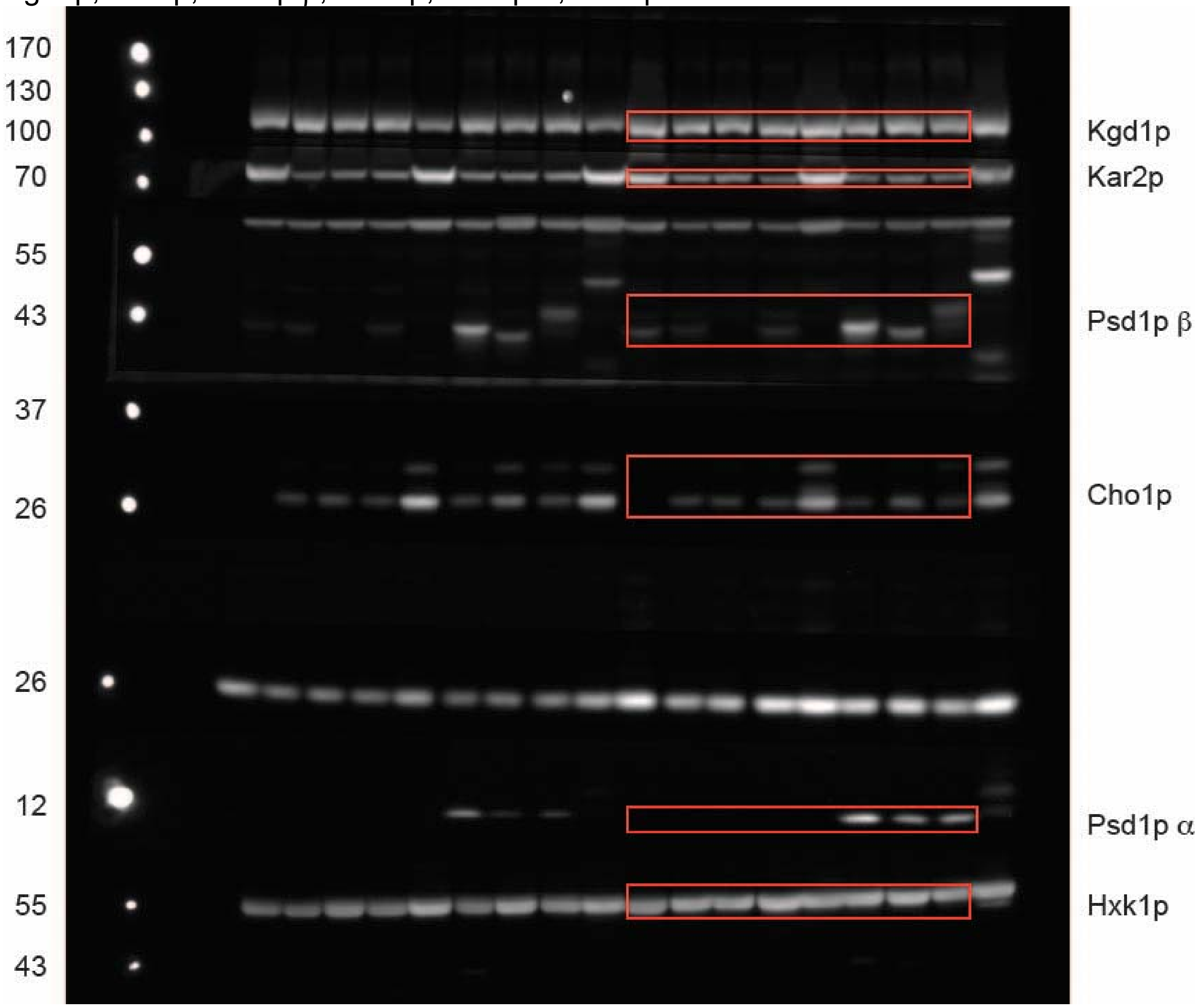

Supplementary Fig 8A

2017_12_14 YS n3 – Composite FluorChem Q exposures Pic1p (5sec), His (16min)

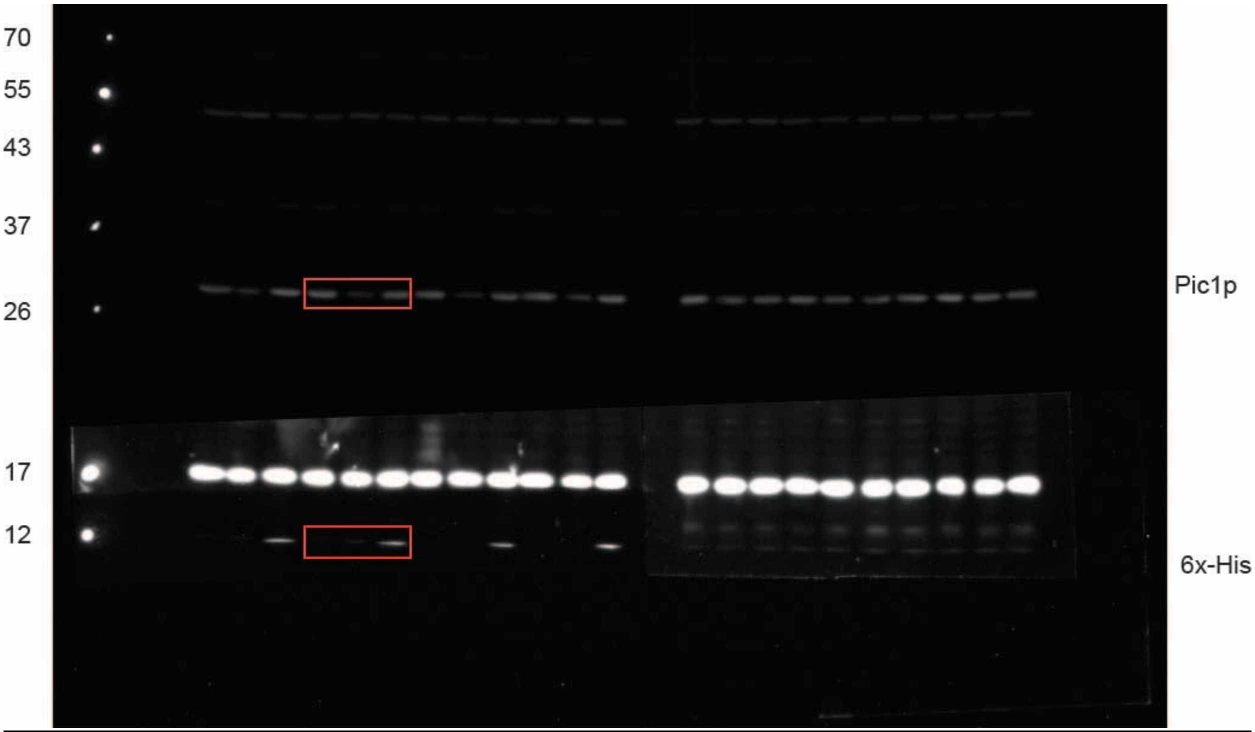

Supplementary Fig 8C

2018_01_09 1DNBPAGE n1 – Rip1p and Cox4p FluorChem Q exposures 1min

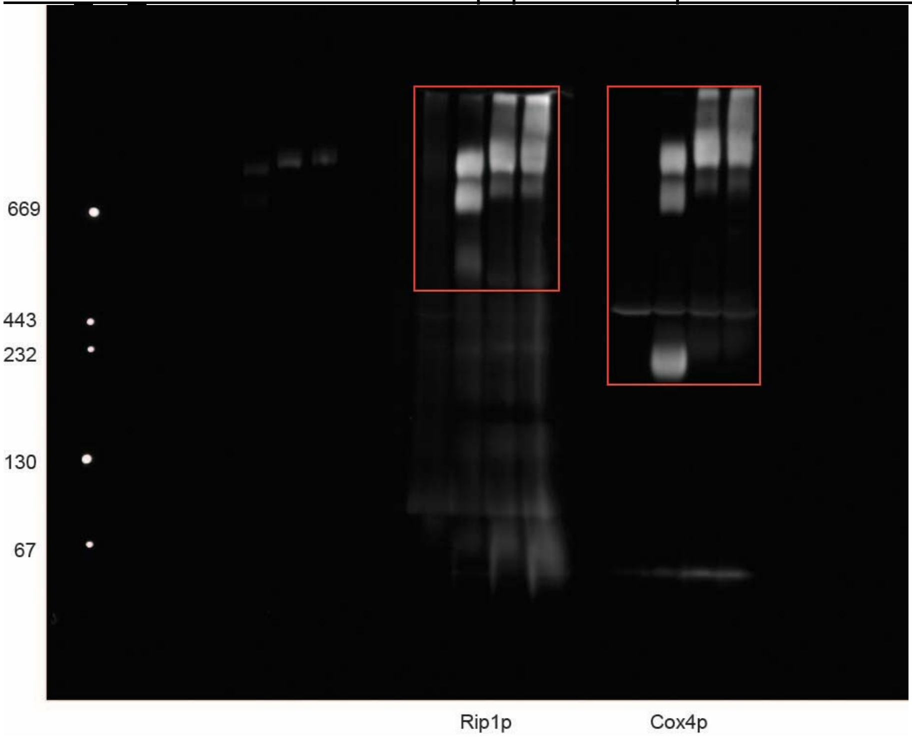

Supplementary Fig 8D

<u>2018_09_10 SMA IP – Composite FluorChem Q exposures</u>

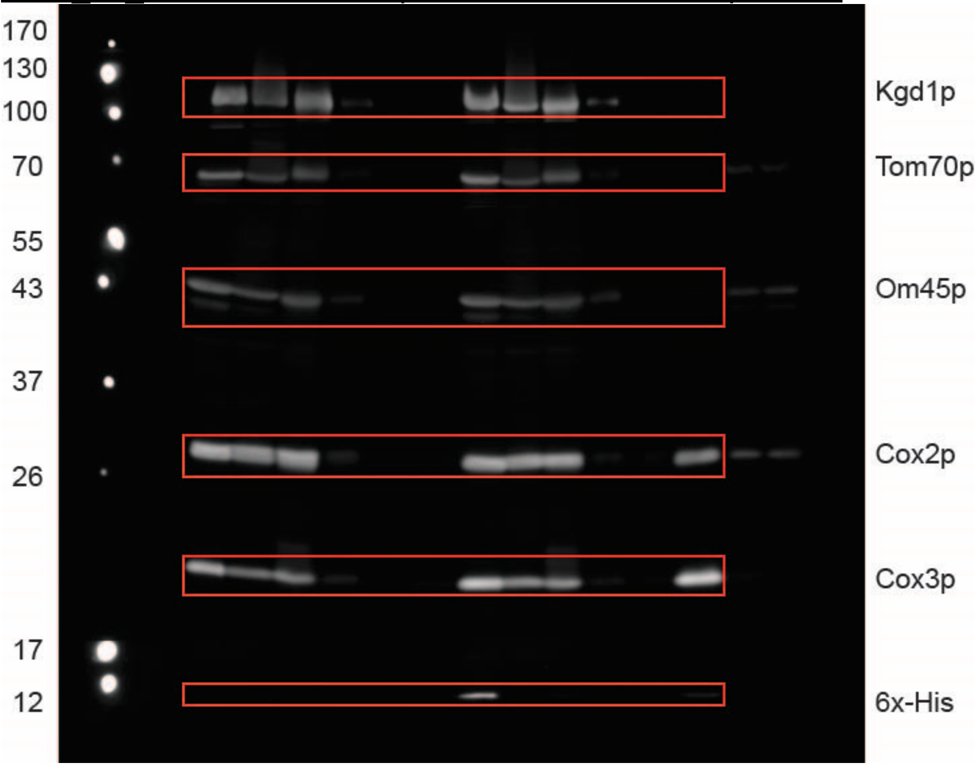

Supplementary Fig 8F

<u>2018_07_26 Steady State Mitos – Composite FluorChem Q exposures</u>

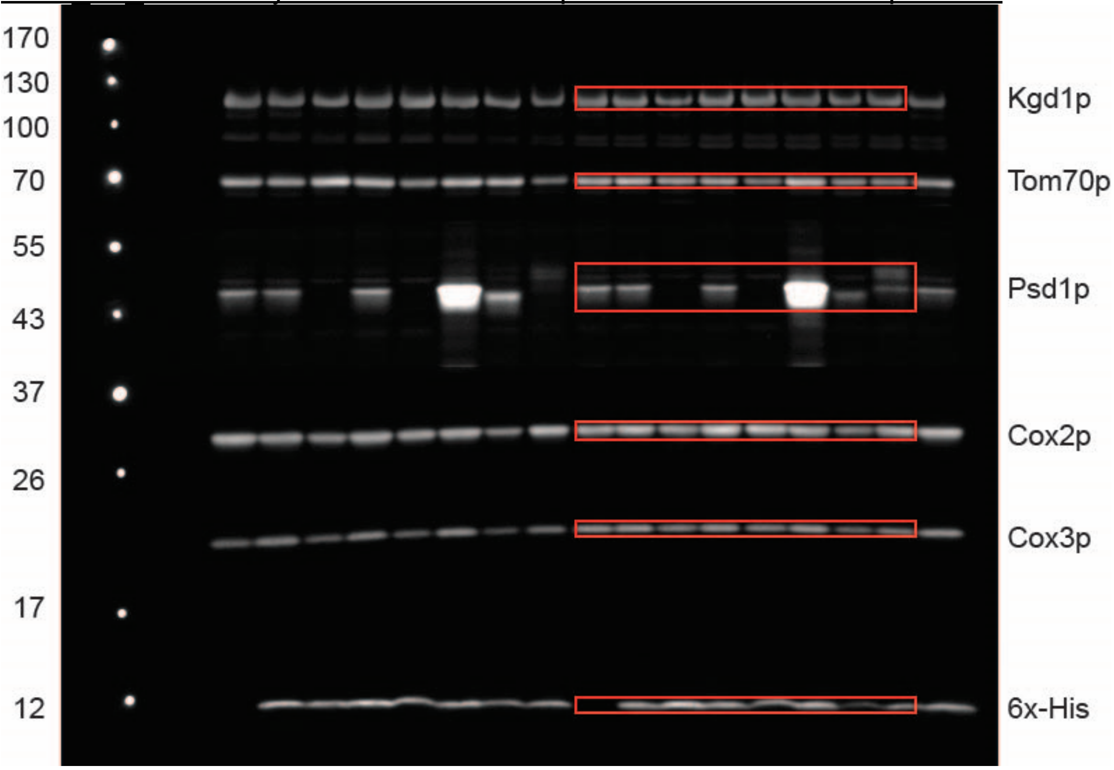

Supplement: Supplementary file 1 — Supplementary Information [file 41467_2019_9425_MOESM1_ESM.pdf]
